# Trans-Activation of the *Coactivator-Associated Arginine Methyltransferase 1* (*Carm1*) Gene by the Oncogene Product Tax of Human T-Cell Leukemia Virus Type 1

**DOI:** 10.3390/genes15060698

**Published:** 2024-05-27

**Authors:** Rahma F. Hayati, Rinka Nakajima, Yaxuan Zhou, Mashiro Shirasawa, Lin Zhao, Mariana Fikriyanti, Ritsuko Iwanaga, Andrew P. Bradford, Kenta Kurayoshi, Keigo Araki, Kiyoshi Ohtani

**Affiliations:** 1 Department of Biomedical Sciences, School of Biological and Environmental Sciences, Kwansei Gakuin University, 1 Gakuen Uegahara, Sanda 669-1330, Hyogo, Japan; rahma.idn@gmail.com (R.F.H.); hnj51097@kwansei.ac.jp (R.N.); gtk53096@kwansei.ac.jp (Y.Z.); idl05439@kwansei.ac.jp (M.S.); ght57978@kwansei.ac.jp (L.Z.); hsj19688@kwansei.ac.jp (M.F.); 2 Department of Obstetrics and Gynecology, University of Colorado School of Medicine, 12700 East 19th Avenue, Aurora, CO 80045, USA; ritsuko.iwanaga@cuanschutz.edu (R.I.); andy.bradford@cuanschutz.edu (A.P.B.); 3 Division of Molecular Genetics, Cancer Research Institute, Kanazawa University, Kakuma-machi, Kanazawa 920-1192, Ishikawa, Japan; kentakurayoshi@staff.kanazawa-u.ac.jp; 4 Department of Morphological Biology, Ohu University School of Dentistry, 31-1 Misumido Tomitamachi, Koriyama 963-8611, Fukushima, Japan; keigoaraki.res@gmail.com

**Keywords:** HTLV-1, tax, trans-activation, CARM1, epigenetic

## Abstract

Human T-cell leukemia virus type 1 (HTLV-1) is the causative agent of adult T-cell leukemia/lymphoma. The oncogene product Tax of HTLV-I is thought to play crucial roles in leukemogenesis by promoting proliferation of the virus-infected cells through activation of growth-promoting genes. These genes code for growth factors and their receptors, cytokines, cell adhesion molecules, growth signal transducers, transcription factors and cell cycle regulators. We show here that Tax activates the gene coding for coactivator-associated arginine methyltransferase 1 (CARM1), which epigenetically enhances gene expression through methylation of histones. Tax activated the *Carm1* gene and increased protein expression, not only in human T-cell lines but also in normal peripheral blood lymphocytes (PHA-PBLs). Tax increased R17-methylated histone H3 on the target gene *IL-2Rα*, concomitant with increased expression of CARM1. Short hairpin RNA (shRNA)-mediated knockdown of CARM1 decreased Tax-mediated induction of *IL-2Rα* and *Cyclin D2* gene expression, reduced E2F activation and inhibited cell cycle progression. Tax acted via response elements in intron 1 of the *Carm1* gene, through the NF-κB pathway. These results suggest that Tax-mediated activation of the *Carm1* gene contributes to leukemogenic target-gene expression and cell cycle progression, identifying the first epigenetic target gene for Tax-mediated trans-activation in cell growth promotion.

## 1. Introduction

Human T-cell leukemia virus type 1 (HTLV-1) is the causative agent of adult T-cell leukemia/lymphoma (ATL), HTLV-1-associated myelopathy (HAM)/tropical spastic paraparesis (TSP), and other inflammatory diseases [1,2,3,4,5,6]. Among the viral gene products, Tax and HTLV-1 basic zipper protein (HBZ) are thought to play crucial roles in the leukemogenesis [4,7]. Tax is a pleiotropic protein that interacts with a variety of cellular proteins such as transcription factors and signaling molecules, leading to the promotion of cell proliferation. These interactions facilitate Tax activation of cellular transcription factors, such as cAMP responsive element-binding factor (CREB), serum-responsive factor (SRF) and nuclear factor (NF)-κB, which lead to activation of not only the HTLV-1 promoter, in the long terminal repeat (LTR), but also cellular genes involved in cell proliferation [1]. Accumulating evidence indicates that activation of the NF-κB pathway by Tax is critical for both immortalization and proliferation of the virus-infected cells [1,8,9]. However, sustained activation of Tax induces cellular senescence, which can be prevented by HBZ attenuation of Tax functions, to permit persistent HTLV-1 viral infection [7,10,11,12,13,14]. Tax target genes identified to date are those encoding for growth factors and their receptors, cytokines, cell adhesion molecules, growth signal transducers, transcription factors and cell cycle regulators. We have previously reported that Tax directly activates cell-cycle regulatory genes such as *cyclin D2* and *cyclin-dependent kinase 6* (*cdk6*) via the NF-κB pathway, which leads to inactivation of the tumor suppressor pRB and activation of the transcription factor E2F that is essential for cell-cycle progression [15,16,17,18]. These observations suggest that activation of growth-promoting genes by Tax is the primary event for Tax-induced cell proliferation.

To better understand the roles of Tax in the promotion of cell proliferation, we screened for Tax-inducible genes in the human T-cell line Kit 225, using DNA microarray, and identified the *Coactivator-associated arginine methyltransferase 1* (*Carm1*) gene. CARM1, also known as protein arginine methyltransferase 4 (PRMT4), is a member of the PRMT family and regulates gene expression by methylating arginine (R) residues of histones, transcription factors and others [19]. Of note, CARM1 enhances methylation of R17 of histone H3, which serves as an epigenetic mark for transcriptional activation. Intriguingly, CARM1 has been reported to enhance transcriptional activation of HTLV-1 LTR by Tax through direct interaction with Tax [20]. This raises the possibility that CARM1 may also enhance Tax-mediated activation of cellular genes required for cell proliferation, and the consequent Tax-mediated cell cycle progression. We thus examined whether Tax activates the *Carm1* gene, and its effects on Tax-mediated target-gene expression and cell cycle progression.

## 2. Materials and Methods

### 2.1. Cell Culture

IL-2-dependent human T-cell line Kit 225 [21] was cultured in RPMI 1640 medium containing 10% fetal calf serum (FCS) and 0.5 nM IL-2 (Ajinomoto, Yokohama, Japan). To synchronize Kit 225 cells in resting state, the cells were cultured in the absence of IL-2 for 48 h. IL-2-independent human T-cell line Jurkat [22] and HTLV-1-infected human T-cell lines MT-1 [23], TL-Om1 [24], HuT 102 [25], MT-2 [26], MT-4 [27] and TL-Su [28] were maintained in RPMI 1640 medium containing 10% FCS. Peripheral blood lymphocytes (PBLs) were obtained from a healthy donor, isolated by density gradient using Ficoll-Paque PLUS (Amersham Pharmacia Biotech, Amersham, UK) and cultured in RPMI 1640 medium containing 20% FCS and 0.15% phytohemagglutinin (PHA) for 72 h, generating PHA-stimulated PBLs (PHA-PBLs).

### 2.2. Plasmids

pCARM1-Luc(-1564) was generated by cloning the -1564 to +143 region of CARM1 promoter into the *Kpn*I and *Hin*dIII sites of pGL3-Basic (Promega, Madison, WI, USA). pCARM1DS-Luc was generated by cloning the +522 to +1490 region in the 1st intron of the *Carm1* gene into the *Kpn*I and *Xho*I sites of pGL3-Promoter (Promega). pENTR-shCARM1-1~pENTR-shCARM1-4 were generated by replacing the control sequence of pENTR-shCon with shRNA sequences against CARM1 (listed in RNA interference). pGP34-Luc(-823) was generated by subcloning the -823 to +27 region of the GP34 promoter from pGP34(-823)CAT into pGL3-Basic (Promega) [29]. pLTR-Luc, pE2WTx4-Luc, pκB-Luc, pCycD2-Luc(-1624), pMT2-Tax, pHβAPr-1-neo, pCMV-β-gal, pENTR-shCon, pENTR-shp65, pENTR-shp100-1, 2 and shRNA-resistant p65 expression vector pENTR-CMV-p65pm were described previously [15,18].

### 2.3. Transfection and Reporter Assay

Reporter plasmids and expression vectors were transfected into the asynchronously growing Kit 225 or Jurkat cells using the DEAE-dextran method as described previously [16]. pCMV-β-gal was included as an internal control. The cells were cultured in the absence of IL-2 for 48 h (Kit 225 and Jurkat with shCARM1) or 24 h (Jurkat with Tax alone), and luciferase activities were determined as described previously [30]. All assays were carried out in triplicate and results are presented as means ± SD.

### 2.4. DNA Microarray

Kit 225 cells were infected with Tax-expressing recombinant adenovirus (AxCAIY-Tax) or control virus (Ad-IWI), cultured for 2 days in the absence of IL-2, and harvested. Total RNA was isolated and preliminary analysis was carried out using Human Chip Version 1, which was under development at the time (DNA Chip Research Institute Inc., Tokyo, Japan).

### 2.5. Recombinant Adenovirus

Recombinant adenovirus expressing Tax (AxCAIY-Tax) and control virus (Ad-IWI), and Ad-shCon were described previously [15,18]. Ad-shCARM1-1~Ad-shCARM1-4 were generated from pENTR-shCARM1-1~pENTR-shCARM1-4, using the ViraPower Adenoviral Expression System (Invitrogen, Carlsbad, CA, USA), according to the supplier’s protocol. The titer of purified viruses was determined by serial dilution and infection of 293A cells followed by staining the *E2* gene product with the specific antibody raised against synthetic peptides in rabbit (a kind gift from Dr. M. Ikeda, Tokyo Medical and Dental University). Infection was carried out as described previously [15].

### 2.6. RNA Interference

Target sequences of shRNA against CARM1 (shCARM1) were as follows.

shCARM1-1: 5′-GTACACGGTGAACTTCTTA-3′

shCARM1-2: 5′-CTTCTTAGAAGCCAAAGAA-3′

shCARM1-3: 5′-GGATAGAAATCCCATTCAA-3′

shCARM1-4: 5′-GTCTTTAAGTGCTCAGTGTCC-3′

### 2.7. Quantitative Reverse Transcription (qRT)-PCR

Total RNA was extracted using Isogen II (Nippon Gene). First-strand cDNA was synthesized using the First Strand cDNA Synthesis Kit for RT-PCR [AMV] (Roche) using oligo (dT) primer. Quantitative PCR was carried out using KAPA SYBR qPCR Mix (KAPA Biosystems, Wilmington, MA, USA) and Thermal Cycler Dice Real Time System Single (TaKaRa, Middleton, WI, USA). Specific primer sets used were as follows.

*Carm 1*

Fw: 5′-CACGGCCTGGCTTTCTGGTTTGAC-3′

Rv: 5′-CATCCCGCTGCTGAGGTTGTAGGT-3′

*Cyclin D2*

Fw: 5′-CTGTGTGCCACCGACTTTAGTT-3′

Rv: 5′-GATGGCTGGCCCACACTTC-3′

*IL-2Rα*

Fw: 5′-GTGGTGGGGCAGATGGTTTATTAT-3′

Rv: 5′-TGTCTGTTCCCGGCTTCTTACCAA-3′

Specific primer set for *GAPDH* was described previously [31].

### 2.8. Fluorescence-Activated Cell Sorting (FACS) Analysis

FACS analysis was carried iout as described previously [31]. Cells were fixed with 70% ethanol and stained with propidium iodide (50 μg/mL) containing RNase (50 μg/mL). Cell samples were analyzed with a FACSCalibur (Becton Dickinson, Franklin Lakes, NJ, USA).

### 2.9. Immunoblot Analysis

Immunoblot analysis was carried out as described previously [16]. The antibodies used were anti-Tax antibody Lt-4 [7], anti-Cyclin D2 (sc-593, Santa Cruz Biotechnology, Dallas, TX, USA, 1:1000), anti-CARM1 (12495S, Cell Signaling, Danvers, MA, USA, 1:1000), anti-β-actin (A1978, SIGMA, Burlington, MA, USA, 1:2000), peroxidase-conjugated anti-mouse IgG (NA9310, Amersham, Buckinghamshire, UK, 1:5000) and peroxidase-conjugated anti-rabbit IgG (NA934, Amersham, 1:5000).

### 2.10. Chromatin Immunoprecipitation (ChIP) Assay

The ChIP assay was carried out as described previously [31]. Antibodies used were anti-HisH3R17m2 (ab8284, abcam, Waltham, MA, USA), anti-CARM1 (12495S, Cell Signaling) and anti-HA (sc-7392, Santa Cruz Biotechnology) as a negative control. Gene-specific primer sets used were as follows.

*IL-2Rα*

Fw: 5′-CTGGGAAGTTGGAATGAGATGAA-3′

Rv: 5′-TAAGAAGCCGGGAACAGACAACAG-3′

*β-actin*

Fw: 5′-AGTGGGGTGGCTTTTAGGATGG-3′

Rv: 5′-TGCGCAGAAAACAAGATGAGATT-3′

The *β-actin* gene was used as a negative control. Input was 1/60 of the lysates.

### 2.11. Statistical Analysis

Reporter assays were carried out in triplicate. Data are presented as means ± SD. Statistical comparisons were made using Student’s *t*-test and Bonferroni correction. *p* value <0.05 was considered as significant.

## 3. Results

### 3.1. Tax induces Carm1 Gene Expression in Human T-Cells

To identify novel Tax target genes, we searched for Tax-inducible genes in the human T-cell line, Kit 225, using DNA microarray. Kit 225 cells are dependent on IL-2 for cell growth and can be arrested by deprivation of IL-2. Subsequent expression of Tax in IL-2-starved Kit 225 cells can induce cell cycle progression [15]. Kit 225 cells were infected with recombinant adenovirus expressing Tax or control virus, cultured for 2 days in the absence of IL-2 and harvested. Tax-inducible genes were screened by DNA microarray (Supplementary Tables S1–S3). Top 300 genes, which were induced by Tax, are listed in Supplementary Tables S1–S3 (100 genes including control genes for each Table) in the order of degree of induction. These genes include previously reported Tax targets such as baculoviral IAP repeat-containing 3 (cIAP2) [32], tumor necrosis factor (ligand) superfamily, member 4 (tax-transcriptionally activated glycoprotein 1, 34 kD) [33], signal transducer and activator of transcription 5 (STAT5) [34], early growth response 1 and early growth response 2 [35], BCL2-related protein A1 (BFL1) [36], granulocyte-macrophage colony stimulating factor 2 [37], thioredoxin [38], cyclin-dependent kinase inhibitor 1A (p21^Cip1^) [39], intercellular adhesion molecule 1 (CD54) [40], v-jun avian sarcoma virus 17 oncogene homolog (c-jun) [41], vimentin [42], proliferating cell nuclear antigen [43], fascin [44], nuclear receptor subfamily 4, group A, member 1 (TR3, Nur77) [45], tumor necrosis factor receptor superfamily, member 9 (4–1BB) [46], tumor necrosis factor ligand superfamily, member 7 (CD70) [47], CD44 [48], Pim-1 [49], Cyclin D1 [50] and major histocompatibility complex, class I [51]. These observations support the validity of the screening. Among these genes, we found and focused on the *Carm1* gene (Rank 55 in Supplementary Table S1), since epigenetic genes have not been identified previously as Tax targets.

**Figure 1:**
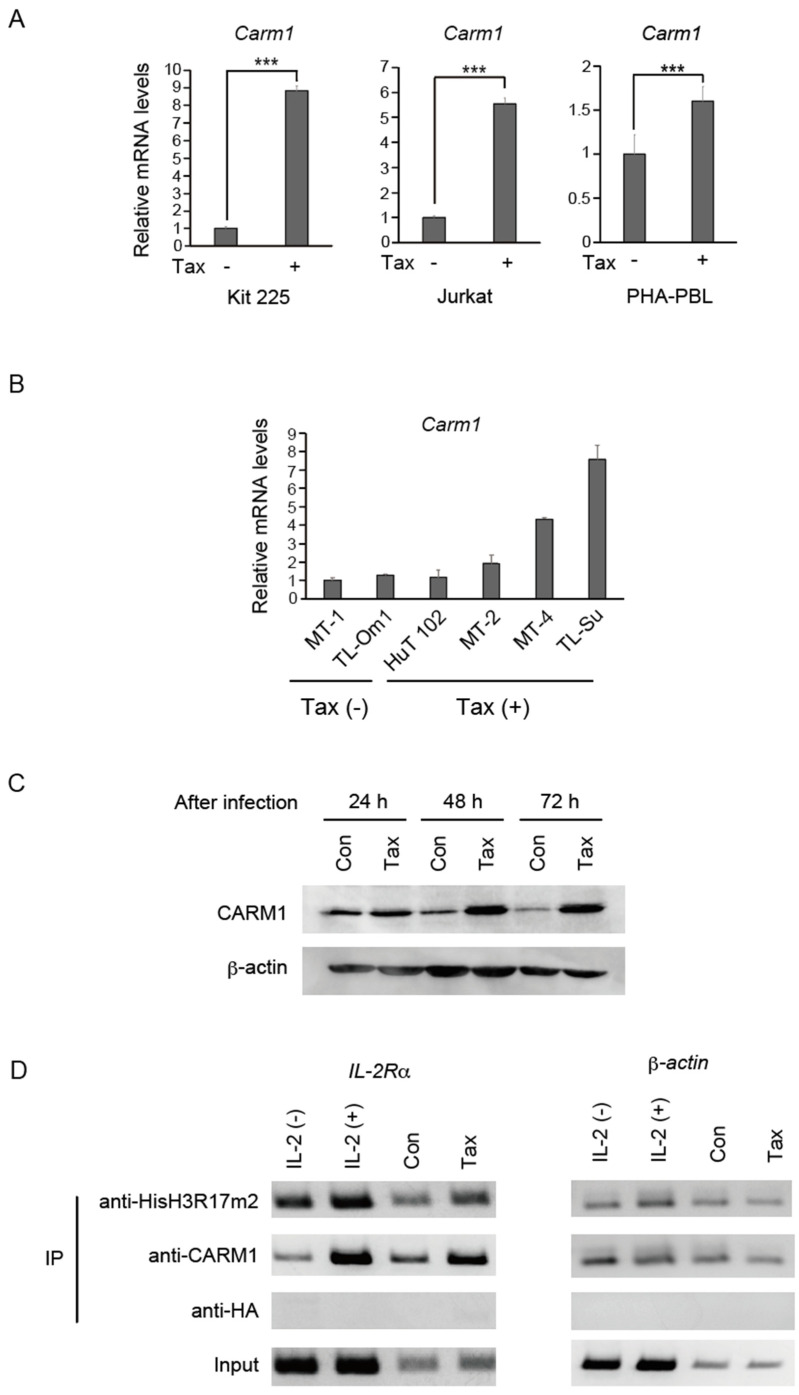
Tax induces CARM1 expression in T-cells. (**A**) Tax induced *Carm1* gene expression in T-cells. Kit 225 and Jurkat cells were infected with recombinant adenovirus expressing Tax (MOI 200) and further cultured for 48 h in the absence of IL-2. PHA-PBLs were similarly infected with Tax-expressing virus and further cultured for 72 h in the absence of IL-2. The cells were harvested and the levels of *Carm1* mRNA were measured by qRT-PCR and adjusted by that of *GAPDH* as an internal control. Fold inductions by Tax are indicated. *** *p* < 0.01. (**B**) *Carm1* gene expression tends to be higher in Tax-expressing cells than in non-expressing cells. *Carm1* gene expression was examined in indicated HTLV-1-infected T-cell lines by qRT-PCR and normalized by that of *GAPDH* as an internal control. Relative expression levels are presented. (**C**) Tax induced CARM1 protein expression in T-cells. Kit 225 cells were infected with Tax-expressing adenovirus, further cultured for 24, 48 and 72 h in the absence of IL-2, and harvested. Levels of CARM1 protein were examined by Western blot analysis. β-actin was used as an internal control. (**D**) Tax induced methylation of R17 of histone H3 on the *IL-2R*α gene. Kit 225 cells were starved of IL-2, restimulated with IL-2 or infected with Tax-expressing adenovirus, further cultured for 24 h, and ChIP assay was performed using anti-HisH3R17m2, anti-CARM1 or anti-HA antibody as a negative control. The immunoprecipitated *IL-2R*α gene fragment was amplified using a specific primer set. The *β-actin* gene was used as a negative control.

To confirm that Tax induces *Carm1* gene expression in the human T-cell line Kit 225, we introduced Tax by recombinant adenovirus-mediated gene transfer and examined the level of *Carm1* gene expression by qRT-PCR. The efficiency of recombinant adenovirus-mediated gene transduction into Kit 225 cells is about 65% [15]. Expression of the *Carm1* gene was induced about 9-fold (Figure 1A left panel), indicating that Tax can enhance *Carm1* gene expression in resting Kit 225 cells. However, the induction of *Carm1* gene expression could be a consequence of Tax stimulation of cell cycle progression. Thus, we examined whether Tax can induce *Carm1* gene expression in Jurkat cells, which are not dependent on IL-2 for cell growth. The introduction of Tax into asynchronously growing Jurkat cells induced *Carm1* gene expression about 5.5-fold (Figure 1A, middle panel), suggesting that activation of *Carm1* gene expression by Tax is not a secondary consequence of Tax-mediated cell cycle progression. We also determined the effect of Tax on *Carm1* gene expression in normal T-cells, using phytohemagglutinin-stimulated peripheral blood lymphocytes (PHA-PBLs). The efficiency of recombinant adenovirus-mediated gene transduction into PHA-PBLs is about 45% [10]. Tax also increased *Carm1* gene expression in PHA-PBLs about 1.6-fold (Figure 1A right panel), suggesting that Tax regulates *Carm1* gene expression in normal T-cells.

We also examined whether there is correlation between *Carm1* gene expression and Tax expression in HTLV-1-infected T-cell lines. MT-1 and TL-Om1 scarcely express Tax, whereas HuT 102, MT-2, MT-4 and TL-Su express Tax. The levels of *Carm1* mRNA were examined in these cell lines and normalized by that of *GAPDH* as an internal control (Figure 1B). Tax-expressing MT-2, MT-4 and TL-Su showed higher levels of *Carm1* expression than non-expressing MT-1 and TL-Om1, supporting the notion that Tax facilitates *Carm1* gene expression.

Finally, we examined whether CARM1 protein is induced by Tax, by Western blot analysis, in IL-2-starved Kit 225 cells. The level of CARM1 protein was clearly enhanced in a time-dependent manner (Figure 1C), indicating that Tax-mediated induction of *Carm1* gene expression is also reflected at the protein level.

Taken together, these results indicate that Tax induces *Carm1* gene expression in human T-cells.

### 3.2. Tax Induces Methylation of Histone H3 on Target Genes

CARM1 stimulates gene expression by methylating histones, such as R17 of histone H3, generating a positive mark for epigenetic regulation. We thus examined whether Tax expression enhanced histone methylation on its target genes, assessed by chromatin immunoprecipitation (ChIP). Kit 225 cells were starved of IL-2 and transduced with Tax, by recombinant adenovirus-mediated gene transfer, or restimulated with IL-2 as a positive control. ChIP assay was performed using an antibody that specifically recognizes histone H3 di-methylated at R17 (HisH3R17m2), which is known to be mediated by CARM1 (Figure 1D). The results show that not only IL-2 stimulation, but also Tax, increased the methylation of histone H3R17 on the Tax target gene *IL-2R*α, suggesting that induction of CARM1 by Tax and consequent H3 methylation may contribute to the activation of target genes by Tax.

### 3.3. Induction of Carm1 Gene Expression Contributes to Tax-Mediated Target-Gene Expression and Cell Cycle Progression

**Figure 2:**
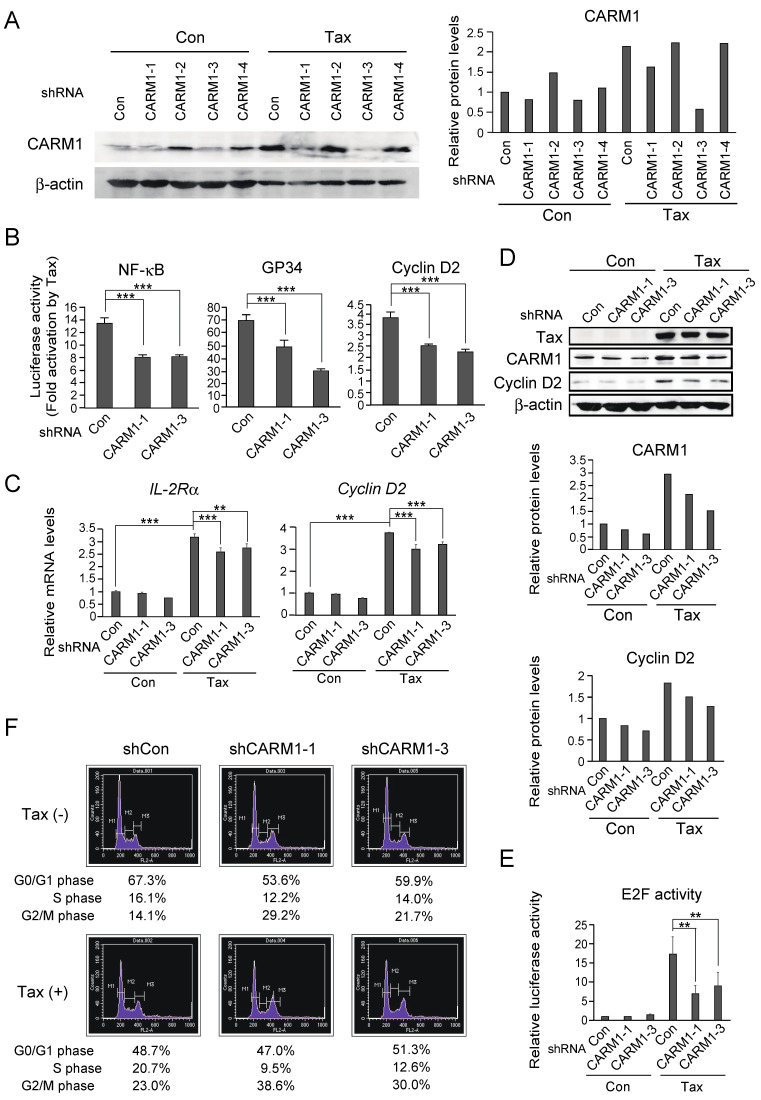
Knockdown of CARM1 expression reduces Tax-mediated target-gene induction and cell cycle progression. (**A**) Knockdown effects of shRNAs against CARM1. Kit 225 cells were infected with recombinant adenovirus expressing shRNA against CARM1 with (Tax) or without (Con) Tax-expressing adenovirus, further cultured for 48 h in the absence of IL-2, and harvested. The levels of CARM1 protein were examined by Western blot analysis (left panel). β-actin was used as an internal control. The intensities of bands were measured by ImageJ, and CARM1 protein levels were normalized by using that of β-actin as an internal control. Relative protein levels are presented (right panel). (**B**) Knockdown of CARM1 reduced Tax-mediated activation of target promoters. Kit 225 cells were transfected with pκB-Luc, pGP34-Luc or pCycD2-Luc and Tax expression vector, along with expression vector for shRNA against CARM1 or control vector. pCMV-β-gal was included as an internal control. The cells were further cultured for 48 h in the absence of IL-2, and harvested. Luciferase activities were measured and adjusted by β-galactosidase activities. Fold activations by Tax are shown. *** *p* < 0.01. (**C**) Knockdown of CARM1 reduced Tax-mediated induction of target-gene expression. Kit 225 cells were infected with control virus or Tax-expressing adenovirus along with adenovirus expressing shRNA against CARM1 or control virus. The cells were further cultured in the absence of IL-2 for 48 h, and harvested. The levels of *IL-2R*α and *Cyclin D2* mRNAs were measured by qRT-PCR and adjusted by using that of *GAPDH* as an internal control. Relative expression levels are shown. ** 0.01 ≦ *p* < 0.05, *** *p* < 0.01. (**D**) Knockdown of CARM1 reduced Tax-mediated induction of Cyclin D2 expression. Under the same condition, expression levels of Cyclin D2 were examined by Western blot analysis (upper panel). β-actin was used as an internal control. Knockdown of CARM1 was also confirmed. The intensities of bands of CARM1 and Cyclin D2 were measured by ImageJ and normalized by that of β-actin, which was used as an internal control. Relative protein levels of CARM1 and Cyclin D2 are presented (middle and lower panels). (**E**) Knockdown of CARM1 reduced Tax-mediated activation of E2F. Kit 225 cells were transfected with pE2WTx4-Luc with the Tax expression vector or control vector, along with the expression vector for shRNA against CARM1 or the control vector. pCMV-β-gal was included as an internal control. The cells were cultured in the absence of IL-2 for 48 h, and harvested. Luciferase activities were measured and adjusted by β-galactosidase activities. Relative luciferase activities are shown. ** 0.01 ≦ *p* < 0.05. (**F**) Knockdown of CARM1 reduced Tax-mediated promotion of cell cycle progression. Asynchronously growing Kit 225 cells were infected with Tax-expressing adenovirus (Tax(+)) or control virus (Tax(−)), along with adenovirus expressing shRNA against CARM1. The cells were cultured in the absence of IL-2 for 48 h, and cell cycle distribution was examined by FACS analysis with DNA content as an indicator. Percent populations of cells in G0/G1, S and G2/M phases are indicated.

We next explored whether down-regulation of Tax-mediated induction of CARM1 expression suppressed Tax stimulation of target-gene expression and cell cycle progression. For this purpose, we utilized shRNA mediated knockdown of CARM1 expression, constructing expression vectors and recombinant adenovirus expressing shRNA against four different target sequences of the *Carm1* gene. We first examined knockdown effects of shRNA against CARM1 (shCARM1), in the absence and presence of Tax expression in IL-2-starved Kit 225 cells, by Western blot analysis (Figure 2A). A reduction in CARM1 expression was observed with shCARM1-1 and -3 both in the absence and presence of Tax. We thus used shCARM1-1 and -3 for further analyses to exclude off-target effects. Activation of the NF-κB pathway is critical for Tax-mediated induction of Cyclin D2 expression and cell cycle progression in IL-2-starved Kit 225 cells [7,8,9,10]. Thus, we examined whether knockdown of CARM1 reduced Tax-mediated activation of an NF-κB reporter and the GP34 and Cyclin D2 promoters, which are also activated by Tax, primarily via the NF-κB pathway. The introduction of shCARM1-1 and -3 reduced Tax activation of the NF-κB reporter and the GP34 and Cyclin D2 promoters (Figure 2B). These results suggest that CARM1 contributes to Tax-mediated activation of target-gene promoters through the NF-κB pathway, a critical mediator of Tax-dependent promotion of cell growth.

We next examined whether knockdown of CARM1 expression compromised Tax-mediated induction of target genes. Kit 225 cells were infected with Tax-expressing adenovirus, along with shCARM1-expressing virus. The cells were cultured in the absence of IL-2, and the expression of Tax target genes *IL2R*α and *Cyclin D2* was examined by qRT-PCR. The introduction of shCARM1 significantly reduced Tax-mediated induction of both *IL-2R*α and *Cyclin D2* gene expression (Figure 2C). Knockdown of CARM1 slightly reduced *IL-2R*α and *Cyclin D2* gene expression in the absence of Tax. This could be due to knockdown of basal-level expression of CARM1. Although the effects of knockdown of CARM1 in the presence of Tax were modest, they were much bigger than in the absence of Tax. In addition, gene transduction efficiency of Kit 225 cells by recombinant adenovirus is about 65% [15]. Thus the effect is expected to be much bigger in the gene-transduced cells. A reduction in Cyclin D2 expression was confirmed at the protein level by Western blot analysis, along with that of CARM1 (Figure 2D). These results suggest that Tax-mediated induction of CARM1 expression contributes to Tax-dependent activation of target genes.

Finally, we examined the effect of CARM1 knockdown on Tax-mediated activation of E2F, which is crucial for cell cycle progression. Kit 225 cells were transfected with an E2F reporter plasmid and Tax expression vector, along with an expression vector for shRNA against CARM1, and cultured in the absence of IL-2 to reduce endogenous E2F activity. The introduction of Tax activated the E2F reporter about 17-fold, while knockdown of CARM1 significantly reduced Tax-mediated activation of the E2F reporter, 5- to 6-fold (Figure 2E). This suggests that Tax-dependent induction of CARM1 contributes to Tax-mediated cell cycle progression. We thus examined the effect of CARM1 knockdown on Tax stimulation of cell cycle progression. Kit 225 cells were infected with Tax-expressing adenovirus or control virus, along with adenovirus expressing shRNA against CARM1 and cultured for 48 h in the absence of IL-2 to achieve quiescence. CARM1 knocked-down cells showed a much lower percentage of cells in S phase in Tax-expressing cells than in control cells (Figure 2F), indicating that Tax induction of CARM1 is a critical component of Tax-dependent regulation of cell cycle progression.

### 3.4. Tax Activation of Carm1 Requires Sequences in the First Intron of the Carm1 Gene

**Figure 3:**
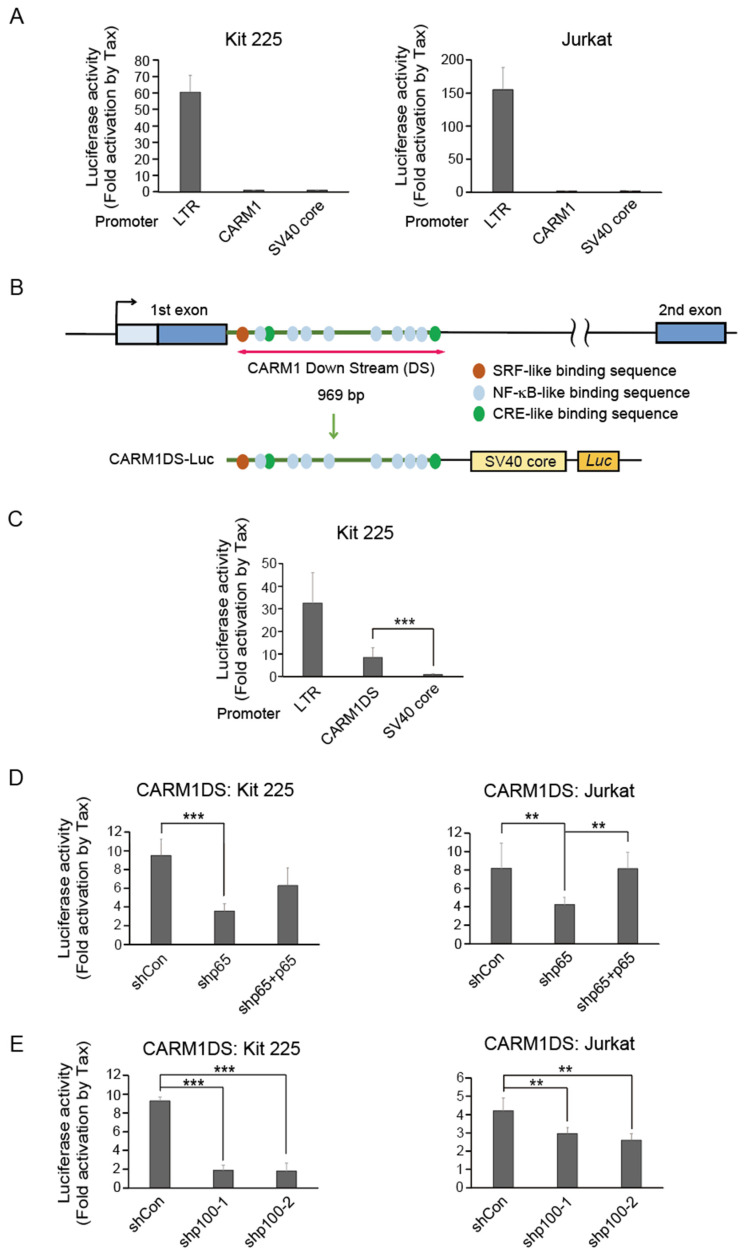
Tax acts via sequences in the first intron of the *Carm1* gene. (**A**) Tax did not activate the upstream regulatory region of the *Carm1* gene. Kit 225 or Jurkat cells were transfected with pCARM1-Luc with the Tax expression vector or control vector, along with pCMV-β-gal as an internal control. pLTR-Luc and pGL3-Promoter were used as positive and negative controls, respectively. The cells were cultured in the absence of IL-2 for 48 h (Kit 225) or 24 h (Jurkat), and harvested. Luciferase activities were measured and adjusted by β-galactosidase activities. Fold activations by Tax are shown. (**B**) Schematic presentation of downstream sequences in the first intron of the *Carm1* gene (CARM1DS). (**C**) Tax activated sequences in the first intron of the *Carm1* gene. Kit 225 cells were transfected with pCARM1DS-Luc with the Tax expression vector or control vector, along with pCMV-β-gal as an internal control. pLTR-Luc and pGL3-Promoter were used as positive and negative controls, respectively. The cells were cultured in the absence of IL-2 for 48 h, and harvested. Luciferase activities were measured and adjusted by β-galactosidase activities. Fold activations by Tax are shown. *** *p* < 0.01. (**D**) NF-κB p65 (RelA) is involved in activation of CARM1DS by Tax. Kit 225 or Jurkat cells were transfected with pCARM1DS-Luc with the Tax expression vector or control vector, along with expression vector for shRNA against p65 with or without expression vector for shRNA-resistant p65 expression vector. pCMV-β-gal was included as an internal control. The cells were cultured in the absence of IL-2 for 48 h, and harvested. Luciferase activities were measured and adjusted by β-galactosidase activities. Fold activations by Tax are shown. ** 0.01 ≦ *p* < 0.05, *** *p* < 0.01. (**E**) NF-κB p100 (precursor of p52, heterodimeric partner of RelB) is involved in activation of CARM1DS by Tax. Kit 225 or Jurkat cells were transfected with pCARM1DS-Luc with the Tax expression vector or control vector, along with expression vectors for shRNA against p100 with two different target sequences. pCMV-β-gal was included as an internal control. The cells were cultured in the absence of IL-2 for 48 h, and harvested. Luciferase activities were measured and adjusted by β-galactosidase activities. Fold activations by Tax are shown. ** 0.01 ≦ *p* < 0.05, *** *p* < 0.01.

To explore the molecular mechanism of Tax-mediated induction of *Carm1* gene expression, we examined whether Tax activated the CARM1 promoter. For this purpose, we cloned the upstream region (−1564 to +143) of the *Carm1* gene into a luciferase reporter plasmid and examined the effect of Tax on CARM1 promoter activity in Kit 225 and Jurkat cells. Unexpectedly, Tax did not activate this upstream CARM1 promoter construct at all in Kit 225 or Jurkat cells (Figure 3A), suggesting that Tax activates the *Carm1* gene through other region(s). A search for Tax-responsive elements in the *Carm1* gene identified multiple NF-κB-like binding sequences in the first intron (Figure 3B). We thus examined whether this region in the first intron (CARM1DS) is required for Tax-mediated activation, using CARM1DS cloned upstream of an SV40 core promoter. As expected, Tax activated CARM1DS in Kit 225 cells (Figure 3C). We thus examined whether Tax activation of CARM1DS is mediated by the classical NF-κB pathway, using shRNA-mediated knockdown of the p65 (RelA) component of NF-κB, complemented by rescue by expression of an shRNA resistant p65. We also knocked down p100, the precursor of p52, the heterodimeric partner of RelB, which is activated through the nonclassical NF-κB pathway. Knockdown effects of shRNA against p65 and p100 have been confirmed in a previous report [18]. Knockdown of p65 reduced Tax-mediated activation of CARM1DS in both Kit 225 and Jurkat cells, while expression of shRNA-resistant p65 reversed these effects (Figure 3D). Similarly, shRNA against two different target sequences of p100 also reduced Tax activation of CARM1DS in both cell types (Figure 3E). These results suggest that activation of CARM1DS, and consequently the *Carm1* gene, by Tax, is mediated, at least in part, through the NF-κB pathway.

## 4. Discussion

**Figure 4:**
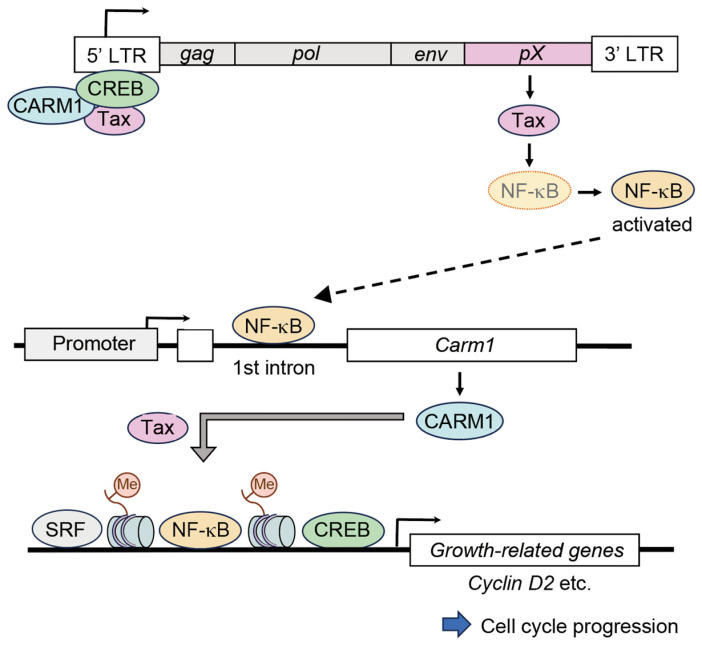
A model of the roles of the *Carm1* gene in Tax-mediated target-gene expression and cell cycle progression. Induction of CARM1 by Tax facilitates Tax-mediated target-gene expression and cell cycle progression. CARM1 and Tax form part of a transcription complex binding to the HTLV-1 LTR, leading to increased Tax transcription. Tax expression mediates activation of NF-κB, which binds to Tax response elements in the intron 1 of the *Carm1* gene. By methylating R17 of histone H3, CARM1 functions as a co-activator for NF-κB, promoting transcription of growth-related genes and cell cycle progression.

In this study, we have identified the *Carm1* gene as a novel target for Tax-mediated trans-activation and cell cycle promotion. Adenovirus-mediated introduction of Tax induced *Carm1* gene expression not only in human T-cell lines (Kit 225 and Jurkat) but also in normal PHA-stimulated PBLs. The introduction of Tax into Kit 225 cells enhanced binding of CARM1 to the Tax target gene *IL-2R*α and methylation of histone H3, as shown by the ChIP assay. ShRNA-mediated downregulation of CARM1 reduced Tax-mediated activation of the Tax target *IL-2R*α and *Cyclin D2* genes, decreased activation of E2F and suppressed cell cycle progression. Although Tax did not activate the 5′ flanking region of the *Carm1* gene, Tax response elements were identified in the first intron of the *Carm1* gene containing NF-κB binding sites. Moreover, knockdown of RelA or RelB subunits reduced Tax activation of CARM1 expression. These results indicate that CARM1 (induced by Tax through the NF-κB pathway) enhances Tax-mediated target-gene expression and the consequent cell cycle progression (Figure 4).

Trans-activation of growth-promoting genes by Tax is thought to be crucial for stimulation of proliferation of HTLV-1 infected T-cells. Tax directly activates the *cyclin D2* and *cdk6* genes through the NF-κB pathway, and induction of *cyclin D2* and *cdk6* genes is essential for Tax-mediated promotion of cell cycle progression in IL-2-starved Kit 225 cells [16,17,18]. CARM1 facilitates gene expression by methylating histones, generating a positive mark for epigenetic regulation. Expression of Tax increased methylation of HisH3R17 on the target gene *IL-2R*α with concomitant induction of CARM1, and knockdown of CARM1 reduced Tax-mediated induction of *IL-2R*α and *cyclin D2* gene expression. Knockdown of CARM1 expression, by shRNA, also reduced *cyclin D2* gene expression and decreased activation of E2F and cell cycle progression, induced by Tax. These results suggest that induction of CARM1 by Tax facilitates Tax-mediated target-gene expression and the consequent cell cycle progression, highlighting the *Carm1* gene as a crucial target of Tax, in Tax-mediated promotion of cell proliferation.

CARM1 is reported to enhance Tax-mediated activation of the HTLV-1 LTR [20]. Together with our finding that Tax induces expression of CARM1, these observations suggest a positive feedback-loop mechanism, regulating the expression of HTLV-1 viral genes, including *Tax*, which may further enhance the expression of cellular growth-promoting genes. This also underscores the importance of Tax-mediated activation of the *Carm1* gene. To the best of our knowledge, the *Carm1* gene is the first epigenetic regulator to be identified as a target of Tax-dependent trans-activation.

Activation of the *IL-2R*α and *cyclin D2* genes by Tax is primarily mediated through the NF-κB pathway. Tax-induced enhancement of the binding of CARM1 to the *IL-2R*α gene and methylation of *IL-2R*α-associated histone H3 suggest that CARM1 may function as coactivator for NF-κB. CARM1 is reported to function as a coactivator for a variety of transcription factors to facilitate the growth of cancer cells [19]. For example, CARM1 is a positive regulator of estrogen receptor alpha (ERα) and is required for the estrogen-induced expression of E2F1 in the MCF7 breast cancer cell line [52]. In colorectal cancers, CARM1 interacts with β-catenin to positively regulate target-gene expression, accompanied by methylation of R17 of histone H3, and anchorage-independent cell growth [53]. In addition, CARM1 is reported to function as a transcriptional coactivator for E2F to activate the *cyclin E1* gene [54]. These observations suggest the possibility that CARM1, induced by Tax, may also function as a coactivator for these transcription factors and contribute to the promotion of cell proliferation of HTLV-1 infected cells.

CARM1 is often over-expressed in many types of cancers, and functions as a coactivator for transcription factors that drive the transformed phenotype [19]. Targeting these driver transcription factors themselves may be difficult, but their activities could be suppressed by removing CARM1 coactivator activity. CARM1 (PRMT4) is ubiquitously expressed, and its knockout does not adversely impact embryonic development (compared to the knockout of PRMT1 or PRMT5, which is embryonic lethal), suggesting that therapeutic targeting of CARM1 may be feasible and well tolerated [19]. Tax-mediated activation of growth-related genes and promotion of cell proliferation is mainly mediated through the NF-κB pathway [1,8,9]. Our finding that the HTLV-1 oncoprotein Tax induces expression of CARM1, which subsequently augments Tax-mediated trans-activation of growth-promoting genes, suggests that CARM1 may represent a new target molecule for treatment of adult T-cell leukemia (ATL).

## Data Availability

The original contributions presented in the study are included in the article and Supplementary Materials, further inquiries can be directed to the corresponding author.

## Supplementary Materials

The following supporting information can be downloaded at: https://www.mdpi.com/article/10.3390/genes15060698/s1, Table S1: DNA microarray data 1, Table S2: DNA microarray data 2, Table S3: DNA microarray data 3.

## References

[1] Yoshida M. (2005). Discovery of HTLV-1, the first human retrovirus, its unique regulatory mechanisms, and insights into pathogenesis. Oncogene.

[2] Bangham C.R., Araujo A., Yamano Y., Taylor G.P. (2015). HTLV-1-associated myelopathy/tropical spastic paraparesis. Nat. Rev. Dis. Primers.

[3] Martin F., Taylor G.P., Jacobson S. (2014). Inflammatory manifestations of HTLV-1 and their therapeutic options. Expert Rev. Clin. Immunol..

[4] Matsuoka M., Jeang K.T. (2007). Human T-cell leukaemia virus type 1 (HTLV-1) infectivity and cellular transformation. Nat. Rev. Cancer.

[5] Forlani G., Shallak M., Accolla R.S., Romanelli M.G. (2021). HTLV-1 Infection and Pathogenesis: New Insights from Cellular and Animal Models. Int. J. Mol. Sci..

[6] Tan B.J.Y., Sugata K., Ono M., Satou Y. (2022). HTLV-1 persistence and leukemogenesis: A game of hide-and-seek with the host immune system. Front. Immunol..

[7] Giam C.Z., Semmes O.J. (2016). HTLV-1 Infection and Adult T-Cell Leukemia/Lymphoma-A Tale of Two Proteins: Tax and HBZ. Viruses.

[8] Fochi S., Mutascio S., Bertazzoni U., Zipeto D., Romanelli M.G. (2018). HTLV Deregulation of the NF-kappaB Pathway: An Update on Tax and Antisense Proteins Role. Front. Microbiol..

[9] Harhaj E.W., Giam C.Z. (2018). NF-kappaB signaling mechanisms in HTLV-1-induced adult T-cell leukemia/lymphoma. FEBS J..

[10] Zhi H., Yang L., Kuo Y.L., Ho Y.K., Shih H.M., Giam C.Z. (2011). NF-kappaB hyper-activation by HTLV-1 tax induces cellular senescence, but can be alleviated by the viral anti-sense protein HBZ. PLoS Pathog..

[11] Ho Y.K., Zhi H., DeBiaso D., Philip S., Shih H.M., Giam C.Z. (2012). HTLV-1 tax-induced rapid senescence is driven by the transcriptional activity of NF-kappaB and depends on chronically activated IKKalpha and p65/RelA. J. Virol..

[12] Matsuoka M., Mesnard J.M. (2020). HTLV-1 bZIP factor: The key viral gene for pathogenesis. Retrovirology.

[13] Giam C.Z. (2021). HTLV-1 Replication and Adult T Cell Leukemia Development. Recent Results Cancer Res..

[14] Bellon M., Nicot C. (2024). HTLV-1 Tax Tug-of-War: Cellular Senescence and Death or Cellular Transformation. Pathogens.

[15] Ohtani K., Iwanaga R., Arai M., Huang Y., Matsumura Y., Nakamura M. (2000). Cell type-specific E2F activation and cell cycle progression induced by the oncogene product Tax of human T-cell leukemia virus type I. J. Biol. Chem..

[16] Iwanaga R., Ohtani K., Hayashi T., Nakamura M. (2001). Molecular mechanism of cell cycle progression induced by the oncogene product Tax of human T-cell leukemia virus type I. Oncogene.

[17] Huang Y., Ohtani K., Iwanaga R., Matsumura Y., Nakamura M. (2001). Direct trans-activation of the human cyclin D2 gene by the oncogene product Tax of human T-cell leukemia virus type I. Oncogene.

[18] Iwanaga R., Ozono E., Fujisawa J., Ikeda M.A., Okamura N., Huang Y., Ohtani K. (2008). Activation of the cyclin D2 and cdk6 genes through NF-kappaB is critical for cell-cycle progression induced by HTLV-I Tax. Oncogene.

[19] Santos M., Hwang J.W., Bedford M.T. (2023). CARM1 arginine methyltransferase as a therapeutic target for cancer. J. Biol. Chem..

[20] Jeong S.J., Lu H., Cho W.K., Park H.U., Pise-Masison C., Brady J.N. (2006). Coactivator-associated arginine methyltransferase 1 enhances transcriptional activity of the human T-cell lymphotropic virus type 1 long terminal repeat through direct interaction with Tax. J. Virol..

[21] Hori T., Uchiyama T., Tsudo M., Umadome H., Ohno H., Fukuhara S., Kita K., Uchino H. (1987). Establishment of an interleukin 2-dependent human T cell line from a patient with T cell chronic lymphocytic leukemia who is not infected with human T cell leukemia/lymphoma virus. Blood.

[22] Kaplan J., Tilton J., Peterson W.D. (1976). Identification of T cell lymphoma tumor antigens on human T cell lines. Am. J. Hematol..

[23] Hinuma Y., Nagata K., Hanaoka M., Nakai M., Matsumoto T., Kinoshita K.I., Shirakawa S., Miyoshi I. (1981). Adult T-cell leukemia: Antigen in an ATL cell line and detection of antibodies to the antigen in human sera. Proc. Natl. Acad. Sci. USA.

[24] Kuramitsu M., Okuma K., Yamagishi M., Yamochi T., Firouzi S., Momose H., Mizukami T., Takizawa K., Araki K., Sugamura K. (2015). Identification of TL-Om1, an adult T-cell leukemia (ATL) cell line, as reference material for quantitative PCR for human T-lymphotropic virus 1. J. Clin. Microbiol..

[25] Poiesz B.J., Ruscetti F.W., Gazdar A.F., Bunn P.A., Minna J.D., Gallo R.C. (1980). Detection and isolation of type C retrovirus particles from fresh and cultured lymphocytes of a patient with cutaneous T-cell lymphoma. Proc. Natl. Acad. Sci. USA.

[26] Miyoshi I., Kubonishi I., Yoshimoto S., Akagi T., Ohtsuki Y., Shiraishi Y., Nagata K., Hinuma Y. (1981). Type C virus particles in a cord T-cell line derived by co-cultivating normal human cord leukocytes and human leukaemic T cells. Nature.

[27] Harada S., Koyanagi Y., Yamamoto N. (1985). Infection of human T-lymphotropic virus type-I (HTLV-I)-bearing MT-4 cells with HTLV-III (AIDS virus): Chronological studies of early events. Virology.

[28] Nakamura Y., Moriuchi R., Nakayama D., Yamashita I., Higashiyama Y., Yamamoto T., Kusano Y., Hino S., Miyamoto T., Katamine S. (1994). Altered expression of a novel cellular gene as a consequence of integration of human T cell lymphotropic virus type 1. J. Gen. Virol..

[29] Ohtani K., Tsujimoto A., Tsukahara T., Numata N., Miura S., Sugamura K., Nakamura M. (1998). Molecular mechanisms of promoter regulation of the gp34 gene that is trans-activated by an oncoprotein Tax of human T cell leukemia virus type I. J. Biol. Chem..

[30] Johnson D.G., Schwarz J.K., Cress W.D., Nevins J.R. (1993). Expression of transcription factor E2F1 induces quiescent cells to enter S phase. Nature.

[31] Kitamura H., Ozono E., Iwanaga R., Bradford A.P., Okuno J., Shimizu E., Kurayoshi K., Kugawa K., Toh H., Ohtani K. (2015). Identification of novel target genes specifically activated by deregulated E2F in human normal fibroblasts. Genes Cells.

[32] Zane L., Sibon D., Legras C., Lachuer J., Wierinckx A., Mehlen P., Delfau-Larue M.H., Gessain A., Gout O., Pinatel C. (2010). Clonal expansion of HTLV-1 positive CD8+ cells relies on cIAP-2 but not on c-FLIP expression. Virology.

[33] Miura S., Ohtani K., Numata N., Niki M., Ohbo K., Ina Y., Gojobori T., Tanaka Y., Tozawa H., Nakamura M. (1991). Molecular cloning and characterization of a novel glycoprotein, gp34, that is specifically induced by the human T-cell leukemia virus type I transactivator p40tax. Mol. Cell. Biol..

[34] Nakamura N., Fujii M., Tsukahara T., Arai M., Ohashi T., Wakao H., Kannagi M., Yamamoto N. (1999). Human T-cell leukemia virus type 1 Tax protein induces the expression of STAT1 and STAT5 genes in T-cells. Oncogene.

[35] Fujii M., Tsuchiya H., Chuhjo T., Akizawa T., Seiki M. (1992). Interaction of HTLV-1 Tax1 with p67SRF causes the aberrant induction of cellular immediate early genes through CArG boxes. Genes Dev..

[36] Macaire H., Riquet A., Moncollin V., Biemont-Trescol M.C., Duc Dodon M., Hermine O., Debaud A.L., Mahieux R., Mesnard J.M., Pierre M. (2012). Tax protein-induced expression of antiapoptotic Bfl-1 protein contributes to survival of human T-cell leukemia virus type 1 (HTLV-1)-infected T-cells. J. Biol. Chem..

[37] Green J.E., Begley C.G., Wagner D.K., Waldmann T.A., Jay G. (1989). trans activation of granulocyte-macrophage colony-stimulating factor and the interleukin-2 receptor in transgenic mice carrying the human T-lymphotropic virus type 1 tax gene. Mol. Cell. Biol..

[38] Masutani H., Hirota K., Sasada T., Ueda-Taniguchi Y., Taniguchi Y., Sono H., Yodoi J. (1996). Transactivation of an inducible anti-oxidative stress protein, human thioredoxin by HTLV-I Tax. Immunol. Lett..

[39] Chowdhury I.H., Farhadi A., Wang X.F., Robb M.L., Birx D.L., Kim J.H. (2003). Human T-cell leukemia virus type 1 Tax activates cyclin-dependent kinase inhibitor p21/Waf1/Cip1 expression through a p53-independent mechanism: Inhibition of cdk2. Int. J. Cancer.

[40] Owen S.M., Rudolph D.L., Dezzutti C.S., Shibata N., Naik S., Caughman S.W., Lal R.B. (1997). Transcriptional activation of the intercellular adhesion molecule 1 (CD54) gene by human T lymphotropic virus types I and II Tax is mediated through a palindromic response element. AIDS Res. Hum. Retroviruses.

[41] Iwakura Y., Tosu M., Yoshida E., Saijo S., Nakayama-Yamada J., Itagaki K., Asano M., Siomi H., Hatanaka M., Takeda T. (1995). Augmentation of c-fos and c-jun expression in transgenic mice carrying the human T-cell leukemia virus type-I tax gene. Virus Genes.

[42] Lilienbaum A., Paulin D. (1993). Activation of the human vimentin gene by the Tax human T-cell leukemia virus. I. Mechanisms of regulation by the NF-kappa B transcription factor. J. Biol. Chem..

[43] Edwards D.C., Marriott S.J. (2008). Human T-cell leukemia virus type 1 Tax relieves repression of proliferating cell nuclear antigen gene expression. J. Virol..

[44] Kress A.K., Kalmer M., Rowan A.G., Grassmann R., Fleckenstein B. (2011). The tumor marker Fascin is strongly induced by the Tax oncoprotein of HTLV-1 through NF-kappaB signals. Blood.

[45] Chen X., Zachar V., Chang C., Ebbesen P., Liu X. (1998). Differential expression of Nur77 family members in human T-lymphotropic virus type 1-infected cells: Transactivation of the TR3/nur77 gene by Tax protein. J. Virol..

[46] Pichler K., Kattan T., Gentzsch J., Kress A.K., Taylor G.P., Bangham C.R., Grassmann R. (2008). Strong induction of 4-1BB, a growth and survival promoting costimulatory receptor, in HTLV-1-infected cultured and patients’ T cells by the viral Tax oncoprotein. Blood.

[47] Masamoto I., Yoshimitsu M., Kuroki A., Horai S., Ezinne C.C., Kozako T., Hachiman M., Kamada Y., Baba M., Arima N. (2016). Clinical significance of CD70 expression on T cells in human T-lymphotropic virus type-1 carriers and adult T cell leukemia/ lymphoma patients. Leuk. Lymphoma.

[48] Zhang J., Yamada O., Kida S., Matsushita Y., Yamaoka S., Chagan-Yasutan H., Hattori T. (2011). Identification of CD44 as a downstream target of noncanonical NF-kappaB pathway activated by human T-cell leukemia virus type 1-encoded Tax protein. Virology.

[49] Bellon M., Nicot C. (2022). Feedback Loop Regulation between Pim Kinases and Tax Keeps Human T-Cell Leukemia Virus Type 1 Viral Replication in Check. J. Virol..

[50] Mori N., Fujii M., Hinz M., Nakayama K., Yamada Y., Ikeda S., Yamasaki Y., Kashanchi F., Tanaka Y., Tomonaga M. (2002). Activation of cyclin D1 and D2 promoters by human T-cell leukemia virus type I tax protein is associated with IL-2-independent growth of T cells. Int. J. Cancer.

[51] Sawada M., Suzumura A., Yoshida M., Marunouchi T. (1990). Human T-cell leukemia virus type I trans activator induces class I major histocompatibility complex antigen expression in glial cells. J. Virol..

[52] Frietze S., Lupien M., Silver P.A., Brown M. (2008). CARM1 regulates estrogen-stimulated breast cancer growth through up-regulation of E2F1. Cancer Res..

[53] Ou C.Y., LaBonte M.J., Manegold P.C., So A.Y., Ianculescu I., Gerke D.S., Yamamoto K.R., Ladner R.D., Kahn M., Kim J.H. (2011). A coactivator role of CARM1 in the dysregulation of β-catenin activity in colorectal cancer cell growth and gene expression. Mol. Cancer Res..

[54] El Messaoudi S., Fabbrizio E., Rodriguez C., Chuchana P., Fauquier L., Cheng D., Theillet C., Vandel L., Bedford M.T., Sardet C. (2006). Coactivator-associated arginine methyltransferase 1 (CARM1) is a positive regulator of the Cyclin E1 gene. Proc. Natl. Acad. Sci. USA.

