# Supplementary material for: Trans-Activation of the Coactivator-Associated Arginine Methyltransferase 1 (Carm1) Gene by the Oncogene Product Tax of Human T-Cell Leukemia Virus Type 1
Source: Genes (Basel). 2024 May 27;15(6):698. doi: 10.3390/genes15060698 (PMC11202806; doi:10.3390/genes15060698)
Supplement: Supplementary file 1 [file genes-15-00698-s001.zip › Supplementary Table S1]

Human UniGene 1 Results

## Human UniGene 1 Results

Experiment Results Generated by GEMTools 2.5  
Client: Genome\_Systems  
Export Date: Oct 12, 2001  


---

|  |  |
| --- | --- |
| GEM | 022JC38J |
| Balance Coefficient | 1.19 |
| Minimum S/B | 2.5 |
| Minimum Area | 40% |
| Probe 1 | 123YA1BU |
| P1 Description | d17/5 |
| Probe 2 | 1235A1BV |
| P2 Description | wt |

---

Report sorted by **Balanced Diff Expr** in **Ascending** order  
Ranks from **1** to **100**

### Order LifeArray clones

Legend

| Rank | Location | Diff Expr | Balanced Diff Expr | P1 Signal | P1 S/B | P1 Area % | P2 Balanced Signal | P2 Signal | P2 S/B | P2 Area % | Plate Row | Plate Col | Plate ID | Gene Name | PCR Status | GenBank Id | Clone Id (Sequence) | Vector |
| --- | --- | --- | --- | --- | --- | --- | --- | --- | --- | --- | --- | --- | --- | --- | --- | --- | --- | --- |
| 1 | 2525 | -12.7 | **-15.1** | 199 | 2.6 | 57 | 3011 | 2530 | 41.2 | 57 | A | 9 | 021MAKNL | Control: Ratio 1:25 (Cy3:Cy5) |  |  |  |  |
| 2 | 10175 | -12.4 | **-14.7** | 222 | 2.9 | 55 | 3264 | 2743 | 45.2 | 55 | B | 10 | 021MAKNL | Control: Ratio 1:25 (Cy3:Cy5) |  |  |  |  |
| 3 | 7625 | -11.9 | **-14.2** | 299 | 3.6 | 58 | 4242 | 3565 | 62.5 | 58 | B | 9 | 021MAKNL | Control: Ratio 1:25 (Cy3:Cy5) |  |  |  |  |
| 4 | 8052 | -11.7 | **-14.0** | 130 | 1.9 | 63**†** | 1816 | 1526 | 17.4 | 63 | F | 12 | 021HAGL1 | regulator of G-protein signalling 1 | Passed | S59049 Entrez UniGene | 3120390 | pINCY |
| 5 | 5075 | -11.7 | **-13.9** | 172 | 2.4 | 51**†** | 2395 | 2013 | 29.8 | 51 | A | 10 | 021MAKNL | Control: Ratio 1:25 (Cy3:Cy5) |  |  |  |  |
| 6 | 8433 | -11.3 | **-13.5** | 188 | 2.5 | 94 | 2534 | 2129 | 28.3 | 94 | F | 6 | 021YAGLH | small inducible cytokine A1 (I-309, homologous to mouse Tca-3) | Passed | NM\_002981 Entrez UniGene | 8990 | pBlue |
| 7 | 10173 | -10.9 | **-13.0** | 396 | 4.4 | 58 | 5138 | 4318 | 68.5 | 58 | B | 6 | 021MAKNL | Control: Ratio 1:10 (Cy3:Cy5) |  |  |  |  |
| 8 | 2523 | -10.7 | **-12.7** | 344 | 3.5 | 87 | 4383 | 3683 | 54.4 | 87 | A | 5 | 021MAKNL | Control: Ratio 1:10 (Cy3:Cy5) |  |  |  |  |
| 9 | 7655 | -8.9 | **-10.6** | 139 | 2.0 | 57**†** | 1478 | 1242 | 17.1 | 57 | B | 10 | 0216AKON | Internal\_Control\_I |  |  |  |  |
| 10 | 7623 | -8.6 | **-10.3** | 271 | 3.2 | 58 | 2786 | 2341 | 35.9 | 58 | B | 5 | 021MAKNL | Control: Ratio 1:10 (Cy3:Cy5) |  |  |  |  |
| 11 | 5 | -7.7 | **-9.2** | 165 | 1.9 | 68**†** | 1517 | 1275 | 16.9 | 68 | A | 9 | 0216AKON | Internal\_Control\_I |  |  |  |  |
| 12 | 5368 | -7.6 | **-9.1** | 307 | 3.4 | 66 | 2794 | 2348 | 30.4 | 66 | B | 7 | 021PAGKW | BCL2-like 1 | Passed | BE780536 Entrez UniGene | 1855683 | pINCY |
| 13 | 5731 | -7.5 | **-8.9** | 788 | 8.2 | 68 | 7038 | 5914 | 101.2 | 68 | D | 1 | 021SAGLB | proteoglycan 1, secretory granule | Passed | NM\_002727 Entrez UniGene | 8995 | pBlue |
| 14 | 5105 | -7.3 | **-8.7** | 189 | 2.2 | 53**†** | 1651 | 1387 | 18.1 | 53 | B | 9 | 0216AKON | Internal\_Control\_I |  |  |  |  |
| 15 | 5073 | -5.7 | **-6.8** | 182 | 2.5 | 75 | 1236 | 1039 | 17.0 | 75 | A | 6 | 021MAKNL | Control: Ratio 1:10 (Cy3:Cy5) |  |  |  |  |
| 16 | 5103 | -5.5 | **-6.6** | 288 | 2.8 | 55 | 1902 | 1598 | 22.0 | 55 | B | 5 | 0216AKON | Internal\_Control\_G |  |  |  |  |
| 17 | 7653 | -5.0 | **-6.0** | 267 | 2.8 | 44 | 1596 | 1341 | 18.0 | 44 | B | 6 | 0216AKON | Internal\_Control\_G |  |  |  |  |
| 18 | 4242 | -4.9 | **-5.9** | 519 | 4.5 | 100 | 3045 | 2559 | 31.5 | 100 | C | 12 | 021IAGMJ | Epstein-Barr virus induced gene 3 | Passed | BG620398 Entrez UniGene | 3745468 | pINCY |
| 19 | 3717 | -4.9 | **-5.8** | 164 | 2.3 | 100**†** | 957 | 804 | 12.0 | 100 | E | 6 | 0212AGLX | baculoviral IAP repeat-containing 3 | Passed | AI581499 Entrez UniGene | 1603857 | pINCY |
| 20 | 1502 | -4.8 | **-5.7** | 547 | 4.5 | 100 | 3099 | 2604 | 32.4 | 100 | E | 3 | 021YAGMB | dual specificity phosphatase 2 | Passed | BC007771 Entrez UniGene | 518826 | pSport1 |
| 21 | 2555 | -4.4 | **-5.3** | 144 | 1.8 | 48**†** | 760 | 639 | 8.8 | 48 | A | 10 | 0216AKON | Internal\_Control\_I |  |  |  |  |
| 22 | 8205 | -4.5 | **-5.3** | 97 | 1.7 | 68**†** | 515 | 433 | 6.3 | 68 | B | 6 | 021UAGL8 | caveolin 1, caveolae protein, 22kD | Passed | BG541572 Entrez UniGene | 4271973 | pINCY |
| 23 | 8201 | -4.3 | **-5.1** | 39 | 1.3 | 40**†** | 200 | 168 | 3.0 | 40 | H | 10 | 021NAGL7 | deleted in azoospermia-like | Passed | NM\_001351 Entrez UniGene | 4919920 | pINCY |
| 24 | 4636 | -3.9 | **-4.7** | 604 | 6.5 | 61 | 2836 | 2383 | 38.8 | 61 | G | 8 | 021MAGMZ | singed (Drosophila)-like (sea urchin fascin homolog like) | Passed | BG774457 Entrez UniGene | 1656271 | pINCY |
| 25 | 3 | -3.6 | **-4.3** | 233 | 2.2 | 62**†** | 995 | 836 | 11.2 | 62 | A | 5 | 0216AKON | Internal\_Control\_G |  |  |  |  |
| 26 | 4420 | -3.6 | **-4.3** | 130 | 2.0 | 81**†** | 553 | 465 | 7.3 | 81 | G | 8 | 021VAGMQ | tumor necrosis factor (ligand) superfamily, member 4 (tax-transcriptionally activated glycoprotein 1, 34kD) | Multiple Bands | BE349175 Entrez UniGene | 1512102 | pINCY |
| 27 | 4457 | -3.3 | **-4.0** | 174 | 2.4 | 74**†** | 688 | 578 | 9.4 | 74 | C | 10 | 0219AGMS | signal transducer and activator of transcription 5A | Passed | AI582321 Entrez UniGene | 606641 | pSport1 |
| 28 | 4998 | -3.2 | **-3.8** | 177 | 2.5 | 88 | 664 | 558 | 10.3 | 88 | G | 12 | 021PAGNE | early growth response 2 (Krox-20 (Drosophila) homolog) | Passed | BG743293 Entrez UniGene | 3603037 | pINCY |
| 29 | 538 | -3.1 | **-3.7** | 712 | 5.7 | 58 | 2600 | 2185 | 24.8 | 58 | C | 7 | 021NAGL7 | glycoprotein, synaptic 2 | Passed | BG282184 Entrez UniGene | 3721920 | pINCY |
| 30 | 1476 | -3.0 | **-3.6** | 1147 | 10.8 | 100 | 4125 | 3466 | 56.0 | 100 | C | 11 | 021RAGMA | baculoviral IAP repeat-containing 3 | Passed | U37546 Entrez UniGene | 1513214 | pINCY |
| 31 | 1539 | -2.8 | **-3.3** | 116 | 1.7 | 63**†** | 382 | 321 | 4.5 | 63 | A | 5 | 021CAGMD | aminolevulinate, delta-, synthase 1 | Passed | BF969214 Entrez UniGene | 943569 | pSport1 |
| 32 | 10171 | -2.8 | **-3.3** | 149 | 2.3 | 81**†** | 494 | 415 | 7.2 | 81 | B | 2 | 021MAKNL | Control: Ratio 1:3 (Cy3:Cy5) |  |  |  |  |
| 33 | 7621 | -2.8 | **-3.3** | 161 | 2.3 | 78**†** | 533 | 448 | 7.9 | 78 | B | 1 | 021MAKNL | Control: Ratio 1:3 (Cy3:Cy5) |  |  |  |  |
| 34 | 2553 | -2.8 | **-3.3** | 321 | 2.9 | 44 | 1066 | 896 | 12.5 | 44 | A | 6 | 0216AKON | Internal\_Control\_G |  |  |  |  |
| 35 | 8990 | -2.8 | **-3.3** | 102 | 1.9 | 96**†** | 334 | 281 | 5.6 | 96 | H | 4 | 0218AGM4 | potassium voltage-gated channel, KQT-like subfamily, member 2 | Passed | AF110020 Entrez UniGene | 617878 | pSport1 |
| 36 | 187 | -2.6 | **-3.2** | 693 | 6.2 | 65 | 2184 | 1835 | 32.6 | 65 | G | 1 | 021XAGKS | DnaJ (Hsp40) homolog, subfamily A, member 1 | Passed | NM\_001539 Entrez UniGene | 1926883 | pSport1 |
| 37 | 8846 | -2.7 | **-3.2** | 291 | 3.4 | 65 | 922 | 775 | 12.4 | 65 | H | 4 | 0219AGLY | ninjurin 1 | Passed | BG530047 Entrez UniGene | 2927362 | pINCY |
| 38 | 5071 | -2.5 | **-3.0** | 163 | 2.3 | 73**†** | 486 | 408 | 6.7 | 73 | A | 2 | 021MAKNL | Control: Ratio 1:3 (Cy3:Cy5) |  |  |  |  |
| 39 | 5101 | -2.4 | **-2.9** | 389 | 3.0 | 49 | 1122 | 943 | 12.1 | 49 | B | 1 | 0216AKON | Internal\_Control\_E |  |  |  |  |
| 40 | 1653 | -2.4 | **-2.9** | 218 | 2.4 | 96**†** | 634 | 533 | 8.6 | 96 | G | 5 | 0214AGMH | BCL2-related protein A1 | Passed | BF677029 Entrez UniGene | 2555673 | pINCY |
| 41 | 5205 | -2.4 | **-2.8** | 3504 | 28.4 | 51 | 9981 | 8387 | 111.4 | 51 | D | 5 | 021CAGKP | colony stimulating factor 2 (granulocyte-macrophage) | Passed | BE669962 Entrez UniGene | 3297733 | pINCY |
| 42 | 7982 | -2.4 | **-2.8** | 394 | 4.2 | 67 | 1103 | 927 | 15.5 | 67 | H | 4 | 0213AGKY | regulator of G-protein signalling 1 | Passed | S59049 Entrez UniGene | 1728022 | pINCY |
| 43 | 8229 | -2.3 | **-2.8** | 129 | 2.2 | 87**†** | 361 | 303 | 5.6 | 87 | B | 6 | 0211AGL9 | syndecan 4 (amphiglycan, ryudocan) | Passed | AI582184 Entrez UniGene | 5183574 | pINCY |
| 44 | 2521 | -2.3 | **-2.8** | 188 | 2.3 | 74**†** | 518 | 435 | 6.5 | 74 | A | 1 | 021MAKNL | Control: Ratio 1:3 (Cy3:Cy5) |  |  |  |  |
| 45 | 5156 | -2.3 | **-2.8** | 344 | 3.5 | 68 | 957 | 804 | 10.8 | 68 | D | 3 | 021YAGKN | BTG family, member 2 | No Amplification | NM\_006763 Entrez UniGene | 1598617 | pINCY |
| 46 | 7651 | -2.4 | **-2.8** | 272 | 2.7 | 55 | 769 | 646 | 8.8 | 55 | B | 2 | 0216AKON | Internal\_Control\_E |  |  |  |  |
| 47 | 9527 | -2.4 | **-2.8** | 335 | 4.0 | 91 | 950 | 798 | 14.3 | 91 | B | 10 | 0212AGMR | tumor necrosis factor receptor superfamily, member 6 | Passed | AL542093 Entrez UniGene | 2205246 | pINCY |
| 48 | 2551 | -2.3 | **-2.8** | 339 | 2.8 | 55 | 941 | 791 | 10.5 | 55 | A | 2 | 0216AKON | Internal\_Control\_E |  |  |  |  |
| 49 | 6903 | -2.4 | **-2.8** | 1476 | 12.4 | 91 | 4139 | 3478 | 42.4 | 91 | B | 5 | 021HAGMO | thioredoxin | Passed | AV763087 Entrez UniGene | 2606240 | pINCY |
| 50 | 7333 | -2.4 | **-2.8** | 719 | 6.8 | 100 | 2016 | 1694 | 26.3 | 100 | B | 1 | 021SAGN6 | suppression of tumorigenicity 14 (colon carcinoma, matriptase, epithin) | Passed | AL548113 Entrez UniGene | 478960 | pSport1 |
| 51 | 2156 | -2.3 | **-2.8** | 508 | 4.4 | 100 | 1398 | 1175 | 16.7 | 100 | G | 3 | 0210AGN2 | baculoviral IAP repeat-containing 2 | Passed | U37547 Entrez UniGene | 1810777 | pINCY |
| 52 | 33 | -2.3 | **-2.7** | 553 | 4.4 | 70 | 1517 | 1275 | 17.1 | 70 | C | 5 | 021RAGKM | neutrophil cytosolic factor 2 (65kD, chronic granulomatous disease, autosomal 2) | Passed | NM\_000433 Entrez UniGene | 1556718 | pINCY |
| 53 | 9342 | -2.2 | **-2.6** | 728 | 7.3 | 67 | 1885 | 1584 | 26.1 | 67 | D | 12 | 021IAGMJ | peptidyl prolyl isomerase H (cyclophilin H) | Passed | BF794821 Entrez UniGene | 3667096 | pINCY |
| 54 | 5140 | -2.2 | **-2.6** | 456 | 3.8 | 62 | 1196 | 1005 | 13.0 | 62 | F | 7 | 021RAGKM | ring finger protein 1 | Passed | AL576514 Entrez UniGene | 1879727 | pINCY |
| 55 | 1581 | -2.2 | **-2.6** | 835 | 6.8 | 75 | 2198 | 1847 | 26.3 | 75 | G | 5 | 021JAGME | coactivator-associated arginine methyltransferase-1 | Passed | BG830500 Entrez UniGene | 2807446 | pINCY |
| 56 | 3525 | -2.2 | **-2.6** | 400 | 4.1 | 100 | 1029 | 865 | 12.7 | 100 | E | 6 | 021IAGLP | HtrA-like serine protease | Passed | AL577683 Entrez UniGene | 1718257 | pINCY |
| 57 | 9661 | -2.1 | **-2.6** | 356 | 3.8 | 89 | 908 | 763 | 11.6 | 89 | H | 2 | 0211AGMW | cyclin-dependent kinase inhibitor 1A (p21, Cip1) | Passed | L26165 Entrez UniGene | 1804548 | pINCY |
| 58 | 8307 | -2.1 | **-2.5** | 244 | 2.9 | 68 | 599 | 503 | 8.2 | 68 | D | 6 | 021ZAGLC | squalene epoxidase | No Amplification | AF098865 Entrez UniGene | 859645 | pSport1 |
| 59 | 2275 | -2.1 | **-2.5** | 91 | 1.5 | 71**†** | 223 | 187 | 2.8 | 71 | G | 1 | 021ZAGN7 | JAK binding protein | Passed | BE514365 Entrez UniGene | 445246 | pBlue |
| 60 | 2138 | -2.1 | **-2.5** | 299 | 3.4 | 73 | 759 | 638 | 10.7 | 73 | A | 3 | 0210AGN2 | mevalonate (diphospho) decarboxylase | Passed | BG324529 Entrez UniGene | 1711364 | pINCY |
| 61 | 67 | -2.1 | **-2.5** | 3360 | 25.9 | 60 | 8491 | 7135 | 114.3 | 60 | G | 1 | 021YAGKN | lymphocyte cytosolic protein 1 (L-plastin) | Passed | BC007673 Entrez UniGene | 1363074 | pINCY |
| 62 | 9470 | -2.1 | **-2.5** | 407 | 4.5 | 100 | 1015 | 853 | 14.8 | 100 | H | 4 | 021HAGMO | tyrosine kinase 2 | Passed | NM\_003331 Entrez UniGene | 1831805 | pINCY |
| 63 | 3611 | -2.1 | **-2.5** | 616 | 6.5 | 66 | 1533 | 1288 | 20.2 | 66 | A | 10 | 021AAGLT | G-rich RNA sequence binding factor 1 | Passed | BF034561 Entrez UniGene | 1931925 | pINCY |
| 64 | 3735 | -2.1 | **-2.5** | 789 | 7.8 | 100 | 1974 | 1659 | 26.5 | 100 | C | 6 | 0219AGLY | intercellular adhesion molecule 1 (CD54), human rhinovirus receptor | Passed | M24283 Entrez UniGene | 1556061 | pINCY |
| 65 | 7320 | -2.1 | **-2.5** | 162 | 2.2 | 91**†** | 407 | 342 | 5.5 | 91 | D | 11 | 021LAGN5 | GTP-binding protein overexpressed in skeletal muscle | Passed | AW297828 Entrez UniGene | 450618 | pBlue |
| 66 | 3294 | -2.0 | **-2.4** | 585 | 5.8 | 100 | 1422 | 1195 | 18.1 | 100 | G | 12 | 021KAGLF | protein geranylgeranyltransferase type I, beta subunit | Passed | AA481712 Entrez UniGene | 815861 | pSport1 |
| 67 | 4916 | -2.0 | **-2.4** | 797 | 8.2 | 65 | 1879 | 1579 | 27.3 | 65 | E | 4 | 0214AGNB | translocase of inner mitochondrial membrane 17 (yeast) homolog A | Passed | BG506029 Entrez UniGene | 2458933 | pINCY |
| 68 | 6204 | -2.0 | **-2.4** | 573 | 5.2 | 100 | 1348 | 1133 | 16.3 | 100 | H | 11 | 021HAGLU | small inducible cytokine subfamily A (Cys-Cys), member 20 | Passed | NM\_004591 Entrez UniGene | 2220923 | pINCY |
| 69 | 9619 | -2.0 | **-2.4** | 274 | 3.4 | 86 | 662 | 556 | 9.3 | 86 | B | 2 | 021UAGMV | tryptophanyl-tRNA synthetase | Passed | BF795451 Entrez UniGene | 1846209 | pSport1 |
| 70 | 2295 | -2.0 | **-2.4** | 193 | 2.7 | 82 | 468 | 393 | 7.4 | 82 | E | 5 | 0216AGN8 | prostaglandin E receptor 4 (subtype EP4) | Passed | NM\_000958 Entrez UniGene | 1631793 | pINCY |
| 71 | 6097 | -2.0 | **-2.4** | 140 | 2.0 | 65**†** | 330 | 277 | 4.5 | 65 | F | 1 | 021PAGLQ | hypothetical protein, clone 2746033 | Passed | BE745844 Entrez UniGene | 1923769 | pSport1 |
| 72 | 9828 | -2.0 | **-2.4** | 97 | 1.9 | 91**†** | 234 | 197 | 4.3 | 91 | F | 12 | 0217AGN3 | vesicle-associated membrane protein 5 (myobrevin) | Passed | AF151025 Entrez UniGene | 122826 | pBlue |
| 73 | 2595 | -1.9 | **-2.3** | 576 | 4.5 | 65 | 1308 | 1099 | 14.4 | 65 | G | 6 | 021RAGKM | integrin, beta 2 (antigen CD18 (p95), lymphocyte function-associated antigen 1; macrophage antigen 1 (mac-1) beta subunit) | Passed | BC005861 Entrez UniGene | 1871113 | pINCY |
| 74 | 10091 | -1.9 | **-2.3** | 262 | 2.9 | 65 | 590 | 496 | 7.7 | 65 | F | 10 | 021PAGNE | ELL-RELATED RNA POLYMERASE II, ELONGATION FACTOR | Passed | NM\_012081 Entrez UniGene | 1281473 | pINCY |
| 75 | 5187 | -1.9 | **-2.3** | 645 | 4.9 | 60 | 1454 | 1222 | 13.3 | 60 | F | 5 | 0215AGKO | flap structure-specific endonuclease 1 | Passed | AU142907 Entrez UniGene | 2050085 | pINCY |
| 76 | 2150 | -1.9 | **-2.3** | 971 | 8.1 | 61 | 2212 | 1859 | 25.8 | 61 | E | 3 | 0210AGN2 | heat shock 70kD protein 5 (glucose-regulated protein, 78kD) | Passed | AI878886 Entrez UniGene | 2884613 | pINCY |
| 77 | 4561 | -2.0 | **-2.3** | 227 | 3.0 | 75 | 532 | 447 | 8.5 | 75 | G | 2 | 0211AGMW | chitobiase, di-N-acetyl- | Passed | AA688097 Entrez UniGene | 2879077 | pINCY |
| 78 | 1420 | -1.9 | **-2.2** | 449 | 4.0 | 100 | 1007 | 846 | 11.6 | 100 | A | 7 | 0210AGM8 | SH3-domain binding protein 5 (BTK-associated) | Passed | BG030766 Entrez UniGene | 2170638 | pINCY |
| 79 | 7696 | -1.9 | **-2.2** | 213 | 2.4 | 68**†** | 474 | 398 | 6.4 | 68 | H | 8 | 021RAGKM | phorbol-12-myristate-13-acetate-induced protein 1 | Passed | BG776688 Entrez UniGene | 1931117 | pINCY |
| 80 | 2654 | -1.9 | **-2.2** | 729 | 6.2 | 75 | 1627 | 1367 | 20.8 | 75 | C | 4 | 021CAGKP | vacuolar protein sorting 41 (yeast homolog) | No Amplification | NM\_014396 Entrez UniGene | 2910949 | pINCY |
| 81 | 2975 | -1.8 | **-2.1** | 114 | 1.9 | 93**†** | 244 | 205 | 3.8 | 93 | E | 10 | 021OAGL2 | syndecan 4 (amphiglycan, ryudocan) | No Amplification | NM\_002999 Entrez UniGene | 3214670 | pINCY |
| 82 | 1990 | -1.7 | **-2.1** | 115 | 1.9 | 91**†** | 239 | 201 | 3.8 | 91 | G | 7 | 021UAGMV | protein kinase C, delta | No Amplification | L07861 Entrez UniGene | 613603 | pSport1 |
| 83 | 4934 | -1.7 | **-2.1** | 138 | 2.1 | 66**†** | 284 | 239 | 4.1 | 66 | C | 4 | 021BAGNC | cyclin-dependent kinase 7 (homolog of Xenopus MO15 cdk-activating kinase) | Passed | BE887969 Entrez UniGene | 1558108 | pINCY |
| 84 | 687 | -1.7 | **-2.1** | 249 | 2.6 | 75 | 515 | 433 | 6.6 | 75 | E | 5 | 0216AGLD | UDP-N-acteylglucosamine pyrophosphorylase 1 | Passed | AL520091 Entrez UniGene | 1997038 | pSport1 |
| 85 | 5039 | -1.8 | **-2.1** | 251 | 3.1 | 73 | 525 | 441 | 7.4 | 73 | E | 10 | 0213AGNG | reticulocalbin 1, EF-hand calcium binding domain | Passed | NM\_002901 Entrez UniGene | 2057296 | pSport1 |
| 86 | 182 | -1.8 | **-2.1** | 478 | 4.4 | 65 | 1022 | 859 | 13.6 | 65 | E | 3 | 021XAGKS | v-jun avian sarcoma virus 17 oncogene homolog | Passed | AI078377 Entrez UniGene | 1969563 | pSport1 |
| 87 | 9135 | -1.7 | **-2.1** | 983 | 9.5 | 100 | 2022 | 1699 | 26.7 | 100 | H | 6 | 021RAGMA | serine (or cysteine) proteinase inhibitor, clade E (nexin, plasminogen activator inhibitor type 1), member 1 | Passed | BE812315 Entrez UniGene | 1445767 | pINCY |
| 88 | 3217 | -1.8 | **-2.1** | 1599 | 13.2 | 100 | 3401 | 2858 | 38.6 | 100 | G | 2 | 021ZAGLC | synaptogyrin 2 | Passed | AL545227 Entrez UniGene | 983008 | pSport1 |
| 89 | 10027 | -1.7 | **-2.1** | 3229 | 26.8 | 77 | 6660 | 5597 | 81.0 | 77 | B | 2 | 021BAGNC | vimentin | Passed | AL572054 Entrez UniGene | 1522716 | pINCY |
| 90 | 7030 | -1.7 | **-2.0** | 482 | 4.9 | 100 | 953 | 801 | 13.1 | 100 | D | 7 | 021GAGMT | basement membrane-induced gene | Passed | NM\_004848 Entrez UniGene | 1840811 | pSport1 |
| 91 | 1315 | -1.7 | **-2.0** | 600 | 5.0 | 100 | 1194 | 1003 | 13.7 | 100 | G | 1 | 0211AGM3 | dual specificity phosphatase 5 | Passed | U16996 Entrez UniGene | 1734561 | pINCY |
| 92 | 7043 | -1.6 | **-2.0** | 334 | 3.4 | 67 | 656 | 551 | 8.9 | 67 | H | 9 | 021GAGMT | BTG family, member 3 | Passed | BG110736 Entrez UniGene | 637576 | pSport1 |
| 93 | 2888 | -1.7 | **-2.0** | 192 | 2.7 | 92 | 392 | 329 | 6.6 | 92 | A | 4 | 021AAGKZ | oxygen regulated protein (150kD) | Passed | AI969119 Entrez UniGene | 2398659 | pINCY |
| 94 | 8729 | -1.7 | **-2.0** | 117 | 2.1 | 96**†** | 239 | 201 | 4.4 | 96 | H | 10 | 021AAGLT | plastin 3 (T isoform) | Passed | BF683154 Entrez UniGene | 1402228 | pINCY |
| 95 | 7464 | -1.7 | **-2.0** | 1053 | 9.7 | 100 | 2078 | 1746 | 27.1 | 100 | D | 11 | 0214AGNB | proliferating cell nuclear antigen | Passed | AA523378 Entrez UniGene | 2781405 | pINCY |
| 96 | 2233 | -1.7 | **-2.0** | 459 | 4.1 | 68 | 916 | 770 | 10.6 | 68 | A | 1 | 021SAGN6 | ADP-ribosylation factor-like 3 | Passed | BI013116 Entrez UniGene | 67300 | pBlue |
| 97 | 4100 | -1.7 | **-2.0** | 818 | 6.9 | 62 | 1628 | 1368 | 19.7 | 62 | E | 4 | 021CAGMD | ferritin, heavy polypeptide 1 | Passed | BE878314 Entrez UniGene | 27775 | pBlue |
| 98 | 7377 | -1.7 | **-2.0** | 754 | 7.6 | 100 | 1516 | 1274 | 21.5 | 100 | H | 5 | 021ZAGN7 | endothelin converting enzyme 1 | Passed | NM\_001397 Entrez UniGene | 1963819 | pSport1 |
| 99 | 7018 | -1.6 | **-1.9** | 257 | 2.9 | 89 | 480 | 403 | 6.8 | 89 | H | 7 | 0219AGMS | preferentially expressed antigen in melanoma | Passed | AI017284 Entrez UniGene | 2007554 | pBlue |
| 100 | 4844 | -1.6 | **-1.9** | 762 | 6.3 | 65 | 1440 | 1210 | 16.9 | 65 | E | 4 | 0216AGN8 | cell division cycle 42 (GTP-binding protein, 25kD) | Passed | AV722422 Entrez UniGene | 2834543 | pINCY |

---

**†** Probe 1 did not meet selection criteria

---

### Order LifeArray clones

Next 100

**1**
2
3
4
5
6
7
8
9
10
11
12
13
14
15
16
17
18
19
20
21
22
23
24
25
26
27
28
29
30
31
32
33
34
35
36
37
38
39
40
41
42
43
44
45
46
47
48
49
50
51
52
53
54
55
56
57
58
59
60
61
62
63
64
65
66
67
68
69
70
71
72
73
74
75
76
77
78
79
80
81
82
83
84
85
86
87
88
89
90
91
92
93
94

Entire List in plain text (long -- 1.91 MB)

|  |  |  |
| --- | --- | --- |
| PDF image of LifeArray (long -- 2.43 MB) | LifeArray color bar: |  |

To save your LifeArray™ results on your computer, use the 'Plain Text' option to display your results, then save them on your computer with your browser's save feature. We will also provide your LifeArray results on a CD-ROM for a nominal fee. Please contact our Technical Support group if you need any assistance.

We guarantee that your LifeArray results will remain on the server for 90 days after it was first uploaded. After that, we may remove and archive your LifeArray results at our discretion. Please contact our Technical Support group if you need any archived LifeArray results restored to our server.

In order to view or print Adobe® Acrobat® PDF files, you need the Adobe Acrobat Reader. If you do not already have it installed, you can obtain it for free from the Adobe web site .

If you have questions about the documents or have difficulty downloading the Acrobat Reader, please contact us.

Download the LifeArray Frequently Asked Questions list in HTML format.

Download the Human UniGEM V Frequently Asked Questions list in HTML format.

Download the LifeArray Control Plate Document in HTML format.

Adobe and Acrobat are trademarks of Adobe Systems Incorporated.

---

### Sort Again:

|  |  |
| --- | --- |
| **Username:** |  |
| **Password:** |  |
| **Sort Order:** | Ascending Descending |
| **Sort By:** | Location Diff Expr Balanced Diff Expr P1 Signal P1 S/B P2 Balanced Signal P2 Signal P2 S/B Plate ID/Row/Col Gene Name |
| **Plate ID:** |  |
| **Gene Name:** |  |

  

---

LifeArray Products  
Incyte Genomics Reagents Home
