# Supplementary material for: Trans-Activation of the Coactivator-Associated Arginine Methyltransferase 1 (Carm1) Gene by the Oncogene Product Tax of Human T-Cell Leukemia Virus Type 1
Source: Genes (Basel). 2024 May 27;15(6):698. doi: 10.3390/genes15060698 (PMC11202806; doi:10.3390/genes15060698)
Supplement: Supplementary file 1 [file genes-15-00698-s001.zip › Supplementary Table S1.pdf]

Human UniGene 1 Results

Experiment Results Generated by GEMTools 2.5
Client: Genome_Systems
Export Date: Oct 12, 2001

**GEM**	022JC38J
**Balance Coefficient**	1.19
**Minimum S/B**	2.5
**Minimum Area**	40%
**Probe 1**	123YA1BU
**P1 Description**	d17/5
**Probe 2**	1235A1BV
**P2 Description**	wt

Report sorted by **Balanced Diff Expr** in **Ascending** order
Ranks from **1** to **100**

Order LifeArray clones

Legend

Rank	Location	Diff Expr	Balanced Diff Expr	P1 Signal	P1 S/B	P1 Area %	P2 Balanced Signal	P2 Signal	P2 S/B	P2 Area %	Plate Row	Plate Col	Plate ID	Gene Name	PCR Status	GenBank Id	Clone Id (Sequence)	Vector
1	2525	-12.7	**-15.1**	199	2.6	57	3011	2530	41.2	57	A	9	021MAKNL	Control: Ratio 1:25 (Cy3:Cy5)				
2	10175	-12.4	**-14.7**	222	2.9	55	3264	2743	45.2	55	B	10	021MAKNL	Control: Ratio 1:25 (Cy3:Cy5)				
3	7625	-11.9	**-14.2**	299	3.6	58	4242	3565	62.5	58	B	9	021MAKNL	Control: Ratio 1:25 (Cy3:Cy5)				
4	8052	-11.7	**-14.0**	130	1.9	63†	1816	1526	17.4	63	F	12	021HAGL1	regulator of G-protein signalling 1	Passed	S59049 Entrez UniGene	3120390	pINCY

5	5075	-11.7	**-13.9**	172	2.4	51†	2395	2013	29.8	51	A	10	021MAKNL	Control: Ratio 1:25 (Cy3:Cy5)				
6	8433	-11.3	**-13.5**	188	2.5	94	2534	2129	28.3	94	F	6	021YAGLH	small inducible cytokine A1 (I-309, homologous to mouse Tca-3)	Passed	NM_002981 Entrez UniGene	8990	pBlue
7	10173	-10.9	**-13.0**	396	4.4	58	5138	4318	68.5	58	B	6	021MAKNL	Control: Ratio 1:10 (Cy3:Cy5)				
8	2523	-10.7	**-12.7**	344	3.5	87	4383	3683	54.4	87	A	5	021MAKNL	Control: Ratio 1:10 (Cy3:Cy5)				
9	7655	-8.9	**-10.6**	139	2.0	57†	1478	1242	17.1	57	B	10	0216AKON	Internal_Control_I				
10	7623	-8.6	**-10.3**	271	3.2	58	2786	2341	35.9	58	B	5	021MAKNL	Control: Ratio 1:10 (Cy3:Cy5)				
11	5	-7.7	**-9.2**	165	1.9	68†	1517	1275	16.9	68	A	9	0216AKON	Internal_Control_I				
12	5368	-7.6	**-9.1**	307	3.4	66	2794	2348	30.4	66	B	7	021PAGKW	BCL2-like 1	Passed	BE780536 Entrez UniGene	1855683	pINCY
13	5731	-7.5	**-8.9**	788	8.2	68	7038	5914	101.2	68	D	1	021SAGLB	proteoglycan 1, secretory granule	Passed	NM_002727 Entrez UniGene	8995	pBlue
14	5105	-7.3	**-8.7**	189	2.2	53†	1651	1387	18.1	53	B	9	0216AKON	Internal_Control_I				
15	5073	-5.7	**-6.8**	182	2.5	75	1236	1039	17.0	75	A	6	021MAKNL	Control: Ratio 1:10 (Cy3:Cy5)				
16	5103	-5.5	**-6.6**	288	2.8	55	1902	1598	22.0	55	B	5	0216AKON	Internal_Control_G				

17	7653	-5.0	**-6.0**	267	2.8	44	1596	1341	18.0	44	B	6	0216AKON	Internal_Control_G				
18	4242	-4.9	**-5.9**	519	4.5	100	3045	2559	31.5	100	C	12	021IAGMJ	Epstein-Barr virus induced gene 3	Passed	BG620398 Entrez UniGene	3745468	pINCY
19	3717	-4.9	**-5.8**	164	2.3	100†	957	804	12.0	100	E	6	0212AGLX	baculoviral IAP repeat-containing 3	Passed	AI581499 Entrez UniGene	1603857	pINCY
20	1502	-4.8	**-5.7**	547	4.5	100	3099	2604	32.4	100	E	3	021YAGMB	dual specificity phosphatase 2	Passed	BC007771 Entrez UniGene	518826	pSport1
21	2555	-4.4	**-5.3**	144	1.8	48†	760	639	8.8	48	A	10	0216AKON	Internal_Control_I				
22	8205	-4.5	**-5.3**	97	1.7	68†	515	433	6.3	68	B	6	021UAGL8	caveolin 1, caveolae protein, 22kD	Passed	BG541572 Entrez UniGene	4271973	pINCY
23	8201	-4.3	**-5.1**	39	1.3	40†	200	168	3.0	40	H	10	021NAGL7	deleted in azoospermia-like	Passed	NM_001351 Entrez UniGene	4919920	pINCY
24	4636	-3.9	**-4.7**	604	6.5	61	2836	2383	38.8	61	G	8	021MAGMZ	singed (Drosophila)-like (sea urchin fascin homolog like)	Passed	BG774457 Entrez UniGene	1656271	pINCY

25	3	-3.6	**-4.3**	233	2.2	62†	995	836	11.2	62	A	5	0216AKON	Internal_Control_G				
26	4420	-3.6	**-4.3**	130	2.0	81†	553	465	7.3	81	G	8	021VAGMQ	tumor necrosis factor (ligand) superfamily, member 4 (tax-transcriptionally activated glycoprotein 1, 34kD)	Multiple Bands	BE349175 Entrez UniGene	1512102	pINCY
27	4457	-3.3	**-4.0**	174	2.4	74†	688	578	9.4	74	C	10	0219AGMS	signal transducer and activator of transcription 5A	Passed	AI582321 Entrez UniGene	606641	pSport1
28	4998	-3.2	**-3.8**	177	2.5	88	664	558	10.3	88	G	12	021PAGNE	early growth response 2 (Krox-20 (Drosophila) homolog)	Passed	BG743293 Entrez UniGene	3603037	pINCY
29	538	-3.1	**-3.7**	712	5.7	58	2600	2185	24.8	58	C	7	021NAGL7	glycoprotein, synaptic 2	Passed	BG282184 Entrez UniGene	3721920	pINCY
30	1476	-3.0	**-3.6**	1147	10.8	100	4125	3466	56.0	100	C	11	021RAGMA	baculoviral IAP repeat-containing 3	Passed	U37546 Entrez UniGene	1513214	pINCY
31	1539	-2.8	**-3.3**	116	1.7	63†	382	321	4.5	63	A	5	021CAGMD	aminolevulinate, delta-, synthase 1	Passed	BF969214 Entrez UniGene	943569	pSport1

32	10171	-2.8	**-3.3**	149	2.3	81†	494	415	7.2	81	B	2	021MAKNL	Control: Ratio 1:3 (Cy3:Cy5)				
33	7621	-2.8	**-3.3**	161	2.3	78†	533	448	7.9	78	B	1	021MAKNL	Control: Ratio 1:3 (Cy3:Cy5)				
34	2553	-2.8	**-3.3**	321	2.9	44	1066	896	12.5	44	A	6	0216AKON	Internal_Control_G				
35	8990	-2.8	**-3.3**	102	1.9	96†	334	281	5.6	96	H	4	0218AGM4	potassium voltage-gated channel, KQT-like subfamily, member 2	Passed	AF110020 Entrez UniGene	617878	pSport1
36	187	-2.6	**-3.2**	693	6.2	65	2184	1835	32.6	65	G	1	021XAGKS	DnaJ (Hsp40) homolog, subfamily A, member 1	Passed	NM_001539 Entrez UniGene	1926883	pSport1
37	8846	-2.7	**-3.2**	291	3.4	65	922	775	12.4	65	H	4	0219AGLY	ninjurin 1	Passed	BG530047 Entrez UniGene	2927362	pINCY
38	5071	-2.5	**-3.0**	163	2.3	73†	486	408	6.7	73	A	2	021MAKNL	Control: Ratio 1:3 (Cy3:Cy5)				
39	5101	-2.4	**-2.9**	389	3.0	49	1122	943	12.1	49	B	1	0216AKON	Internal_Control_E				
40	1653	-2.4	**-2.9**	218	2.4	96†	634	533	8.6	96	G	5	0214AGMH	BCL2-related protein A1	Passed	BF677029 Entrez UniGene	2555673	pINCY

41	5205	-2.4	**-2.8**	3504	28.4	51	9981	8387	111.4	51	D	5	021CAGKP	colony stimulating factor 2 (granulocyte-macrophage)	Passed	BE669962 Entrez UniGene	3297733	pI NCY
42	7982	-2.4	**-2.8**	394	4.2	67	1103	927	15.5	67	H	4	0213AGKY	regulator of G-protein signalling 1	Passed	S59049 Entrez UniGene	1728022	pI NCY
43	8229	-2.3	**-2.8**	129	2.2	87†	361	303	5.6	87	B	6	0211AGL9	syndecan 4 (amphiglycan, ryudocan)	Passed	AI582184 Entrez UniGene	5183574	pI NCY
44	2521	-2.3	**-2.8**	188	2.3	74†	518	435	6.5	74	A	1	021MAKNL	Control: Ratio 1:3 (Cy3:Cy5)				
45	5156	-2.3	**-2.8**	344	3.5	68	957	804	10.8	68	D	3	021YAGKN	BTG family, member 2	No Amplification	NM_006763 Entrez UniGene	1598617	pI NCY
46	7651	-2.4	**-2.8**	272	2.7	55	769	646	8.8	55	B	2	0216AKON	Internal_Control_E				
47	9527	-2.4	**-2.8**	335	4.0	91	950	798	14.3	91	B	10	0212AGMR	tumor necrosis factor receptor superfamily, member 6	Passed	AL542093 Entrez UniGene	2205246	pI NCY
48	2551	-2.3	**-2.8**	339	2.8	55	941	791	10.5	55	A	2	0216AKON	Internal_Control_E				
49	6903	-2.4	**-2.8**	1476	12.4	91	4139	3478	42.4	91	B	5	021HAGMO	thioredoxin	Passed	AV763087 Entrez UniGene	2606240	pI NCY

50	7333	-2.4	**-2.8**	719	6.8	100	2016	1694	26.3	100	B	1	021SAGN6	suppression of tumorigenicity 14 (colon carcinoma, matriptase, epithin)	Passed	AL548113 Entrez UniGene	478960	pSport1
51	2156	-2.3	**-2.8**	508	4.4	100	1398	1175	16.7	100	G	3	0210AGN2	baculoviral IAP repeat-containing 2	Passed	U37547 Entrez UniGene	1810777	pINCY
52	33	-2.3	**-2.7**	553	4.4	70	1517	1275	17.1	70	C	5	021RAGKM	neutrophil cytosolic factor 2 (65kD, chronic granulomatous disease, autosomal 2)	Passed	NM_000433 Entrez UniGene	1556718	pINCY
53	9342	-2.2	**-2.6**	728	7.3	67	1885	1584	26.1	67	D	12	021IAGMJ	peptidyl prolyl isomerase H (cyclophilin H)	Passed	BF794821 Entrez UniGene	3667096	pINCY
54	5140	-2.2	**-2.6**	456	3.8	62	1196	1005	13.0	62	F	7	021RAGKM	ring finger protein 1	Passed	AL576514 Entrez UniGene	1879727	pINCY
55	1581	-2.2	**-2.6**	835	6.8	75	2198	1847	26.3	75	G	5	021JAGME	coactivator-associated arginine methyltransferase-1	Passed	BG830500 Entrez UniGene	2807446	pINCY
56	3525	-2.2	**-2.6**	400	4.1	100	1029	865	12.7	100	E	6	021IAGLP	HtrA-like serine protease	Passed	AL577683 Entrez UniGene	1718257	pINCY

57	9661	-2.1	**-2.6**	356	3.8	89	908	763	11.6	89	H	2	0211AGMW	cyclin-dependent kinase inhibitor 1A (p21, Cip1)	Passed	L26165 Entrez UniGene	1804548	pINCY
58	8307	-2.1	**-2.5**	244	2.9	68	599	503	8.2	68	D	6	021ZAGLC	squalene epoxidase	No Amplification	AF098865 Entrez UniGene	859645	pSport1
59	2275	-2.1	**-2.5**	91	1.5	71†	223	187	2.8	71	G	1	021ZAGN7	JAK binding protein	Passed	BE514365 Entrez UniGene	445246	pBlue
60	2138	-2.1	**-2.5**	299	3.4	73	759	638	10.7	73	A	3	0210AGN2	mevalonate (diphospho) decarboxylase	Passed	BG324529 Entrez UniGene	1711364	pINCY
61	67	-2.1	**-2.5**	3360	25.9	60	8491	7135	114.3	60	G	1	021YAGKN	lymphocyte cytosolic protein 1 (L-plastin)	Passed	BC007673 Entrez UniGene	1363074	pINCY
62	9470	-2.1	**-2.5**	407	4.5	100	1015	853	14.8	100	H	4	021HAGMO	tyrosine kinase 2	Passed	NM_003331 Entrez UniGene	1831805	pINCY
63	3611	-2.1	**-2.5**	616	6.5	66	1533	1288	20.2	66	A	10	021AAGLT	G-rich RNA sequence binding factor 1	Passed	BF034561 Entrez UniGene	1931925	pINCY

64	3735	-2.1	**-2.5**	789	7.8	100	1974	1659	26.5	100	C	6	0219AGLY	intercellular adhesion molecule 1 (CD54), human rhinovirus receptor	Passed	M24283 Entrez UniGene	1556061	pINCY
65	7320	-2.1	**-2.5**	162	2.2	91†	407	342	5.5	91	D	11	021LAGN5	GTP-binding protein overexpressed in skeletal muscle	Passed	AW297828 Entrez UniGene	450618	pBlue
66	3294	-2.0	**-2.4**	585	5.8	100	1422	1195	18.1	100	G	12	021KAGLF	protein geranylgeranyltransferase type I, beta subunit	Passed	AA481712 Entrez UniGene	815861	pSport1
67	4916	-2.0	**-2.4**	797	8.2	65	1879	1579	27.3	65	E	4	0214AGNB	translocase of inner mitochondrial membrane 17 (yeast) homolog A	Passed	BG506029 Entrez UniGene	2458933	pINCY
68	6204	-2.0	**-2.4**	573	5.2	100	1348	1133	16.3	100	H	11	021HAGLU	small inducible cytokine subfamily A (Cys-Cys), member 20	Passed	NM_004591 Entrez UniGene	2220923	pINCY
69	9619	-2.0	**-2.4**	274	3.4	86	662	556	9.3	86	B	2	021UAGMV	tryptophanyl-tRNA synthetase	Passed	BF795451 Entrez UniGene	1846209	pSport1
70	2295	-2.0	**-2.4**	193	2.7	82	468	393	7.4	82	E	5	0216AGN8	prostaglandin E receptor 4 (subtype EP4)	Passed	NM_000958 Entrez UniGene	1631793	pINCY

71	6097	-2.0	**-2.4**	140	2.0	65†	330	277	4.5	65	F	1	021PAGLQ	hypothetical protein, clone 2746033	Passed	BE745844 Entrez UniGene	1923769	pSport1
72	9828	-2.0	**-2.4**	97	1.9	91†	234	197	4.3	91	F	12	0217AGN3	vesicle-associated membrane protein 5 (myobrevin)	Passed	AF151025 Entrez UniGene	122826	pBlue
73	2595	-1.9	**-2.3**	576	4.5	65	1308	1099	14.4	65	G	6	021RAGKM	integrin, beta 2 (antigen CD18 (p95), lymphocyte function-associated antigen 1; macrophage antigen 1 (mac-1) beta subunit)	Passed	BC005861 Entrez UniGene	1871113	pINCY
74	10091	-1.9	**-2.3**	262	2.9	65	590	496	7.7	65	F	10	021PAGNE	ELL-RELATED RNA POLYMERASE II, ELONGATION FACTOR	Passed	NM_012081 Entrez UniGene	1281473	pINCY
75	5187	-1.9	**-2.3**	645	4.9	60	1454	1222	13.3	60	F	5	0215AGKO	flap structure-specific endonuclease 1	Passed	AU142907 Entrez UniGene	2050085	pINCY
76	2150	-1.9	**-2.3**	971	8.1	61	2212	1859	25.8	61	E	3	0210AGN2	heat shock 70kD protein 5 (glucose-regulated protein, 78kD)	Passed	AI878886 Entrez UniGene	2884613	pINCY

77	4561	-2.0	**-2.3**	227	3.0	75	532	447	8.5	75	G	2	0211AGMW	chitobiase, di-N-acetyl-	Passed	AA688097 Entrez UniGene	2879077	pINCY
78	1420	-1.9	**-2.2**	449	4.0	100	1007	846	11.6	100	A	7	0210AGM8	SH3-domain binding protein 5 (BTK-associated)	Passed	BG030766 Entrez UniGene	2170638	pINCY
79	7696	-1.9	**-2.2**	213	2.4	68†	474	398	6.4	68	H	8	021RAGKM	phorbol-12-myristate-13-acetate-induced protein 1	Passed	BG776688 Entrez UniGene	1931117	pINCY
80	2654	-1.9	**-2.2**	729	6.2	75	1627	1367	20.8	75	C	4	021CAGKP	vacuolar protein sorting 41 (yeast homolog)	No Amplification	NM_014396 Entrez UniGene	2910949	pINCY
81	2975	-1.8	**-2.1**	114	1.9	93†	244	205	3.8	93	E	10	021OAGL2	syndecan 4 (amphiglycan, ryudocan)	No Amplification	NM_002999 Entrez UniGene	3214670	pINCY
82	1990	-1.7	**-2.1**	115	1.9	91†	239	201	3.8	91	G	7	021UAGMV	protein kinase C, delta	No Amplification	L07861 Entrez UniGene	613603	pSport1

83	4934	-1.7	**-2.1**	138	2.1	66†	284	239	4.1	66	C	4	021BAGNC	cyclin-dependent kinase 7 (homolog of Xenopus MO15 cdk-activating kinase)	Passed	BE887969 Entrez UniGene	1558108	pINCY
84	687	-1.7	**-2.1**	249	2.6	75	515	433	6.6	75	E	5	0216AGLD	UDP-N-acteylglucosamine pyrophosphorylase 1	Passed	AL520091 Entrez UniGene	1997038	pSport1
85	5039	-1.8	**-2.1**	251	3.1	73	525	441	7.4	73	E	10	0213AGNG	reticulocalbin 1, EF-hand calcium binding domain	Passed	NM_002901 Entrez UniGene	2057296	pSport1
86	182	-1.8	**-2.1**	478	4.4	65	1022	859	13.6	65	E	3	021XAGKS	v-jun avian sarcoma virus 17 oncogene homolog	Passed	AI078377 Entrez UniGene	1969563	pSport1
87	9135	-1.7	**-2.1**	983	9.5	100	2022	1699	26.7	100	H	6	021RAGMA	serine (or cysteine) proteinase inhibitor, clade E (nexin, plasminogen activator inhibitor type 1), member 1	Passed	BE812315 Entrez UniGene	1445767	pINCY
88	3217	-1.8	**-2.1**	1599	13.2	100	3401	2858	38.6	100	G	2	021ZAGLC	synaptogyrin 2	Passed	AL545227 Entrez UniGene	983008	pSport1

89	10027	-1.7	**-2.1**	3229	26.8	77	6660	5597	81.0	77	B	2	021BAGNC	vimentin	Passed	AL572054 Entrez UniGene	1522716	pINCY
90	7030	-1.7	**-2.0**	482	4.9	100	953	801	13.1	100	D	7	021GAGMT	basement membrane-induced gene	Passed	NM_004848 Entrez UniGene	1840811	pSport1
91	1315	-1.7	**-2.0**	600	5.0	100	1194	1003	13.7	100	G	1	0211AGM3	dual specificity phosphatase 5	Passed	U16996 Entrez UniGene	1734561	pINCY
92	7043	-1.6	**-2.0**	334	3.4	67	656	551	8.9	67	H	9	021GAGMT	BTG family, member 3	Passed	BG110736 Entrez UniGene	637576	pSport1
93	2888	-1.7	**-2.0**	192	2.7	92	392	329	6.6	92	A	4	021AAGKZ	oxygen regulated protein (150kD)	Passed	AI969119 Entrez UniGene	2398659	pINCY
94	8729	-1.7	**-2.0**	117	2.1	96†	239	201	4.4	96	H	10	021AAGLT	plastin 3 (T isoform)	Passed	BF683154 Entrez UniGene	1402228	pINCY
95	7464	-1.7	**-2.0**	1053	9.7	100	2078	1746	27.1	100	D	11	0214AGNB	proliferating cell nuclear antigen	Passed	AA523378 Entrez UniGene	2781405	pINCY

96	2233	-1.7	**-2.0**	459	4.1	68	916	770	10.6	68	A	1	021SAGN6	ADP-ribosylation factor-like 3	Passed	BI013116 Entrez UniGene	67300	pBlue
97	4100	-1.7	**-2.0**	818	6.9	62	1628	1368	19.7	62	E	4	021CAGMD	ferritin, heavy polypeptide 1	Passed	BE878314 Entrez UniGene	27775	pBlue
98	7377	-1.7	**-2.0**	754	7.6	100	1516	1274	21.5	100	H	5	021ZAGN7	endothelin converting enzyme 1	Passed	NM_001397 Entrez UniGene	1963819	pSport1
99	7018	-1.6	**-1.9**	257	2.9	89	480	403	6.8	89	H	7	0219AGMS	preferentially expressed antigen in melanoma	Passed	AI017284 Entrez UniGene	2007554	pBlue
100	4844	-1.6	**-1.9**	762	6.3	65	1440	1210	16.9	65	E	4	0216AGN8	cell division cycle 42 (GTP-binding protein, 25kD)	Passed	AV722422 Entrez UniGene	2834543	pINCY

† Probe 1 did not meet selection criteria

Order LifeArray clones

Next 100

1 2 3 4 5 6 7 8 9 10 11 12 13 14 15 16 17 18 19 20 21 22 23 24 25 26 27 28 29 30 31 32 33 34 35 36 37 38 39 40 41 42 43 44 45 46 47 48 49 50 51 52 53 54 55 56 57 58 59 60 61 62 63 64 65 66 67 68 69 70 71 72 73 74 75 76 77 78 79 80 81 82 83 84 85 86 87 88 89 90 91 92 93 94

Entire List in plain text (long -- 1.91 MB)

PDF image of LifeArray (long -- 2.43 MB)

LifeArray color bar:

To save your LifeArray™ results on your computer, use the 'Plain Text' option to display your results, then save them on your computer with your browser's save feature. We will also provide your LifeArray results on a CD-ROM for a nominal fee. Please contact our Technical Support group if you need any assistance.

We guarantee that your LifeArray results will remain on the server for 90 days after it was first uploaded. After that, we may remove and archive your LifeArray results at our discretion. Please contact our Technical Support group if you need any archived LifeArray results restored to our server.

In order to view or print Adobe® Acrobat® PDF files, you need the Adobe Acrobat Reader. If you do not already have it installed, you can obtain it for free from the Adobe web site .

If you have questions about the documents or have difficulty downloading the Acrobat Reader, please contact us.

Download the LifeArray Frequently Asked Questions list in HTML format.

Download the Human UniGEM V Frequently Asked Questions list in HTML format.

Download the LifeArray Control Plate Document in HTML format.

Adobe and Acrobat are trademarks of Adobe Systems Incorporated.

Sort Again:

**Username:**	nature
**Password:**	
**Sort Order:**	Ascending Descending
**Sort By:**	Location<br>Diff Expr<br>Balanced Diff Expr<br>P1 Signal<br>P1 S/B<br>P2 Balanced Signal<br>P2 Signal<br>P2 S/B<br>Plate ID/Row/Col<br>Gene Name
**Plate ID:**	
**Gene Name:**	

LifeArray Products
Incyte Genomics Reagents Home
