# Supplementary material for: Trans-Activation of the Coactivator-Associated Arginine Methyltransferase 1 (Carm1) Gene by the Oncogene Product Tax of Human T-Cell Leukemia Virus Type 1
Source: Genes (Basel). 2024 May 27;15(6):698. doi: 10.3390/genes15060698 (PMC11202806; doi:10.3390/genes15060698)
Supplement: Supplementary file 1 [file genes-15-00698-s001.zip › Supplementary Table S2]

Human UniGene 1 Results

## Human UniGene 1 Results

Experiment Results Generated by GEMTools 2.5  
Client: Genome\_Systems  
Export Date: Oct 12, 2001  


---

|  |  |
| --- | --- |
| GEM | 022JC38J |
| Balance Coefficient | 1.19 |
| Minimum S/B | 2.5 |
| Minimum Area | 40% |
| Probe 1 | 123YA1BU |
| P1 Description | d17/5 |
| Probe 2 | 1235A1BV |
| P2 Description | wt |

---

Report sorted by **Balanced Diff Expr** in **Ascending** order  
Ranks from **101** to **200**

### Order LifeArray clones

Legend

| Rank | Location | Diff Expr | Balanced Diff Expr | P1 Signal | P1 S/B | P1 Area % | P2 Balanced Signal | P2 Signal | P2 S/B | P2 Area % | Plate Row | Plate Col | Plate ID | Gene Name | PCR Status | GenBank Id | Clone Id (Sequence) | Vector |
| --- | --- | --- | --- | --- | --- | --- | --- | --- | --- | --- | --- | --- | --- | --- | --- | --- | --- | --- |
| 101 | 8632 | -1.6 | **-1.9** | 371 | 4.5 | 85 | 703 | 591 | 10.7 | 85 | H | 8 | 021IAGLP | SWI/SNF related, matrix associated, actin dependent regulator of chromatin, subfamily a, member 2 | Passed | X72889 Entrez UniGene | 2944175 | pINCY |
| 102 | 3089 | -1.6 | **-1.9** | 142 | 2.1 | 83**†** | 263 | 221 | 3.9 | 83 | C | 10 | 021NAGL7 | fascin (Strongylocentrotus purpuratus) homolog 2 (actin-bundling protein, retinal) | Passed | NM\_012418 Entrez UniGene | 4855125 | pINCY |
| 103 | 6438 | -1.6 | **-1.9** | 480 | 5.2 | 100 | 934 | 785 | 13.9 | 100 | F | 11 | 0218AGM4 | thymidine kinase 1, soluble | Passed | BE746447 Entrez UniGene | 2055926 | pSport1 |
| 104 | 629 | -1.6 | **-1.9** | 496 | 4.4 | 100 | 933 | 784 | 12.4 | 100 | A | 9 | 021SAGLB | UDP-N-acetyl-alpha-D-galactosamine:polypeptide N-acetylgalactosaminyltransferase 2 (GalNAc-T2) | Passed | AL580930 Entrez UniGene | 516735 | pSport1 |
| 105 | 8186 | -1.6 | **-1.9** | 514 | 5.8 | 98 | 997 | 838 | 14.7 | 98 | D | 4 | 021NAGL7 | potassium intermediate/small conductance calcium-activated channel, subfamily N, member 4 | Passed | BG741215 Entrez UniGene | 2793840 | pINCY |
| 106 | 4503 | -1.6 | **-1.9** | 545 | 5.0 | 67 | 1014 | 852 | 11.7 | 67 | C | 6 | 021NAGMU | lamin B2 | Passed | AU130032 Entrez UniGene | 2414632 | pINCY |
| 107 | 527 | -1.6 | **-1.9** | 859 | 7.0 | 100 | 1626 | 1366 | 19.0 | 100 | G | 9 | 021GAGL6 | tumor rejection antigen (gp96) 1 | Passed | AV655628 Entrez UniGene | 3624631 | pINCY |
| 108 | 5144 | -1.6 | **-1.9** | 974 | 6.2 | 44 | 1833 | 1540 | 14.9 | 44 | H | 3 | 021RAGKM | protein disulfide isomerase related protein (calcium-binding protein, intestinal-related) | Passed | NM\_004911 Entrez UniGene | 1824957 | pINCY |
| 109 | 63 | -1.6 | **-1.9** | 1283 | 10.1 | 65 | 2395 | 2013 | 31.0 | 65 | E | 5 | 021YAGKN | FXYD domain-containing ion transport regulator 2 | Passed | BG166035 Entrez UniGene | 2380042 | pINCY |
| 110 | 7557 | -1.6 | **-1.9** | 1387 | 12.0 | 65 | 2658 | 2234 | 34.8 | 65 | D | 5 | 021WAGNF | malate dehydrogenase 1, NAD (soluble) | Passed | NM\_005917 Entrez UniGene | 487539 | pBlue |
| 111 | 104 | -1.6 | **-1.9** | 2197 | 13.1 | 55 | 4075 | 3424 | 35.9 | 55 | C | 3 | 021CAGKP | KIAA0084 protein | Passed | AL561563 Entrez UniGene | 2697959 | pINCY |
| 112 | 415 | -1.6 | **-1.9** | 3427 | 27.0 | 62 | 6562 | 5514 | 87.2 | 62 | C | 1 | 021OAGL2 | RNA-binding protein regulatory subunit | Passed | BG715880 Entrez UniGene | 1879921 | pINCY |
| 113 | 7301 | -1.6 | **-1.9** | 227 | 2.4 | 79**†** | 433 | 364 | 5.5 | 79 | F | 9 | 021EAGN4 | hypothetical protein KIAA1165 | No Amplification | R82692 Entrez UniGene | 55889 | pBlue |
| 114 | 3622 | -1.5 | **-1.8** | 152 | 2.2 | 100**†** | 273 | 229 | 4.3 | 100 | E | 8 | 021AAGLT | arginine-rich, mutated in early stage tumors | Passed | AA974308 Entrez UniGene | 1618455 | pINCY |
| 115 | 3787 | -1.5 | **-1.8** | 153 | 2.1 | 92**†** | 269 | 226 | 4.0 | 92 | E | 2 | 021GAGM0 | KIAA0974 protein | Passed | BE669938 Entrez UniGene | 4540 | pBlue |
| 116 | 3017 | -1.5 | **-1.8** | 173 | 2.2 | 86**†** | 307 | 258 | 4.4 | 86 | C | 10 | 0212AGL4 | platelet activating receptor homolog | Passed | NM\_013308 Entrez UniGene | 3879095 | pINCY |
| 117 | 6495 | -1.5 | **-1.8** | 154 | 2.1 | 86**†** | 276 | 232 | 4.1 | 86 | B | 5 | 021TAGM7 | Homo sapiens cDNA: FLJ21897 fis, clone HEP03447, highly similar to AF052178 Homo sapiens clone 24523 mRNA sequence | Passed | BE259271 Entrez UniGene | 2518964 | pINCY |
| 118 | 6374 | -1.5 | **-1.8** | 1481 | 14.2 | 100 | 2600 | 2185 | 36.2 | 100 | B | 3 | 021UAGM2 | FYN-binding protein (FYB-120/130) | Passed | AF198052 Entrez UniGene | 1614872 | pINCY |
| 119 | 9411 | -1.5 | **-1.8** | 268 | 2.9 | 88 | 471 | 396 | 5.7 | 88 | D | 6 | 0213AGMM | synaptophysin-like protein | Passed | S72481 Entrez UniGene | 1798283 | pINCY |
| 120 | 7570 | -1.5 | **-1.8** | 287 | 3.3 | 82 | 512 | 430 | 7.6 | 82 | H | 7 | 021WAGNF | S-adenosylhomocysteine hydrolase-like 1 | Passed | AL527927 Entrez UniGene | 1739627 | pSport1 |
| 121 | 7577 | -1.5 | **-1.8** | 297 | 3.2 | 70 | 528 | 444 | 7.6 | 70 | B | 9 | 0213AGNG | CD58 antigen, (lymphocyte function-associated antigen 3) | Passed | NM\_001779 Entrez UniGene | 342285 | pBlue |
| 122 | 1322 | -1.5 | **-1.8** | 212 | 2.5 | 96 | 383 | 322 | 6.0 | 96 | A | 3 | 0218AGM4 | nuclear matrix protein p84 | Passed | AV713026 Entrez UniGene | 22715 | pBlue |
| 123 | 6786 | -1.5 | **-1.8** | 2578 | 22.8 | 55 | 4618 | 3881 | 56.4 | 55 | B | 11 | 021IAGMJ | tubulin, beta polypeptide | Passed | BG762520 Entrez UniGene | 3334367 | pINCY |
| 124 | 2716 | -1.5 | **-1.8** | 1160 | 10.8 | 66 | 2048 | 1721 | 28.3 | 66 | G | 8 | 021QAGKR | diazepam binding inhibitor (GABA receptor modulator, acyl-Coenzyme A binding protein) | Passed | AA843364 Entrez UniGene | 2060396 | pSport1 |
| 125 | 4316 | -1.5 | **-1.8** | 1114 | 9.6 | 100 | 2040 | 1714 | 23.9 | 100 | E | 4 | 0213AGMM | transporter 1, ATP-binding cassette, sub-family B (MDR/TAP) | Passed | NM\_000593 Entrez UniGene | 1634279 | pINCY |
| 126 | 488 | -1.5 | **-1.8** | 641 | 5.7 | 68 | 1157 | 972 | 12.9 | 68 | C | 3 | 0219AGL5 | TNF receptor-associated factor 2 | Passed | BG677004 Entrez UniGene | 3226017 | pINCY |
| 127 | 1926 | -1.5 | **-1.8** | 369 | 4.2 | 85 | 655 | 550 | 10.6 | 85 | A | 11 | 021GAGMT | leptin (murine obesity homolog) | Passed | NM\_000230 Entrez UniGene | 1961822 | pSport1 |
| 128 | 1273 | -1.5 | **-1.8** | 387 | 3.9 | 93 | 679 | 571 | 10.2 | 93 | A | 1 | 021UAGM2 | grancalcin, EF-hand calcium-binding protein | Passed | BG545676 Entrez UniGene | 1671852 | pINCY |
| 129 | 1177 | -1.5 | **-1.8** | 406 | 4.1 | 85 | 735 | 618 | 10.1 | 85 | A | 1 | 0219AGLY | early growth response 1 | Passed | AL553329 Entrez UniGene | 1705208 | pINCY |
| 130 | 5138 | -1.5 | **-1.8** | 453 | 3.7 | 62 | 815 | 685 | 8.4 | 62 | F | 3 | 021RAGKM | ribosomal protein S6 kinase, 90kD, polypeptide 1 | Passed | NM\_002953 Entrez UniGene | 1822236 | pINCY |
| 131 | 8359 | -1.5 | **-1.8** | 462 | 4.3 | 88 | 833 | 700 | 9.2 | 88 | F | 2 | 021DAGLE | protein kinase, cAMP-dependent, regulatory, type I, beta | Passed | BF940317 Entrez UniGene | 1926688 | pSport1 |
| 132 | 7799 | -1.5 | **-1.8** | 519 | 4.5 | 50 | 925 | 777 | 10.4 | 50 | B | 10 | 021QAGKR | spermine synthase | Passed | BG535109 Entrez UniGene | 2061432 | pSport1 |
| 133 | 6370 | -1.5 | **-1.8** | 1054 | 10.6 | 100 | 1853 | 1557 | 27.0 | 100 | H | 7 | 021NAGM1 | SEC14 (S. cerevisiae)-like 1 | Passed | NM\_003003 Entrez UniGene | 1879956 | pINCY |
| 134 | 6529 | -1.5 | **-1.8** | 540 | 5.6 | 100 | 988 | 830 | 14.2 | 100 | F | 1 | 0210AGM8 | solute carrier family 29 (nucleoside transporters), member 1 | Passed | BI088219 Entrez UniGene | 2594080 | pINCY |
| 135 | 9461 | -1.5 | **-1.8** | 135 | 2.1 | 88**†** | 244 | 205 | 3.8 | 88 | D | 10 | 021HAGMO | cathepsin H | Passed | BC002479 Entrez UniGene | 1749417 | pINCY |
| 136 | 4361 | -1.5 | **-1.8** | 125 | 1.9 | 83**†** | 223 | 187 | 3.4 | 83 | C | 10 | 021HAGMO | nuclear receptor subfamily 4, group A, member 1 | Passed | NM\_002135 Entrez UniGene | 1958560 | pINCY |
| 137 | 6375 | -1.5 | **-1.8** | 921 | 8.7 | 100 | 1695 | 1424 | 23.3 | 100 | B | 5 | 021UAGM2 | v-rel avian reticuloendotheliosis viral oncogene homolog B (nuclear factor of kappa light polypeptide gene enhancer in B-cells 3) | Passed | BG748809 Entrez UniGene | 1859449 | pINCY |
| 138 | 10180 | -1.6 | **-1.8** | 845 | 9.0 | 62 | 1561 | 1312 | 24.9 | 62 | D | 8 | 021MAKNL | Control: Sensitivity 2000pg |  |  |  |  |
| 139 | 3168 | -1.5 | **-1.8** | 300 | 3.0 | 87 | 546 | 459 | 6.4 | 87 | E | 12 | 021LAGLA | DNA segment on chromosome X (unique) 9879 expressed sequence | Passed | AI360458 Entrez UniGene | 2585358 | pINCY |
| 140 | 3805 | -1.5 | **-1.8** | 624 | 6.4 | 66 | 1147 | 964 | 17.1 | 66 | C | 2 | 021NAGM1 | tetracycline transporter-like protein | Passed | L11669 Entrez UniGene | 1856947 | pINCY |
| 141 | 1 | -1.5 | **-1.8** | 547 | 3.5 | 55 | 1004 | 844 | 10.3 | 55 | A | 1 | 0216AKON | Internal\_Control\_E |  |  |  |  |
| 142 | 8522 | -1.5 | **-1.8** | 109 | 2.0 | 97**†** | 198 | 166 | 3.5 | 97 | D | 4 | 021QAGLL | hypothetical protein FLJ23231 | Passed | AA640102 Entrez UniGene | 2348706 | pINCY |
| 143 | 6701 | -1.5 | **-1.7** | 1063 | 7.7 | 65 | 1858 | 1561 | 20.3 | 65 | F | 9 | 021QAGMF | SH2 domain protein 2A | Passed | NM\_003975 Entrez UniGene | 504273 | pBlue |
| 144 | 7630 | -1.5 | **-1.7** | 1477 | 14.0 | 61 | 2567 | 2157 | 36.4 | 61 | D | 7 | 021MAKNL | Control: Sensitivity 2000pg |  |  |  |  |
| 145 | 7083 | -1.4 | **-1.7** | 2448 | 23.3 | 86 | 4100 | 3445 | 54.0 | 86 | F | 5 | 021UAGMV | tumor necrosis factor receptor superfamily, member 9 | Passed | BG436824 Entrez UniGene | 191843 | pBlue |
| 146 | 1102 | -1.4 | **-1.7** | 895 | 7.1 | 100 | 1482 | 1245 | 18.8 | 100 | G | 7 | 021HAGLU | nuclear factor of kappa light polypeptide gene enhancer in B-cells inhibitor, epsilon | Passed | BG469491 Entrez UniGene | 2748942 | pINCY |
| 147 | 10047 | -1.4 | **-1.7** | 910 | 8.5 | 62 | 1509 | 1268 | 20.2 | 62 | H | 6 | 021BAGNC | pericentrin 2 (kendrin) | Passed | AB007862 Entrez UniGene | 1655365 | pINCY |
| 148 | 9493 | -1.5 | **-1.7** | 6971 | 60.6 | 49 | 12108 | 10175 | 155.2 | 49 | H | 2 | 021OAGMP | x 006 protein | Passed | BF445491 Entrez UniGene | 440017 | pBlue |
| 149 | 8042 | -1.4 | **-1.7** | 19343 | 175.3 | 51 | 32332 | 27170 | 412.7 | 51 | D | 4 | 021HAGL1 | nuclear receptor subfamily 1, group H, member 2 | Passed | BE878950 Entrez UniGene | 2581075 | pINCY |
| 150 | 3741 | -1.4 | **-1.7** | 909 | 8.2 | 100 | 1509 | 1268 | 18.6 | 100 | E | 6 | 0219AGLY | tumor necrosis factor receptor superfamily, member 14 (herpesvirus entry mediator) | Multiple Bands | BC002794 Entrez UniGene | 2121653 | pINCY |
| 151 | 9008 | -1.4 | **-1.7** | 2975 | 26.9 | 82 | 4985 | 4189 | 66.5 | 82 | F | 4 | 021FAGM5 | peroxiredoxin 1 | Passed | BG612736 Entrez UniGene | 3942594 | pINCY |
| 152 | 9528 | -1.4 | **-1.7** | 345 | 4.2 | 92 | 595 | 500 | 9.3 | 92 | B | 12 | 0212AGMR | metallothionein 1L | Passed | BG928106 Entrez UniGene | 2513883 | pINCY |
| 153 | 1089 | -1.4 | **-1.7** | 170 | 2.1 | 59**†** | 292 | 245 | 3.8 | 59 | C | 5 | 021HAGLU | solute carrier family 8 (sodium/calcium exchanger), member 1 | Passed | NM\_021097 Entrez UniGene | 2880435 | pINCY |
| 154 | 6796 | -1.4 | **-1.7** | 844 | 7.4 | 62 | 1448 | 1217 | 17.4 | 62 | F | 7 | 021IAGMJ | cytochrome b-245, alpha polypeptide | Passed | BG751568 Entrez UniGene | 4173205 | pINCY |
| 155 | 2932 | -1.5 | **-1.7** | 757 | 7.3 | 100 | 1315 | 1105 | 16.8 | 100 | G | 8 | 021AAGL0 | macrophage myristoylated alanine-rich C kinase substrate | Passed | AL534743 Entrez UniGene | 2135733 | pINCY |
| 156 | 4287 | -1.4 | **-1.7** | 718 | 7.4 | 100 | 1225 | 1029 | 17.1 | 100 | C | 6 | 021WAGML | tubulin, beta, 5 | Passed | AL536237 Entrez UniGene | 1486358 | pINCY |
| 157 | 1774 | -1.4 | **-1.7** | 554 | 4.7 | 100 | 938 | 788 | 10.7 | 100 | G | 7 | 0213AGMM | growth arrest and DNA-damage-inducible, alpha | Passed | AI935984 Entrez UniGene | 1702350 | pINCY |
| 158 | 5390 | -1.4 | **-1.7** | 432 | 4.3 | 75 | 745 | 626 | 10.2 | 75 | B | 3 | 021WAGKX | modulator recognition factor I | Passed | M62324 Entrez UniGene | 1568724 | pINCY |
| 159 | 117 | -1.4 | **-1.7** | 3127 | 24.0 | 53 | 5178 | 4351 | 65.0 | 53 | G | 5 | 021CAGKP | LIM domain kinase 1 | Passed | NM\_002314 Entrez UniGene | 3373632 | pINCY |
| 160 | 8372 | -1.4 | **-1.7** | 368 | 4.1 | 92 | 616 | 518 | 8.8 | 92 | B | 4 | 021KAGLF | keratin 7 | Passed | BI094014 Entrez UniGene | 1962141 | pSport1 |
| 161 | 6631 | -1.4 | **-1.7** | 319 | 3.7 | 93 | 528 | 444 | 8.4 | 93 | H | 1 | 0215AGMC | uridine monophosphate synthetase (orotate phosphoribosyl transferase and orotidine-5'-decarboxylase) | Passed | AU133065 Entrez UniGene | 1816355 | pINCY |
| 162 | 1887 | -1.4 | **-1.7** | 295 | 3.0 | 73 | 502 | 422 | 6.4 | 73 | E | 5 | 0212AGMR | replication protein A3 (14kD) | Passed | BF030135 Entrez UniGene | 2451279 | pINCY |
| 163 | 8993 | -1.4 | **-1.7** | 264 | 3.3 | 87 | 449 | 377 | 7.7 | 87 | H | 10 | 0218AGM4 | KIAA0005 gene product | Passed | NM\_014670 Entrez UniGene | 2059208 | pSport1 |
| 164 | 9799 | -1.4 | **-1.7** | 259 | 2.8 | 68 | 430 | 361 | 5.8 | 68 | F | 2 | 0210AGN2 | methylenetetrahydrofolate dehydrogenase (NADP+ dependent), methenyltetrahydrofolate cyclohydrolase, formyltetrahydrofolate synthetase | Passed | BC001014 Entrez UniGene | 1297179 | pINCY |
| 165 | 8262 | -1.4 | **-1.7** | 226 | 2.7 | 82 | 383 | 322 | 5.4 | 82 | D | 12 | 021LAGLA | neural polypyrimidine tract binding protein | Passed | BG991819 Entrez UniGene | 2578983 | pINCY |
| 166 | 8033 | -1.5 | **-1.7** | 199 | 2.6 | 90 | 346 | 291 | 4.9 | 90 | H | 10 | 021AAGL0 | retinoid X receptor, alpha | Passed | X52773 Entrez UniGene | 2808269 | pINCY |
| 167 | 4534 | -1.5 | **-1.7** | 151 | 1.9 | 76**†** | 261 | 219 | 3.5 | 76 | E | 8 | 021UAGMV | E2F transcription factor 1 | Passed | AL121906 Entrez UniGene | 1920739 | pSport1 |
| 168 | 5991 | -1.4 | **-1.7** | 148 | 2.1 | 72**†** | 253 | 213 | 3.7 | 72 | B | 5 | 021XAGLM | HTPAP protein | Passed | AI765129 Entrez UniGene | 2626340 | pINCY |
| 169 | 3731 | -1.4 | **-1.6** | 174 | 2.5 | 98 | 282 | 237 | 5.0 | 98 | A | 10 | 0219AGLY | annexin A8 | Passed | BC008813 Entrez UniGene | 1911622 | pINCY |
| 170 | 3280 | -1.4 | **-1.6** | 165 | 2.4 | 92**†** | 269 | 226 | 4.2 | 92 | C | 8 | 021KAGLF | RNA helicase-related protein | Passed | BF679543 Entrez UniGene | 2757583 | pSport1 |
| 171 | 6994 | -1.3 | **-1.6** | 230 | 2.5 | 75 | 361 | 303 | 4.8 | 75 | H | 7 | 0212AGMR | vav 2 oncogene | Passed | AL045952 Entrez UniGene | 3744592 | pINCY |
| 172 | 9458 | -1.3 | **-1.6** | 280 | 3.1 | 90 | 445 | 374 | 5.7 | 90 | D | 4 | 021HAGMO | serine/threonine kinase 4 | Passed | NM\_006282 Entrez UniGene | 1510581 | pINCY |
| 173 | 2358 | -1.4 | **-1.6** | 164 | 2.0 | 65**†** | 268 | 225 | 3.4 | 65 | A | 11 | 0214AGNB | RAB9, member RAS oncogene family | Passed | BG535930 Entrez UniGene | 1746329 | pINCY |
| 174 | 7474 | -1.4 | **-1.6** | 335 | 3.9 | 86 | 543 | 456 | 8.5 | 86 | H | 7 | 0214AGNB | aldo-keto reductase family 1, member A1 (aldehyde reductase) | Passed | AW873466 Entrez UniGene | 1634342 | pINCY |
| 175 | 2349 | -1.3 | **-1.6** | 174 | 2.1 | 59**†** | 274 | 230 | 3.8 | 59 | G | 5 | 021XAGNA | biliverdin reductase A | Passed | AI765830 Entrez UniGene | 775330 | pSport1 |
| 176 | 725 | -1.4 | **-1.6** | 875 | 7.6 | 100 | 1443 | 1213 | 19.4 | 100 | A | 9 | 021KAGLF | chemokine (C-C motif) receptor 6 | Passed | AL121935 Entrez UniGene | 3190228 | pSport1 |
| 177 | 7445 | -1.3 | **-1.6** | 162 | 2.1 | 59**†** | 256 | 215 | 3.4 | 59 | F | 9 | 021XAGNA | thymopoietin | Passed | BF983366 Entrez UniGene | 56919 | pBlue |
| 178 | 4665 | -1.4 | **-1.6** | 480 | 5.1 | 66 | 775 | 651 | 9.8 | 66 | A | 6 | 021TAGN1 | insulin-like growth factor 2 (somatomedin A) | Passed | BG619049 Entrez UniGene | 2955178 | pINCY |
| 179 | 1549 | -1.3 | **-1.6** | 496 | 4.2 | 58 | 771 | 648 | 7.8 | 58 | E | 1 | 021CAGMD | ADP-ribosylation factor 4 | Passed | BG529241 Entrez UniGene | 65463 | pBlue |
| 180 | 6748 | -1.3 | **-1.6** | 130 | 2.1 | 75**†** | 207 | 174 | 3.5 | 75 | F | 7 | 0214AGMH | exonuclease 1 | Passed | AC004783 Entrez UniGene | 4385292 | pINCY |
| 181 | 7700 | -1.4 | **-1.6** | 1003 | 7.7 | 58 | 1642 | 1380 | 19.4 | 58 | B | 4 | 021YAGKN | tumor necrosis factor (ligand) superfamily, member 7 | Passed | BG170786 Entrez UniGene | 2017463 | pINCY |
| 182 | 4678 | -1.3 | **-1.6** | 528 | 5.5 | 78 | 833 | 700 | 11.0 | 78 | E | 8 | 021TAGN1 | proteasome (prosome, macropain) subunit, beta type, 6 | Passed | BF205294 Entrez UniGene | 2989852 | pINCY |
| 183 | 4526 | -1.4 | **-1.6** | 437 | 4.4 | 72 | 709 | 596 | 10.5 | 72 | C | 4 | 021UAGMV | benzodiazapine receptor (peripheral) | Passed | BE531172 Entrez UniGene | 1241680 | pSport1 |
| 184 | 4806 | -1.3 | **-1.6** | 214 | 2.3 | 56**†** | 342 | 287 | 4.2 | 56 | G | 12 | 021SAGN6 | cAMP responsive element modulator | Passed | AL117336 Entrez UniGene | 1921290 | pSport1 |
| 185 | 2828 | -1.4 | **-1.6** | 537 | 5.4 | 100 | 872 | 733 | 12.1 | 100 | E | 4 | 021PAGKW | intracellular hyaluronan-binding protein | Passed | AK025144 Entrez UniGene | 1755234 | pINCY |
| 186 | 9466 | -1.3 | **-1.6** | 601 | 6.5 | 100 | 933 | 784 | 13.4 | 100 | F | 8 | 021HAGMO | proteasome (prosome, macropain) subunit, alpha type, 4 | Passed | BG178069 Entrez UniGene | 1975642 | pINCY |
| 187 | 5163 | -1.4 | **-1.6** | 1116 | 11.1 | 57 | 1836 | 1543 | 29.1 | 57 | F | 5 | 021YAGKN | general transcription factor IIA, 2 (12kD subunit) | Multiple Bands | BG431140 Entrez UniGene | 2457833 | pINCY |
| 188 | 4999 | -1.3 | **-1.6** | 647 | 6.5 | 89 | 1019 | 856 | 15.5 | 89 | A | 2 | 021WAGNF | cellular retinoic acid-binding protein 1 | Passed | BG715841 Entrez UniGene | 585432 | pSport1 |
| 189 | 9885 | -1.3 | **-1.6** | 1381 | 13.9 | 91 | 2153 | 1809 | 32.7 | 91 | B | 6 | 021SAGN6 | transcriptional coactivator | Passed | AI088790 Entrez UniGene | 1842227 | pSport1 |
| 190 | 6897 | -1.3 | **-1.6** | 1433 | 11.8 | 64 | 2280 | 1916 | 27.6 | 64 | H | 5 | 021AAGMN | Homo sapiens, Similar to src homology three (SH3) and cysteine rich domain, clone MGC:2793 IMAGE:2961089, mRNA, complete cds | Passed | AW410140 Entrez UniGene | 2256026 | pSport1 |
| 191 | 2434 | -1.3 | **-1.6** | 653 | 5.1 | 64 | 1016 | 854 | 9.7 | 64 | C | 7 | 021PAGNE | KIAA0101 gene product | Passed | BG560803 Entrez UniGene | 2458926 | pINCY |
| 192 | 3039 | -1.3 | **-1.6** | 672 | 6.3 | 100 | 1066 | 896 | 12.9 | 100 | C | 6 | 0219AGL5 | SWI/SNF related, matrix associated, actin dependent regulator of chromatin, subfamily a, member 2 | Passed | R56503 Entrez UniGene | 3660322 | pINCY |
| 193 | 2824 | -1.3 | **-1.6** | 8903 | 70.6 | 89 | 14242 | 11968 | 148.8 | 89 | C | 8 | 021PAGKW | protein kinase, cAMP-dependent, regulatory, type I, alpha (tissue specific extinguisher 1) | Passed | BE257854 Entrez UniGene | 1627426 | pINCY |
| 194 | 5756 | -1.4 | **-1.6** | 1533 | 12.2 | 100 | 2513 | 2112 | 28.8 | 100 | D | 3 | 021ZAGLC | immediate early protein | Passed | BG255669 Entrez UniGene | 1217963 | pSport1 |
| 195 | 1764 | -1.3 | **-1.6** | 712 | 6.4 | 100 | 1136 | 955 | 14.1 | 100 | C | 11 | 0213AGMM | Sjogren's syndrome nuclear autoantigen 1 | Passed | BC004118 Entrez UniGene | 1710701 | pINCY |
| 196 | 2614 | -1.3 | **-1.6** | 759 | 7.8 | 66 | 1182 | 993 | 16.8 | 66 | E | 8 | 021YAGKN | complement component 1, r subcomponent | Passed | M14058 Entrez UniGene | 1664320 | pINCY |
| 197 | 373 | -1.3 | **-1.6** | 802 | 7.0 | 83 | 1288 | 1082 | 18.7 | 83 | E | 1 | 021AAGL0 | general transcription factor IIH, polypeptide 4 (52kD subunit) | Passed | AW401633 Entrez UniGene | 2470646 | pINCY |
| 198 | 1591 | -1.3 | **-1.6** | 464 | 4.1 | 70 | 734 | 617 | 8.0 | 70 | C | 1 | 021QAGMF | lymphocyte-activation gene 3 | Passed | NM\_002286 Entrez UniGene | 684538 | pSport1 |
| 199 | 9555 | -1.3 | **-1.6** | 903 | 8.1 | 100 | 1415 | 1189 | 16.6 | 100 | D | 6 | 0219AGMS | ATX1 (antioxidant protein 1, yeast) homolog 1 | No Amplification | AI276280 Entrez UniGene | 2313349 | pSport1 |
| 200 | 7470 | -1.4 | **-1.6** | 3269 | 30.2 | 93 | 5365 | 4508 | 76.1 | 93 | F | 11 | 0214AGNB | chemokine (C-C motif) receptor 7 | Passed | L08176 Entrez UniGene | 2652665 | pINCY |

---

**†** Probe 1 did not meet selection criteria

---

### Order LifeArray clones

Previous 100
Next 100

1
**2**
3
4
5
6
7
8
9
10
11
12
13
14
15
16
17
18
19
20
21
22
23
24
25
26
27
28
29
30
31
32
33
34
35
36
37
38
39
40
41
42
43
44
45
46
47
48
49
50
51
52
53
54
55
56
57
58
59
60
61
62
63
64
65
66
67
68
69
70
71
72
73
74
75
76
77
78
79
80
81
82
83
84
85
86
87
88
89
90
91
92
93
94

Entire List in plain text (long -- 1.91 MB)

|  |  |  |
| --- | --- | --- |
| PDF image of LifeArray (long -- 2.43 MB) | LifeArray color bar: |  |

To save your LifeArray™ results on your computer, use the 'Plain Text' option to display your results, then save them on your computer with your browser's save feature. We will also provide your LifeArray results on a CD-ROM for a nominal fee. Please contact our Technical Support group if you need any assistance.

We guarantee that your LifeArray results will remain on the server for 90 days after it was first uploaded. After that, we may remove and archive your LifeArray results at our discretion. Please contact our Technical Support group if you need any archived LifeArray results restored to our server.

In order to view or print Adobe® Acrobat® PDF files, you need the Adobe Acrobat Reader. If you do not already have it installed, you can obtain it for free from the Adobe web site .

If you have questions about the documents or have difficulty downloading the Acrobat Reader, please contact us.

Download the LifeArray Frequently Asked Questions list in HTML format.

Download the Human UniGEM V Frequently Asked Questions list in HTML format.

Download the LifeArray Control Plate Document in HTML format.

Adobe and Acrobat are trademarks of Adobe Systems Incorporated.

---

### Sort Again:

|  |  |
| --- | --- |
| **Username:** |  |
| **Password:** |  |
| **Sort Order:** | Ascending Descending |
| **Sort By:** | Location Diff Expr Balanced Diff Expr P1 Signal P1 S/B P2 Balanced Signal P2 Signal P2 S/B Plate ID/Row/Col Gene Name |
| **Plate ID:** |  |
| **Gene Name:** |  |

  

---

LifeArray Products  
Incyte Genomics Reagents Home
