# Supplementary material for: Trans-Activation of the Coactivator-Associated Arginine Methyltransferase 1 (Carm1) Gene by the Oncogene Product Tax of Human T-Cell Leukemia Virus Type 1
Source: Genes (Basel). 2024 May 27;15(6):698. doi: 10.3390/genes15060698 (PMC11202806; doi:10.3390/genes15060698)
Supplement: Supplementary file 1 [file genes-15-00698-s001.zip › Supplementary Table S2.pdf]

Human UniGene 1 Results

Experiment Results Generated by GEMTools 2.5
Client: Genome_Systems
Export Date: Oct 12, 2001

**GEM**	022JC38J
**Balance Coefficient**	1.19
**Minimum S/B**	2.5
**Minimum Area**	40%
**Probe 1**	123YA1BU
**P1 Description**	d17/5
**Probe 2**	1235A1BV
**P2 Description**	wt

Report sorted by **Balanced Diff Expr** in **Ascending** order
Ranks from **101** to **200**

Order LifeArray clones

Legend

Rank	Location	Diff Expr	Balanced Diff Expr	P1 Signal	P1 S/B	P1 Area %	P2 Balanced Signal	P2 Signal	P2 S/B	P2 Area %	Plate Row	Plate Col	Plate ID	Gene Name	PCR Status	GenBank Id	Clone Id (Sequence)	Vector
101	8632	-1.6	**-1.9**	371	4.5	85	703	591	10.7	85	H	8	021IAGLP	SWI/SNF related, matrix associated, actin dependent regulator of chromatin, subfamily a, member 2	Passed	X72889 Entrez UniGene	2944175	pINCY

102	3089	-1.6	**-1.9**	142	2.1	83†	263	221	3.9	83	C	10	021NAGL7	fascin (Strongylocentrotus purpuratus) homolog 2 (actin-bundling protein, retinal)	Passed	NM_012418 Entrez UniGene	4855125	pINCY
103	6438	-1.6	**-1.9**	480	5.2	100	934	785	13.9	100	F	11	0218AGM4	thymidine kinase 1, soluble	Passed	BE746447 Entrez UniGene	2055926	pSport1
104	629	-1.6	**-1.9**	496	4.4	100	933	784	12.4	100	A	9	021SAGLB	UDP-N-acetyl-alpha-D-galactosamine:polypeptide N-acetylgalactosaminyltransferase 2 (GalNAc-T2)	Passed	AL580930 Entrez UniGene	516735	pSport1
105	8186	-1.6	**-1.9**	514	5.8	98	997	838	14.7	98	D	4	021NAGL7	potassium intermediate/small conductance calcium-activated channel, subfamily N, member 4	Passed	BG741215 Entrez UniGene	2793840	pINCY
106	4503	-1.6	**-1.9**	545	5.0	67	1014	852	11.7	67	C	6	021NAGMU	lamin B2	Passed	AU130032 Entrez UniGene	2414632	pINCY
107	527	-1.6	**-1.9**	859	7.0	100	1626	1366	19.0	100	G	9	021GAGL6	tumor rejection antigen (gp96) 1	Passed	AV655628 Entrez UniGene	3624631	pINCY

108	5144	-1.6	**-1.9**	974	6.2	44	1833	1540	14.9	44	H	3	021RAGKM	protein disulfide isomerase related protein (calcium-binding protein, intestinal-related)	Passed	NM_004911 Entrez UniGene	1824957	pINCY
109	63	-1.6	**-1.9**	1283	10.1	65	2395	2013	31.0	65	E	5	021YAGKN	FXYD domain-containing ion transport regulator 2	Passed	BG166035 Entrez UniGene	2380042	pINCY
110	7557	-1.6	**-1.9**	1387	12.0	65	2658	2234	34.8	65	D	5	021WAGNF	malate dehydrogenase 1, NAD (soluble)	Passed	NM_005917 Entrez UniGene	487539	pBlue
111	104	-1.6	**-1.9**	2197	13.1	55	4075	3424	35.9	55	C	3	021CAGKP	KIAA0084 protein	Passed	AL561563 Entrez UniGene	2697959	pINCY
112	415	-1.6	**-1.9**	3427	27.0	62	6562	5514	87.2	62	C	1	021OAGL2	RNA-binding protein regulatory subunit	Passed	BG715880 Entrez UniGene	1879921	pINCY
113	7301	-1.6	**-1.9**	227	2.4	79†	433	364	5.5	79	F	9	021EAGN4	hypothetical protein KIAA1165	No Amplification	R82692 Entrez UniGene	55889	pBlue

114	3622	-1.5	**-1.8**	152	2.2	100†	273	229	4.3	100	E	8	021AAGLT	arginine-rich, mutated in early stage tumors	Passed	AA974308 Entrez UniGene	1618455	pINCY
115	3787	-1.5	**-1.8**	153	2.1	92†	269	226	4.0	92	E	2	021GAGM0	KIAA0974 protein	Passed	BE669938 Entrez UniGene	4540	pBlue
116	3017	-1.5	**-1.8**	173	2.2	86†	307	258	4.4	86	C	10	0212AGL4	platelet activating receptor homolog	Passed	NM_013308 Entrez UniGene	3879095	pINCY
117	6495	-1.5	**-1.8**	154	2.1	86†	276	232	4.1	86	B	5	021TAGM7	Homo sapiens cDNA: FLJ21897 fis, clone HEP03447, highly similar to AF052178 Homo sapiens clone 24523 mRNA sequence	Passed	BE259271 Entrez UniGene	2518964	pINCY
118	6374	-1.5	**-1.8**	1481	14.2	100	2600	2185	36.2	100	B	3	021UAGM2	FYN-binding protein (FYB-120/130)	Passed	AF198052 Entrez UniGene	1614872	pINCY
119	9411	-1.5	**-1.8**	268	2.9	88	471	396	5.7	88	D	6	0213AGMM	synaptophysin-like protein	Passed	S72481 Entrez UniGene	1798283	pINCY

120	7570	-1.5	**-1.8**	287	3.3	82	512	430	7.6	82	H	7	021WAGNF	S-adenosylhomocysteine hydrolase-like 1	Passed	AL527927 Entrez UniGene	1739627	pSport1
121	7577	-1.5	**-1.8**	297	3.2	70	528	444	7.6	70	B	9	0213AGNG	CD58 antigen, (lymphocyte function-associated antigen 3)	Passed	NM_001779 Entrez UniGene	342285	pBlue
122	1322	-1.5	**-1.8**	212	2.5	96	383	322	6.0	96	A	3	0218AGM4	nuclear matrix protein p84	Passed	AV713026 Entrez UniGene	22715	pBlue
123	6786	-1.5	**-1.8**	2578	22.8	55	4618	3881	56.4	55	B	11	021IAGMJ	tubulin, beta polypeptide	Passed	BG762520 Entrez UniGene	3334367	pINCY
124	2716	-1.5	**-1.8**	1160	10.8	66	2048	1721	28.3	66	G	8	021QAGKR	diazepam binding inhibitor (GABA receptor modulator, acyl-Coenzyme A binding protein)	Passed	AA843364 Entrez UniGene	2060396	pSport1
125	4316	-1.5	**-1.8**	1114	9.6	100	2040	1714	23.9	100	E	4	0213AGMM	transporter 1, ATP-binding cassette, sub-family B (MDR/TAP)	Passed	NM_000593 Entrez UniGene	1634279	pINCY

126	488	-1.5	**-1.8**	641	5.7	68	1157	972	12.9	68	C	3	0219AGL5	TNF receptor-associated factor 2	Passed	BG677004 Entrez UniGene	3226017	pINCY
127	1926	-1.5	**-1.8**	369	4.2	85	655	550	10.6	85	A	11	021GAGMT	leptin (murine obesity homolog)	Passed	NM_000230 Entrez UniGene	1961822	pSport1
128	1273	-1.5	**-1.8**	387	3.9	93	679	571	10.2	93	A	1	021UAGM2	grancalcin, EF-hand calcium-binding protein	Passed	BG545676 Entrez UniGene	1671852	pINCY
129	1177	-1.5	**-1.8**	406	4.1	85	735	618	10.1	85	A	1	0219AGLY	early growth response 1	Passed	AL553329 Entrez UniGene	1705208	pINCY
130	5138	-1.5	**-1.8**	453	3.7	62	815	685	8.4	62	F	3	021RAGKM	ribosomal protein S6 kinase, 90kD, polypeptide 1	Passed	NM_002953 Entrez UniGene	1822236	pINCY
131	8359	-1.5	**-1.8**	462	4.3	88	833	700	9.2	88	F	2	021DAGLE	protein kinase, cAMP-dependent, regulatory, type I, beta	Passed	BF940317 Entrez UniGene	1926688	pSport1
132	7799	-1.5	**-1.8**	519	4.5	50	925	777	10.4	50	B	10	021QAGKR	spermine synthase	Passed	BG535109 Entrez UniGene	2061432	pSport1

133	6370	-1.5	**-1.8**	1054	10.6	100	1853	1557	27.0	100	H	7	021NAGM1	SEC14 (S. cerevisiae)-like 1	Passed	NM_003003 Entrez UniGene	1879956	pINCY
134	6529	-1.5	**-1.8**	540	5.6	100	988	830	14.2	100	F	1	0210AGM8	solute carrier family 29 (nucleoside transporters), member 1	Passed	BI088219 Entrez UniGene	2594080	pINCY
135	9461	-1.5	**-1.8**	135	2.1	88†	244	205	3.8	88	D	10	021HAGMO	cathepsin H	Passed	BC002479 Entrez UniGene	1749417	pINCY
136	4361	-1.5	**-1.8**	125	1.9	83†	223	187	3.4	83	C	10	021HAGMO	nuclear receptor subfamily 4, group A, member 1	Passed	NM_002135 Entrez UniGene	1958560	pINCY
137	6375	-1.5	**-1.8**	921	8.7	100	1695	1424	23.3	100	B	5	021UAGM2	v-rel avian reticuloendotheliosis viral oncogene homolog B (nuclear factor of kappa light polypeptide gene enhancer in B-cells 3)	Passed	BG748809 Entrez UniGene	1859449	pINCY
138	10180	-1.6	**-1.8**	845	9.0	62	1561	1312	24.9	62	D	8	021MAKNL	Control: Sensitivity 2000pg				
139	3168	-1.5	**-1.8**	300	3.0	87	546	459	6.4	87	E	12	021LAGLA	DNA segment on chromosome X (unique) 9879 expressed sequence	Passed	AI360458 Entrez UniGene	2585358	pINCY

140	3805	-1.5	**-1.8**	624	6.4	66	1147	964	17.1	66	C	2	021NAGM1	tetracycline transporter-like protein	Passed	L11669 Entrez UniGene	1856947	pINCY
141	1	-1.5	**-1.8**	547	3.5	55	1004	844	10.3	55	A	1	0216AKON	Internal_Control_E				
142	8522	-1.5	**-1.8**	109	2.0	97†	198	166	3.5	97	D	4	021QAGLL	hypothetical protein FLJ23231	Passed	AA640102 Entrez UniGene	2348706	pINCY
143	6701	-1.5	**-1.7**	1063	7.7	65	1858	1561	20.3	65	F	9	021QAGMF	SH2 domain protein 2A	Passed	NM_003975 Entrez UniGene	504273	pBlue
144	7630	-1.5	**-1.7**	1477	14.0	61	2567	2157	36.4	61	D	7	021MAKNL	Control: Sensitivity 2000pg				
145	7083	-1.4	**-1.7**	2448	23.3	86	4100	3445	54.0	86	F	5	021UAGMV	tumor necrosis factor receptor superfamily, member 9	Passed	BG436824 Entrez UniGene	191843	pBlue
146	1102	-1.4	**-1.7**	895	7.1	100	1482	1245	18.8	100	G	7	021HAGLU	nuclear factor of kappa light polypeptide gene enhancer in B-cells inhibitor, epsilon	Passed	BG469491 Entrez UniGene	2748942	pINCY
147	10047	-1.4	**-1.7**	910	8.5	62	1509	1268	20.2	62	H	6	021BAGNC	pericentrin 2 (kendrin)	Passed	AB007862 Entrez UniGene	1655365	pINCY

148	9493	-1.5	**-1.7**	6971	60.6	49	12108	10175	155.2	49	H	2	021OAGMP	x 006 protein	Passed	BF445491 Entrez UniGene	440017	pBlue
149	8042	-1.4	**-1.7**	19343	175.3	51	32332	27170	412.7	51	D	4	021HAGL1	nuclear receptor subfamily 1, group H, member 2	Passed	BE878950 Entrez UniGene	2581075	pINCY
150	3741	-1.4	**-1.7**	909	8.2	100	1509	1268	18.6	100	E	6	0219AGLY	tumor necrosis factor receptor superfamily, member 14 (herpesvirus entry mediator)	Multiple Bands	BC002794 Entrez UniGene	2121653	pINCY
151	9008	-1.4	**-1.7**	2975	26.9	82	4985	4189	66.5	82	F	4	021FAGM5	peroxiredoxin 1	Passed	BG612736 Entrez UniGene	3942594	pINCY
152	9528	-1.4	**-1.7**	345	4.2	92	595	500	9.3	92	B	12	0212AGMR	metallothionein 1L	Passed	BG928106 Entrez UniGene	2513883	pINCY
153	1089	-1.4	**-1.7**	170	2.1	59†	292	245	3.8	59	C	5	021HAGLU	solute carrier family 8 (sodium/calcium exchanger), member 1	Passed	NM_021097 Entrez UniGene	2880435	pINCY
154	6796	-1.4	**-1.7**	844	7.4	62	1448	1217	17.4	62	F	7	021IAGMJ	cytochrome b-245, alpha polypeptide	Passed	BG751568 Entrez UniGene	4173205	pINCY

155	2932	-1.5	**-1.7**	757	7.3	100	1315	1105	16.8	100	G	8	021AAGL0	macrophage myristoylated alanine-rich C kinase substrate	Passed	AL534743 Entrez UniGene	2135733	pINCY
156	4287	-1.4	**-1.7**	718	7.4	100	1225	1029	17.1	100	C	6	021WAGML	tubulin, beta, 5	Passed	AL536237 Entrez UniGene	1486358	pINCY
157	1774	-1.4	**-1.7**	554	4.7	100	938	788	10.7	100	G	7	0213AGMM	growth arrest and DNA-damage-inducible, alpha	Passed	AI935984 Entrez UniGene	1702350	pINCY
158	5390	-1.4	**-1.7**	432	4.3	75	745	626	10.2	75	B	3	021WAGKX	modulator recognition factor I	Passed	M62324 Entrez UniGene	1568724	pINCY
159	117	-1.4	**-1.7**	3127	24.0	53	5178	4351	65.0	53	G	5	021CAGKP	LIM domain kinase 1	Passed	NM_002314 Entrez UniGene	3373632	pINCY
160	8372	-1.4	**-1.7**	368	4.1	92	616	518	8.8	92	B	4	021KAGLF	keratin 7	Passed	BI094014 Entrez UniGene	1962141	pSport1

161	6631	-1.4	**-1.7**	319	3.7	93	528	444	8.4	93	H	1	0215AGMC	uridine monophosphate synthetase (orotate phosphoribosyl transferase and orotidine-5'-decarboxylase)	Passed	AU13306 5 Entrez UniGene	1816355	pINCY
162	1887	-1.4	**-1.7**	295	3.0	73	502	422	6.4	73	E	5	0212AGMR	replication protein A3 (14kD)	Passed	BF030135 Entrez UniGene	2451279	pINCY
163	8993	-1.4	**-1.7**	264	3.3	87	449	377	7.7	87	H	10	0218AGM4	KIAA0005 gene product	Passed	NM_014670 Entrez UniGene	2059208	pSport1
164	9799	-1.4	**-1.7**	259	2.8	68	430	361	5.8	68	F	2	0210AGN2	methylenetetrahydrofolate dehydrogenase (NADP+ dependent), methenyltetrahydrofolate cyclohydrolase, formyltetrahydrofolate synthetase	Passed	BC001014 Entrez UniGene	1297179	pINCY
165	8262	-1.4	**-1.7**	226	2.7	82	383	322	5.4	82	D	12	021LAGLA	neural polypyrimidine tract binding protein	Passed	BG991819 Entrez UniGene	2578983	pINCY
166	8033	-1.5	**-1.7**	199	2.6	90	346	291	4.9	90	H	10	021AAGL0	retinoid X receptor, alpha	Passed	X52773 Entrez UniGene	2808269	pINCY

167	4534	-1.5	**-1.7**	151	1.9	76†	261	219	3.5	76	E	8	021UAGMV	E2F transcription factor 1	Passed	AL121906 Entrez UniGene	1920739	pSport1
168	5991	-1.4	**-1.7**	148	2.1	72†	253	213	3.7	72	B	5	021XAGLM	HTPAP protein	Passed	AI765129 Entrez UniGene	2626340	pINCY
169	3731	-1.4	**-1.6**	174	2.5	98	282	237	5.0	98	A	10	0219AGLY	annexin A8	Passed	BC008813 Entrez UniGene	1911622	pINCY
170	3280	-1.4	**-1.6**	165	2.4	92†	269	226	4.2	92	C	8	021KAGLF	RNA helicase-related protein	Passed	BF679543 Entrez UniGene	2757583	pSport1
171	6994	-1.3	**-1.6**	230	2.5	75	361	303	4.8	75	H	7	0212AGMR	vav 2 oncogene	Passed	AL045952 Entrez UniGene	3744592	pINCY
172	9458	-1.3	**-1.6**	280	3.1	90	445	374	5.7	90	D	4	021HAGMO	serine/threonine kinase 4	Passed	NM_006282 Entrez UniGene	1510581	pINCY
173	2358	-1.4	**-1.6**	164	2.0	65†	268	225	3.4	65	A	11	0214AGNB	RAB9, member RAS oncogene family	Passed	BG535930 Entrez UniGene	1746329	pINCY

174	7474	-1.4	**-1.6**	335	3.9	86	543	456	8.5	86	H	7	0214AGNB	aldo-keto reductase family 1, member A1 (aldehyde reductase)	Passed	AW873466 Entrez UniGene	1634342	pINCY
175	2349	-1.3	**-1.6**	174	2.1	59†	274	230	3.8	59	G	5	021XAGNA	biliverdin reductase A	Passed	AI765830 Entrez UniGene	775330	pSport1
176	725	-1.4	**-1.6**	875	7.6	100	1443	1213	19.4	100	A	9	021KAGLF	chemokine (C-C motif) receptor 6	Passed	AL121935 Entrez UniGene	3190228	pSport1
177	7445	-1.3	**-1.6**	162	2.1	59†	256	215	3.4	59	F	9	021XAGNA	thymopoietin	Passed	BF983366 Entrez UniGene	56919	pBlue
178	4665	-1.4	**-1.6**	480	5.1	66	775	651	9.8	66	A	6	021TAGN1	insulin-like growth factor 2 (somatomedin A)	Passed	BG619049 Entrez UniGene	2955178	pINCY
179	1549	-1.3	**-1.6**	496	4.2	58	771	648	7.8	58	E	1	021CAGMD	ADP-ribosylation factor 4	Passed	BG529241 Entrez UniGene	65463	pBlue
180	6748	-1.3	**-1.6**	130	2.1	75†	207	174	3.5	75	F	7	0214AGMH	exonuclease 1	Passed	AC004783 Entrez UniGene	4385292	pINCY

181	7700	-1.4	**-1.6**	1003	7.7	58	1642	1380	19.4	58	B	4	021YAGKN	tumor necrosis factor (ligand) superfamily, member 7	Passed	BG170786 Entrez UniGene	2017463	pINCY
182	4678	-1.3	**-1.6**	528	5.5	78	833	700	11.0	78	E	8	021TAGN1	proteasome (prosome, macropain) subunit, beta type, 6	Passed	BF205294 Entrez UniGene	2989852	pINCY
183	4526	-1.4	**-1.6**	437	4.4	72	709	596	10.5	72	C	4	021UAGMV	benzodiazapine receptor (peripheral)	Passed	BE531172 Entrez UniGene	1241680	pSport1
184	4806	-1.3	**-1.6**	214	2.3	56†	342	287	4.2	56	G	12	021SAGN6	cAMP responsive element modulator	Passed	AL117336 Entrez UniGene	1921290	pSport1
185	2828	-1.4	**-1.6**	537	5.4	100	872	733	12.1	100	E	4	021PAGKW	intracellular hyaluronan-binding protein	Passed	AK025144 Entrez UniGene	1755234	pINCY
186	9466	-1.3	**-1.6**	601	6.5	100	933	784	13.4	100	F	8	021HAGMO	proteasome (prosome, macropain) subunit, alpha type, 4	Passed	BG178069 Entrez UniGene	1975642	pINCY
187	5163	-1.4	**-1.6**	1116	11.1	57	1836	1543	29.1	57	F	5	021YAGKN	general transcription factor IIA, 2 (12kD subunit)	Multiple Bands	BG431140 Entrez UniGene	2457833	pINCY

188	4999	-1.3	**-1.6**	647	6.5	89	1019	856	15.5	89	A	2	021WAGNF	cellular retinoic acid-binding protein 1	Passed	BG71584 1 Entrez UniGene	585432	pSport1
189	9885	-1.3	**-1.6**	1381	13.9	91	2153	1809	32.7	91	B	6	021SAGN6	transcriptional coactivator	Passed	AI088790 Entrez UniGene	1842227	pSport1
190	6897	-1.3	**-1.6**	1433	11.8	64	2280	1916	27.6	64	H	5	021AAGMN	Homo sapiens, Similar to src homology three (SH3) and cysteine rich domain, clone MGC:2793 IMAGE:2961089, mRNA, complete cds	Passed	AW41014 0 Entrez UniGene	2256026	pSport1
191	2434	-1.3	**-1.6**	653	5.1	64	1016	854	9.7	64	C	7	021PAGNE	KIAA0101 gene product	Passed	BG56080 3 Entrez UniGene	2458926	pINCY
192	3039	-1.3	**-1.6**	672	6.3	100	1066	896	12.9	100	C	6	0219AGL5	SWI/SNF related, matrix associated, actin dependent regulator of chromatin, subfamily a, member 2	Passed	R56503 Entrez UniGene	3660322	pINCY
193	2824	-1.3	**-1.6**	8903	70.6	89	14242	11968	148.8	89	C	8	021PAGKW	protein kinase, cAMP-dependent, regulatory, type I, alpha (tissue specific extinguisher 1)	Passed	BE25785 4 Entrez UniGene	1627426	pINCY

194	5756	-1.4	**-1.6**	1533	12.2	100	2513	2112	28.8	100	D	3	021ZAGLC	immediate early protein	Passed	BG255669 Entrez UniGene	1217963	pSport1
195	1764	-1.3	**-1.6**	712	6.4	100	1136	955	14.1	100	C	11	0213AGMM	Sjogren's syndrome nuclear autoantigen 1	Passed	BC004118 Entrez UniGene	1710701	pINCY
196	2614	-1.3	**-1.6**	759	7.8	66	1182	993	16.8	66	E	8	021YAGKN	complement component 1, r subcomponent	Passed	M14058 Entrez UniGene	1664320	pINCY
197	373	-1.3	**-1.6**	802	7.0	83	1288	1082	18.7	83	E	1	021AAGL0	general transcription factor IIH, polypeptide 4 (52kD subunit)	Passed	AW401633 Entrez UniGene	2470646	pINCY
198	1591	-1.3	**-1.6**	464	4.1	70	734	617	8.0	70	C	1	021QAGMF	lymphocyte-activation gene 3	Passed	NM_002286 Entrez UniGene	684538	pSport1
199	9555	-1.3	**-1.6**	903	8.1	100	1415	1189	16.6	100	D	6	0219AGMS	ATX1 (antioxidant protein 1, yeast) homolog 1	No Amplification	AI276280 Entrez UniGene	2313349	pSport1
200	7470	-1.4	**-1.6**	3269	30.2	93	5365	4508	76.1	93	F	11	0214AGNB	chemokine (C-C motif) receptor 7	Passed	L08176 Entrez UniGene	2652665	pINCY

† Probe 1 did not meet selection criteria

Order LifeArray clones

Previous 100 Next 100

1 2 3 4 5 6 7 8 9 10 11 12 13 14 15 16 17 18 19 20 21 22 23 24 25 26 27 28 29 30 31 32 33 34 35 36 37 38 39 40 41 42 43 44 45 46 47 48 49 50 51 52 53 54 55 56 57 58 59 60 61 62 63 64 65 66 67 68 69 70 71 72 73 74 75 76 77 78 79 80 81 82 83 84 85 86 87 88 89 90 91 92 93 94

Entire List in plain text (long -- 1.91 MB)

PDF image of LifeArray (long -- 2.43 MB)	LifeArray color bar:

To save your LifeArray™ results on your computer, use the 'Plain Text' option to display your results, then save them on your computer with your browser's save feature. We will also provide your LifeArray results on a CD-ROM for a nominal fee. Please contact our Technical Support group if you need any assistance.

We guarantee that your LifeArray results will remain on the server for 90 days after it was first uploaded. After that, we may remove and archive your LifeArray results at our discretion. Please contact our Technical Support group if you need any archived LifeArray results restored to our server.

In order to view or print Adobe® Acrobat® PDF files, you need the Adobe Acrobat Reader. If you do not already have it installed, you can obtain it for free from the Adobe web site .

If you have questions about the documents or have difficulty downloading the Acrobat Reader, please contact us.

Download the LifeArray Frequently Asked Questions list in HTML format.

Download the Human UniGEM V Frequently Asked Questions list in HTML format.

Download the LifeArray Control Plate Document in HTML format.

Adobe and Acrobat are trademarks of Adobe Systems Incorporated.

Sort Again:

Username: nature
Password:
Sort Order: Ascending Descending

**Sort By:**	Location<br>Diff Expr<br>Balanced Diff Expr<br>P1 Signal<br>P1 S/B<br>P2 Balanced Signal<br>P2 Signal<br>P2 S/B<br>Plate ID/Row/Col<br>Gene Name
**Plate ID:**	
**Gene Name:**	
