# Supplementary material for: Trans-Activation of the Coactivator-Associated Arginine Methyltransferase 1 (Carm1) Gene by the Oncogene Product Tax of Human T-Cell Leukemia Virus Type 1
Source: Genes (Basel). 2024 May 27;15(6):698. doi: 10.3390/genes15060698 (PMC11202806; doi:10.3390/genes15060698)
Supplement: Supplementary file 1 [file genes-15-00698-s001.zip › Supplementary Table S3]

Human UniGene 1 Results

## Human UniGene 1 Results

Experiment Results Generated by GEMTools 2.5  
Client: Genome\_Systems  
Export Date: Oct 12, 2001  


---

|  |  |
| --- | --- |
| GEM | 022JC38J |
| Balance Coefficient | 1.19 |
| Minimum S/B | 2.5 |
| Minimum Area | 40% |
| Probe 1 | 123YA1BU |
| P1 Description | d17/5 |
| Probe 2 | 1235A1BV |
| P2 Description | wt |

---

Report sorted by **Balanced Diff Expr** in **Ascending** order  
Ranks from **201** to **300**

### Order LifeArray clones

Legend

| Rank | Location | Diff Expr | Balanced Diff Expr | P1 Signal | P1 S/B | P1 Area % | P2 Balanced Signal | P2 Signal | P2 S/B | P2 Area % | Plate Row | Plate Col | Plate ID | Gene Name | PCR Status | GenBank Id | Clone Id (Sequence) | Vector |
| --- | --- | --- | --- | --- | --- | --- | --- | --- | --- | --- | --- | --- | --- | --- | --- | --- | --- | --- |
| 201 | 4691 | -1.3 | **-1.6** | 1605 | 13.3 | 61 | 2499 | 2100 | 28.3 | 61 | A | 10 | 0210AGN2 | tropomyosin 2 (beta) | Passed | AA283746 Entrez UniGene | 3176845 | pINCY |
| 202 | 3208 | -1.4 | **-1.6** | 1977 | 14.1 | 53 | 3262 | 2741 | 29.3 | 53 | C | 8 | 021ZAGLC | minichromosome maintenance deficient (S. cerevisiae) 7 | Passed | BG683250 Entrez UniGene | 986752 | pSport1 |
| 203 | 4196 | -1.3 | **-1.6** | 2088 | 16.8 | 50 | 3243 | 2725 | 39.9 | 50 | E | 4 | 0214AGMH | neuropeptide Y | Passed | K01911 Entrez UniGene | 2494284 | pINCY |
| 204 | 7009 | -1.3 | **-1.6** | 3116 | 25.3 | 86 | 4963 | 4171 | 59.7 | 86 | F | 1 | 0219AGMS | CD44 antigen (homing function and Indian blood group system) | Passed | BG822701 Entrez UniGene | 549196 | pSport1 |
| 205 | 542 | -1.3 | **-1.6** | 2439 | 17.9 | 50 | 3901 | 3278 | 40.5 | 50 | E | 3 | 021NAGL7 | FK506-binding protein 1A (12kD) | Passed | AL539248 Entrez UniGene | 2204204 | pINCY |
| 206 | 4094 | -1.3 | **-1.6** | 2284 | 18.8 | 93 | 3628 | 3049 | 42.2 | 93 | C | 4 | 021CAGMD | dual specificity phosphatase 4 | Passed | NM\_001394 Entrez UniGene | 740878 | pSport1 |
| 207 | 6084 | -1.4 | **-1.6** | 126 | 2.0 | 86**†** | 205 | 172 | 3.2 | 86 | H | 11 | 021IAGLP | sperm associated antigen 1 | Passed | NM\_003114 Entrez UniGene | 2962332 | pINCY |
| 208 | 4080 | -1.3 | **-1.5** | 192 | 2.5 | 93 | 287 | 241 | 4.4 | 93 | E | 12 | 0215AGMC | uridine phosphorylase | Passed | BG492119 Entrez UniGene | 1806435 | pINCY |
| 209 | 2448 | -1.3 | **-1.5** | 118 | 1.9 | 65**†** | 179 | 150 | 2.8 | 65 | G | 11 | 021PAGNE | KIAA0222 gene product | Passed | AW510659 Entrez UniGene | 1527755 | pINCY |
| 210 | 1535 | -1.2 | **-1.5** | 240 | 2.5 | 74 | 355 | 298 | 4.8 | 74 | G | 9 | 0215AGMC | cytochrome P450, subfamily IIC (mephenytoin 4-hydroxylase), polypeptide 18 | Passed | M61853 Entrez UniGene | 2595728 | pINCY |
| 211 | 289 | -1.3 | **-1.5** | 256 | 2.6 | 71 | 383 | 322 | 5.4 | 71 | A | 1 | 021WAGKX | glutamine-fructose-6-phosphate transaminase 2 | Passed | AK001242 Entrez UniGene | 1904696 | pINCY |
| 212 | 679 | -1.2 | **-1.5** | 259 | 2.8 | 88 | 383 | 322 | 5.5 | 88 | C | 1 | 0216AGLD | B-cell CLL/lymphoma 6 (zinc finger protein 51) | Passed | NM\_001706 Entrez UniGene | 1920672 | pSport1 |
| 213 | 8351 | -1.2 | **-1.5** | 288 | 3.0 | 73 | 426 | 358 | 5.7 | 73 | B | 10 | 021DAGLE | B-cell CLL/lymphoma 1 | Passed | Z23022 Entrez UniGene | 1994739 | pSport1 |
| 214 | 2949 | -1.2 | **-1.5** | 290 | 3.1 | 77 | 424 | 356 | 5.9 | 77 | E | 6 | 021HAGL1 | paraoxonase 2 | Passed | BG699928 Entrez UniGene | 2134968 | pINCY |
| 215 | 2492 | -1.3 | **-1.5** | 295 | 3.1 | 58 | 439 | 369 | 5.4 | 58 | G | 3 | 0213AGNG | Incyte EST | Passed |  | 87242 | pBlue |
| 216 | 9552 | -1.3 | **-1.5** | 300 | 3.3 | 81 | 462 | 388 | 6.1 | 81 | B | 12 | 0219AGMS | gamma-glutamyl hydrolase (conjugase, folylpolygammaglutamyl hydrolase) | Passed | BG717516 Entrez UniGene | 1997967 | pSport1 |
| 217 | 7716 | -1.2 | **-1.5** | 301 | 3.3 | 73 | 441 | 371 | 6.9 | 73 | F | 12 | 021YAGKN | aldo-keto reductase family 1, member B1 (aldose reductase) | Passed | AV703256 Entrez UniGene | 1901073 | pINCY |
| 218 | 2495 | -1.3 | **-1.5** | 327 | 3.1 | 49 | 496 | 417 | 5.3 | 49 | G | 9 | 0213AGNG | ubiquitin carrier protein | Passed | AI571293 Entrez UniGene | 2057823 | pSport1 |
| 219 | 5872 | -1.3 | **-1.5** | 334 | 3.8 | 85 | 516 | 434 | 8.0 | 85 | B | 7 | 021YAGLH | CDK2-associated protein 1 | Passed | NM\_004642 Entrez UniGene | 902381 | pSport1 |
| 220 | 1044 | -1.2 | **-1.5** | 347 | 3.2 | 71 | 505 | 424 | 5.9 | 71 | C | 11 | 0213AGLS | early growth response 3 | Passed | NM\_004430 Entrez UniGene | 2633001 | pINCY |
| 221 | 2540 | -1.3 | **-1.5** | 360 | 3.8 | 70 | 537 | 451 | 7.8 | 70 | G | 3 | 021MAKNL | Control: Complex Target (Homo sapiens) |  |  |  |  |
| 222 | 5682 | -1.3 | **-1.5** | 380 | 4.1 | 76 | 577 | 485 | 8.6 | 76 | B | 11 | 0211AGL9 | calcium and integrin binding protein (DNA-dependent protein kinase interacting protein) | Passed | BF685744 Entrez UniGene | 4626895 | pINCY |
| 223 | 2082 | -1.2 | **-1.5** | 394 | 4.2 | 82 | 578 | 486 | 8.8 | 82 | E | 11 | 021MAGMZ | ZW10 interactor | Passed | AW409765 Entrez UniGene | 1576329 | pINCY |
| 224 | 10178 | -1.3 | **-1.5** | 430 | 5.2 | 96 | 644 | 541 | 10.3 | 96 | D | 4 | 021MAKNL | Control: Sensitivity 20pg |  |  |  |  |
| 225 | 455 | -1.3 | **-1.5** | 436 | 4.2 | 75 | 657 | 552 | 9.5 | 75 | G | 9 | 021VAGL3 | myotubularin related protein 4 | No Amplification | BG012919 Entrez UniGene | 2890336 | pINCY |
| 226 | 10179 | -1.3 | **-1.5** | 438 | 5.1 | 91 | 652 | 548 | 10.4 | 91 | D | 6 | 021MAKNL | Control: Sensitivity 200pg |  |  |  |  |
| 227 | 3097 | -1.3 | **-1.5** | 489 | 4.8 | 68 | 732 | 615 | 10.5 | 68 | G | 2 | 021NAGL7 | agrin | Passed | AF016903 Entrez UniGene | 4610962 | pINCY |
| 228 | 9607 | -1.3 | **-1.5** | 506 | 4.6 | 72 | 763 | 641 | 8.1 | 72 | F | 2 | 021NAGMU | replication protein A2 (32kD) | Passed | BG333934 Entrez UniGene | 1729876 | pINCY |
| 229 | 6538 | -1.2 | **-1.5** | 537 | 5.5 | 97 | 784 | 659 | 10.8 | 97 | H | 7 | 0210AGM8 | KIAA0399 protein | Passed | AB007859 Entrez UniGene | 2347845 | pINCY |
| 230 | 2529 | -1.2 | **-1.5** | 568 | 5.3 | 84 | 844 | 709 | 11.6 | 84 | C | 5 | 021MAKNL | Control: Sensitivity 200pg |  |  |  |  |
| 231 | 6166 | -1.3 | **-1.5** | 613 | 6.0 | 100 | 939 | 789 | 13.0 | 100 | D | 7 | 021AAGLT | cell division cycle 2, G1 to S and G2 to M | Passed | BF968871 Entrez UniGene | 1525795 | pINCY |
| 232 | 1046 | -1.2 | **-1.5** | 661 | 6.0 | 71 | 979 | 823 | 12.0 | 71 | E | 3 | 0213AGLS | glucosidase I | Passed | NM\_006302 Entrez UniGene | 1555701 | pINCY |
| 233 | 2346 | -1.3 | **-1.5** | 688 | 5.2 | 58 | 1038 | 872 | 10.7 | 58 | E | 11 | 021XAGNA | HS1 binding protein | Passed | AL523082 Entrez UniGene | 155194 | pBlue |
| 234 | 4669 | -1.3 | **-1.5** | 743 | 6.9 | 65 | 1109 | 932 | 12.2 | 65 | C | 2 | 021TAGN1 | pim-1 oncogene | Passed | NM\_002648 Entrez UniGene | 2679117 | pINCY |
| 235 | 7892 | -1.3 | **-1.5** | 746 | 5.8 | 58 | 1135 | 954 | 11.4 | 58 | B | 4 | 021IAGKV | decidual protein induced by progesterone | Passed | AK002191 Entrez UniGene | 1960889 | pSport1 |
| 236 | 341 | -1.2 | **-1.5** | 773 | 7.0 | 77 | 1128 | 948 | 14.0 | 77 | A | 9 | 021AAGKZ | ras homolog gene family, member | Passed | AI167227 Entrez UniGene | 1910324 | pINCY |
| 237 | 6355 | -1.3 | **-1.5** | 779 | 7.0 | 100 | 1180 | 992 | 16.3 | 100 | D | 1 | 021NAGM1 | interleukin 4 receptor | Multiple Bands | NM\_000418 Entrez UniGene | 1808529 | pINCY |
| 238 | 4633 | -1.3 | **-1.5** | 799 | 8.9 | 67 | 1201 | 1009 | 17.5 | 67 | G | 2 | 021MAGMZ | SWI/SNF related, matrix associated, actin dependent regulator of chromatin, subfamily d, member 1 | Multiple Bands | BC009368 Entrez UniGene | 1658083 | pINCY |
| 239 | 7021 | -1.3 | **-1.5** | 898 | 8.2 | 100 | 1382 | 1161 | 17.1 | 100 | B | 1 | 021GAGMT | protein translocation complex beta | Passed | BG029603 Entrez UniGene | 1966933 | pSport1 |
| 240 | 8845 | -1.3 | **-1.5** | 952 | 9.4 | 65 | 1454 | 1222 | 21.7 | 65 | H | 2 | 0219AGLY | small nuclear ribonucleoprotein polypeptide G | Passed | AV762663 Entrez UniGene | 2449837 | pINCY |
| 241 | 2530 | -1.3 | **-1.5** | 975 | 8.7 | 68 | 1454 | 1222 | 19.8 | 68 | C | 7 | 021MAKNL | Control: Sensitivity 2000pg |  |  |  |  |
| 242 | 582 | -1.3 | **-1.5** | 978 | 8.2 | 100 | 1468 | 1234 | 16.2 | 100 | A | 11 | 0211AGL9 | hypocretin (orexin) neuropeptide precursor | Passed | BG194545 Entrez UniGene | 4588486 | pINCY |
| 243 | 1092 | -1.3 | **-1.5** | 1020 | 7.9 | 62 | 1568 | 1318 | 17.9 | 62 | C | 11 | 021HAGLU | autoimmune regulator (automimmune polyendocrinopathy candidiasis ectodermal dystrophy) | Passed | AJ009610 Entrez UniGene | 2914128 | pINCY |
| 244 | 5028 | -1.2 | **-1.5** | 1056 | 10.1 | 67 | 1566 | 1316 | 20.4 | 67 | A | 12 | 0213AGNG | acyl-Coenzyme A oxidase 2, branched chain | Passed | BG545036 Entrez UniGene | 943744 | pSport1 |
| 245 | 6619 | -1.2 | **-1.5** | 1081 | 9.3 | 100 | 1595 | 1340 | 19.1 | 100 | D | 1 | 0215AGMC | activity-regulated cytoskeleton-associated protein | Passed | AF193421 Entrez UniGene | 1382579 | pINCY |
| 246 | 3638 | -1.2 | **-1.5** | 1135 | 9.6 | 100 | 1654 | 1390 | 20.6 | 100 | C | 4 | 021HAGLU | phosphorylase, glycogen; muscle (McArdle syndrome, glycogen storage disease type V) | Passed | NM\_005609 Entrez UniGene | 2635943 | pINCY |
| 247 | 10107 | -1.2 | **-1.5** | 1204 | 9.0 | 62 | 1752 | 1472 | 16.8 | 62 | D | 6 | 021WAGNF | Nef-associated factor 1 | Passed | NM\_006058 Entrez UniGene | 1985586 | pSport1 |
| 248 | 792 | -1.3 | **-1.5** | 1212 | 8.7 | 59 | 1830 | 1538 | 18.3 | 59 | G | 11 | 021YAGLH | interferon-induced, hepatitis C-associated microtubular aggregate protein (44kD) | Passed | NM\_006417 Entrez UniGene | 1922658 | pSport1 |
| 249 | 2963 | -1.2 | **-1.5** | 1216 | 9.9 | 100 | 1799 | 1512 | 20.6 | 100 | A | 10 | 021OAGL2 | thyroid hormone receptor interactor 10 | Passed | NM\_004240 Entrez UniGene | 2814551 | pINCY |
| 250 | 5597 | -1.2 | **-1.5** | 1382 | 12.4 | 59 | 2043 | 1717 | 30.1 | 59 | F | 9 | 0219AGL5 | thyroid hormone receptor interactor 10 | Passed | NM\_004240 Entrez UniGene | 1655192 | pINCY |
| 251 | 3646 | -1.2 | **-1.5** | 1389 | 11.8 | 72 | 2043 | 1717 | 25.2 | 72 | E | 8 | 021HAGLU | non-metastatic cells 3, protein expressed in | Passed | BC000250 Entrez UniGene | 2455170 | pINCY |
| 252 | 2069 | -1.3 | **-1.5** | 1439 | 13.1 | 65 | 2150 | 1807 | 31.1 | 65 | A | 9 | 021MAGMZ | cell membrane glycoprotein, 110000M(r) (surface antigen) | Passed | BF033463 Entrez UniGene | 1720946 | pINCY |
| 253 | 5122 | -1.2 | **-1.5** | 1459 | 10.5 | 51 | 2134 | 1793 | 24.6 | 51 | H | 7 | 0216AKON | Internal\_Control\_Z |  |  |  |  |
| 254 | 6682 | -1.2 | **-1.5** | 1459 | 12.6 | 100 | 2123 | 1784 | 28.0 | 100 | H | 7 | 021JAGME | minichromosome maintenance deficient (S. cerevisiae) 5 (cell division cycle 46) | Passed | AU149507 Entrez UniGene | 1746529 | pINCY |
| 255 | 72 | -1.2 | **-1.5** | 1573 | 12.2 | 75 | 2284 | 1919 | 25.3 | 75 | G | 11 | 021YAGKN | ferredoxin reductase | Passed | NM\_004110 Entrez UniGene | 1819763 | pINCY |
| 256 | 2355 | -1.2 | **-1.5** | 1725 | 12.3 | 62 | 2541 | 2135 | 27.4 | 62 | A | 5 | 0214AGNB | HMT1 (hnRNP methyltransferase, S. cerevisiae)-like 2 | Passed | BG167159 Entrez UniGene | 2888814 | pINCY |
| 257 | 9137 | -1.3 | **-1.5** | 1744 | 17.0 | 100 | 2616 | 2198 | 35.3 | 100 | H | 10 | 021RAGMA | calcium channel, voltage-dependent, gamma subunit 1 | Passed | NM\_000727 Entrez UniGene | 4014318 | pINCY |
| 258 | 5782 | -1.3 | **-1.5** | 1871 | 15.7 | 65 | 2783 | 2339 | 34.9 | 65 | D | 7 | 0216AGLD | cyclin D1 (PRAD1: parathyroid adenomatosis 1) | Passed | X59798 Entrez UniGene | 2057653 | pSport1 |
| 259 | 3808 | -1.3 | **-1.5** | 1938 | 17.3 | 62 | 2936 | 2467 | 33.5 | 62 | C | 8 | 021NAGM1 | cathepsin C | Passed | NM\_001814 Entrez UniGene | 1822716 | pINCY |
| 260 | 1034 | -1.3 | **-1.5** | 1939 | 18.3 | 62 | 2992 | 2514 | 43.6 | 62 | A | 3 | 0213AGLS | ras homolog gene family, member G (rho G) | Passed | BG338917 Entrez UniGene | 1342744 | pINCY |
| 261 | 5600 | -1.3 | **-1.5** | 1996 | 14.6 | 45 | 3052 | 2565 | 31.9 | 45 | H | 3 | 0219AGL5 | delta-like homolog (Drosophila) | Passed | BF969929 Entrez UniGene | 3288394 | pINCY |
| 262 | 7388 | -1.3 | **-1.5** | 2222 | 18.6 | 49 | 3397 | 2855 | 38.6 | 49 | D | 3 | 0216AGN8 | protein tyrosine phosphatase, receptor type, U | Passed | AL049570 Entrez UniGene | 2941607 | pINCY |
| 263 | 2862 | -1.3 | **-1.5** | 2523 | 21.5 | 59 | 3763 | 3162 | 46.8 | 59 | G | 12 | 021WAGKX | selenophosphate synthetase 2 | Passed | BG674956 Entrez UniGene | 1687542 | pINCY |
| 264 | 7993 | -1.2 | **-1.5** | 2648 | 24.9 | 47 | 3892 | 3271 | 54.6 | 47 | D | 2 | 021AAGKZ | amphiregulin (schwannoma-derived growth factor) | Passed | AL546917 Entrez UniGene | 2350594 | pINCY |
| 265 | 2486 | -1.3 | **-1.5** | 3108 | 28.3 | 56 | 4658 | 3914 | 64.1 | 56 | E | 3 | 0213AGNG | neurogranin (protein kinase C substrate, RC3) | Multiple Bands | AW117600 Entrez UniGene | 1943863 | pBlue |
| 266 | 7741 | -1.3 | **-1.5** | 3676 | 29.5 | 47 | 5535 | 4651 | 59.1 | 47 | H | 2 | 0215AGKO | hypothetical protein FLJ20030 | Passed | AI589003 Entrez UniGene | 3027978 | pINCY |
| 267 | 2009 | -1.2 | **-1.5** | 4770 | 37.1 | 98 | 7003 | 5885 | 86.3 | 98 | E | 9 | 0211AGMW | calreticulin | Passed | BG675100 Entrez UniGene | 2970280 | pINCY |
| 268 | 113 | -1.2 | **-1.5** | 4897 | 30.3 | 62 | 7233 | 6078 | 68.5 | 62 | E | 9 | 021CAGKP | double C2-like domains, alpha | Passed | BE646046 Entrez UniGene | 1797982 | pINCY |
| 269 | 3041 | -1.3 | **-1.5** | 5768 | 47.9 | 59 | 8870 | 7454 | 104.5 | 59 | C | 10 | 0219AGL5 | calcitonin/calcitonin-related polypeptide, alpha | Passed | X03662 Entrez UniGene | 2498725 | pINCY |
| 270 | 4860 | -1.2 | **-1.5** | 151 | 2.1 | 79**†** | 223 | 187 | 3.3 | 79 | A | 12 | 021DAGN9 | SH3-domain binding protein 1 | Passed | AL157480 Entrez UniGene | 2132470 | pSport1 |
| 271 | 89 | -1.1 | **-1.4** | 30400 | 202.3 | 50 | 41185 | 34609 | 444.7 | 50 | E | 9 | 0215AGKO | actin, gamma 1 | Passed | BG422944 Entrez UniGene | 3225977 | pINCY |
| 272 | 1030 | -1.2 | **-1.4** | 217 | 2.6 | 95 | 306 | 257 | 5.0 | 95 | G | 7 | 021WAGLR | hypothetical protein MGC2495 | Passed | AA972572 Entrez UniGene | 958633 | pSport1 |
| 273 | 4504 | -1.2 | **-1.4** | 7057 | 47.7 | 48 | 10036 | 8434 | 90.7 | 48 | C | 8 | 021NAGMU | lysosomal-associated membrane protein 2 | Passed | BG719002 Entrez UniGene | 2503017 | pINCY |
| 274 | 9289 | -1.1 | **-1.4** | 6364 | 49.2 | 52 | 8644 | 7264 | 104.8 | 52 | D | 2 | 0214AGMH | major histocompatibility complex, class I, B | Multiple Bands | BG754309 Entrez UniGene | 2859033 | pINCY |
| 275 | 3018 | -1.2 | **-1.4** | 4521 | 34.2 | 92 | 6248 | 5250 | 63.5 | 92 | C | 12 | 0212AGL4 | vinexin beta (SH3-containing adaptor molecule-1) | Passed | BG252405 Entrez UniGene | 1957809 | pINCY |
| 276 | 28 | -1.2 | **-1.4** | 3815 | 22.7 | 49 | 5424 | 4558 | 50.0 | 49 | A | 7 | 021RAGKM | tubulin, alpha 1 (testis specific) | Passed | BE742772 Entrez UniGene | 1872936 | pINCY |
| 277 | 8014 | -1.2 | **-1.4** | 3581 | 32.4 | 52 | 5042 | 4237 | 67.2 | 52 | B | 8 | 021AAGL0 | mitogen-activated protein kinase kinase kinase 11 | Passed | NM\_002419 Entrez UniGene | 1992626 | pINCY |
| 278 | 1689 | -1.1 | **-1.4** | 3433 | 24.0 | 49 | 4676 | 3929 | 51.4 | 49 | C | 5 | 021IAGMJ | tyrosine 3-monooxygenase/tryptophan 5-monooxygenase activation protein, theta polypeptide | Passed | NM\_006826 Entrez UniGene | 4169223 | pINCY |
| 279 | 2989 | -1.1 | **-1.4** | 3360 | 29.0 | 89 | 4595 | 3861 | 60.4 | 89 | C | 2 | 021VAGL3 | Homo sapiens clone 23608 mRNA sequence | Passed | BC008032 Entrez UniGene | 2642865 | pINCY |
| 280 | 7756 | -1.2 | **-1.4** | 2910 | 25.7 | 45 | 4130 | 3471 | 53.6 | 45 | D | 8 | 021CAGKP | KIAA0544 protein | Passed | BF801696 Entrez UniGene | 4174315 | pINCY |
| 281 | 69 | -1.2 | **-1.4** | 2745 | 19.4 | 52 | 3826 | 3215 | 40.2 | 52 | G | 5 | 021YAGKN | splicing factor, arginine/serine-rich 3 | Passed | BG287081 Entrez UniGene | 2474214 | pINCY |
| 282 | 9609 | -1.2 | **-1.4** | 2629 | 23.7 | 49 | 3739 | 3142 | 45.9 | 49 | F | 6 | 021NAGMU | ets variant gene 2 | Passed | AF000671 Entrez UniGene | 1320685 | pINCY |
| 283 | 7037 | -1.1 | **-1.4** | 2590 | 22.8 | 64 | 3525 | 2962 | 48.0 | 64 | F | 9 | 021GAGMT | RAN, member RAS oncogene family | Passed | BG111605 Entrez UniGene | 552654 | pSport1 |
| 284 | 8324 | -1.1 | **-1.4** | 2533 | 24.7 | 58 | 3452 | 2901 | 46.3 | 58 | B | 4 | 0216AGLD | vesicle-associated membrane protein 3 (cellubrevin) | Passed | BG401423 Entrez UniGene | 1988078 | pSport1 |
| 285 | 393 | -1.1 | **-1.4** | 2244 | 13.7 | 44 | 3062 | 2573 | 25.7 | 44 | C | 5 | 021HAGL1 | ATPase, H+ transporting, lysosomal (vacuolar proton pump), member D | Passed | BF311825 Entrez UniGene | 2923189 | pINCY |
| 286 | 7629 | -1.1 | **-1.4** | 2022 | 17.2 | 44 | 2755 | 2315 | 34.1 | 44 | D | 5 | 021MAKNL | Control: Sensitivity 200pg |  |  |  |  |
| 287 | 2636 | -1.2 | **-1.4** | 2002 | 15.9 | 49 | 2741 | 2303 | 29.8 | 49 | E | 4 | 0215AGKO | H2B histone family, member Q | Passed | BF794197 Entrez UniGene | 3040858 | pINCY |
| 288 | 3822 | -1.1 | **-1.4** | 210 | 2.8 | 97 | 287 | 241 | 4.6 | 97 | G | 12 | 021NAGM1 | M-phase phosphoprotein 6 | Passed | BI088194 Entrez UniGene | 1806540 | pINCY |
| 289 | 2121 | -1.2 | **-1.4** | 1879 | 14.0 | 64 | 2628 | 2208 | 29.7 | 64 | C | 5 | 021TAGN1 | SH3-domain GRB2-like 1 | Passed | BE563866 Entrez UniGene | 2346978 | pINCY |
| 290 | 2835 | -1.2 | **-1.4** | 1589 | 13.7 | 70 | 2225 | 1870 | 28.9 | 70 | G | 6 | 021PAGKW | plexin B2 | Passed | BC004542 Entrez UniGene | 1831287 | pINCY |
| 291 | 903 | -1.2 | **-1.4** | 1430 | 10.2 | 93 | 2028 | 1704 | 24.7 | 93 | E | 5 | 021XAGLM | COBW-like protein | Passed | BG709004 Entrez UniGene | 2220048 | pINCY |
| 292 | 2153 | -1.2 | **-1.4** | 1401 | 10.5 | 67 | 2004 | 1684 | 25.1 | 67 | E | 9 | 0210AGN2 | glutathione peroxidase 1 | Passed | BG764734 Entrez UniGene | 3137033 | pINCY |
| 293 | 4983 | -1.1 | **-1.4** | 1350 | 12.2 | 62 | 1846 | 1551 | 23.5 | 62 | C | 6 | 021PAGNE | splicing factor, arginine/serine-rich (transformer 2 Drosophila homolog) 10 | Passed | AL560128 Entrez UniGene | 2457759 | pINCY |
| 294 | 2298 | -1.2 | **-1.4** | 1343 | 8.9 | 48 | 1868 | 1570 | 18.4 | 48 | E | 11 | 0216AGN8 | Finkel-Biskis-Reilly murine sarcoma virus (FBR-MuSV) ubiquitously expressed (fox derived); ribosomal protein S30 | Passed | AA316067 Entrez UniGene | 2734906 | pINCY |
| 295 | 1532 | -1.2 | **-1.4** | 1275 | 12.9 | 73 | 1756 | 1476 | 26.4 | 73 | G | 3 | 0215AGMC | Homo sapiens, clone MGC:18203 IMAGE:4155840, mRNA, complete cds | Passed | AW248283 Entrez UniGene | 2135596 | pINCY |
| 296 | 1866 | -1.2 | **-1.4** | 1264 | 10.2 | 100 | 1743 | 1465 | 23.2 | 100 | E | 11 | 021VAGMQ | keratin 7 | Passed | AA307373 Entrez UniGene | 1649959 | pINCY |
| 297 | 2641 | -1.1 | **-1.4** | 1239 | 10.4 | 100 | 1677 | 1409 | 20.3 | 100 | G | 2 | 0215AGKO | crystallin, alpha B | Passed | BF727296 Entrez UniGene | 3016305 | pINCY |
| 298 | 1348 | -1.2 | **-1.4** | 1185 | 9.5 | 66 | 1627 | 1367 | 20.0 | 66 | A | 7 | 021FAGM5 | albumin | Passed | N24732 Entrez UniGene | 3354436 | pINCY |
| 299 | 1555 | -1.2 | **-1.4** | 1157 | 9.2 | 100 | 1584 | 1331 | 19.7 | 100 | G | 1 | 021CAGMD | serine/threonine kinase 12 | Passed | BG616089 Entrez UniGene | 161207 | pBlue |
| 300 | 7956 | -1.2 | **-1.4** | 177 | 2.3 | 82**†** | 245 | 206 | 3.5 | 82 | F | 12 | 021WAGKX | era (E. coli G-protein homolog)-like 1 | Passed | BE871890 Entrez UniGene | 1686892 | pINCY |

---

**†** Probe 1 did not meet selection criteria

---

### Order LifeArray clones

Previous 100
Next 100

1
2
**3**
4
5
6
7
8
9
10
11
12
13
14
15
16
17
18
19
20
21
22
23
24
25
26
27
28
29
30
31
32
33
34
35
36
37
38
39
40
41
42
43
44
45
46
47
48
49
50
51
52
53
54
55
56
57
58
59
60
61
62
63
64
65
66
67
68
69
70
71
72
73
74
75
76
77
78
79
80
81
82
83
84
85
86
87
88
89
90
91
92
93
94

Entire List in plain text (long -- 1.91 MB)

|  |  |  |
| --- | --- | --- |
| PDF image of LifeArray (long -- 2.43 MB) | LifeArray color bar: |  |

To save your LifeArray™ results on your computer, use the 'Plain Text' option to display your results, then save them on your computer with your browser's save feature. We will also provide your LifeArray results on a CD-ROM for a nominal fee. Please contact our Technical Support group if you need any assistance.

We guarantee that your LifeArray results will remain on the server for 90 days after it was first uploaded. After that, we may remove and archive your LifeArray results at our discretion. Please contact our Technical Support group if you need any archived LifeArray results restored to our server.

In order to view or print Adobe® Acrobat® PDF files, you need the Adobe Acrobat Reader. If you do not already have it installed, you can obtain it for free from the Adobe web site .

If you have questions about the documents or have difficulty downloading the Acrobat Reader, please contact us.

Download the LifeArray Frequently Asked Questions list in HTML format.

Download the Human UniGEM V Frequently Asked Questions list in HTML format.

Download the LifeArray Control Plate Document in HTML format.

Adobe and Acrobat are trademarks of Adobe Systems Incorporated.

---

### Sort Again:

|  |  |
| --- | --- |
| **Username:** |  |
| **Password:** |  |
| **Sort Order:** | Ascending Descending |
| **Sort By:** | Location Diff Expr Balanced Diff Expr P1 Signal P1 S/B P2 Balanced Signal P2 Signal P2 S/B Plate ID/Row/Col Gene Name |
| **Plate ID:** |  |
| **Gene Name:** |  |

  

---

LifeArray Products  
Incyte Genomics Reagents Home
