# Supplementary material for: Trans-Activation of the Coactivator-Associated Arginine Methyltransferase 1 (Carm1) Gene by the Oncogene Product Tax of Human T-Cell Leukemia Virus Type 1
Source: Genes (Basel). 2024 May 27;15(6):698. doi: 10.3390/genes15060698 (PMC11202806; doi:10.3390/genes15060698)
Supplement: Supplementary file 1 [file genes-15-00698-s001.zip › Supplementary Table S3.pdf]

Human UniGene 1 Results

Experiment Results Generated by GEMTools 2.5
Client: Genome_Systems
Export Date: Oct 12, 2001

**GEM**	022JC38J
**Balance Coefficient**	1.19
**Minimum S/B**	2.5
**Minimum Area**	40%
**Probe 1**	123YA1BU
**P1 Description**	d17/5
**Probe 2**	1235A1BV
**P2 Description**	wt

Report sorted by **Balanced Diff Expr** in **Ascending** order
Ranks from **201** to **300**

Order LifeArray clones

Legend

Rank	Location	Diff Expr	Balanced Diff Expr	P1 Signal	P1 S/B	P1 Area %	P2 Balanced Signal	P2 Signal	P2 S/B	P2 Area %	Plate Row	Plate Col	Plate ID	Gene Name	PCR Status	GenBank Id	Clone Id (Sequence)	Vector
201	4691	-1.3	**-1.6**	1605	13.3	61	2499	2100	28.3	61	A	10	0210AGN2	tropomyosin 2 (beta)	Passed	AA283746 Entrez UniGene	3176845	pINCY
202	3208	-1.4	**-1.6**	1977	14.1	53	3262	2741	29.3	53	C	8	021ZAGLC	minichromosome maintenance deficient (S. cerevisiae) 7	Passed	BG683250 Entrez UniGene	986752	pSport1

203	4196	-1.3	**-1.6**	2088	16.8	50	3243	2725	39.9	50	E	4	0214AGMH	neuropeptide Y	Passed	K01911 Entrez UniGene	2494284	pINCY
204	7009	-1.3	**-1.6**	3116	25.3	86	4963	4171	59.7	86	F	1	0219AGMS	CD44 antigen (homing function and Indian blood group system)	Passed	BG822701 Entrez UniGene	549196	pSport1
205	542	-1.3	**-1.6**	2439	17.9	50	3901	3278	40.5	50	E	3	021NAGL7	FK506-binding protein 1A (12kD)	Passed	AL539248 Entrez UniGene	2204204	pINCY
206	4094	-1.3	**-1.6**	2284	18.8	93	3628	3049	42.2	93	C	4	021CAGMD	dual specificity phosphatase 4	Passed	NM_001394 Entrez UniGene	740878	pSport1
207	6084	-1.4	**-1.6**	126	2.0	86†	205	172	3.2	86	H	11	021IAGLP	sperm associated antigen 1	Passed	NM_003114 Entrez UniGene	2962332	pINCY
208	4080	-1.3	**-1.5**	192	2.5	93	287	241	4.4	93	E	12	0215AGMC	uridine phosphorylase	Passed	BG492119 Entrez UniGene	1806435	pINCY
209	2448	-1.3	**-1.5**	118	1.9	65†	179	150	2.8	65	G	11	021PAGNE	KIAA0222 gene product	Passed	AW510659 Entrez UniGene	1527755	pINCY

210	1535	-1.2	**-1.5**	240	2.5	74	355	298	4.8	74	G	9	0215AGMC	cytochrome P450, subfamily IIC (mephenytoin 4-hydroxylase), polypeptide 18	Passed	M61853 Entrez UniGene	2595728	pINCY
211	289	-1.3	**-1.5**	256	2.6	71	383	322	5.4	71	A	1	021WAGKX	glutamine-fructose-6-phosphate transaminase 2	Passed	AK001242 Entrez UniGene	1904696	pINCY
212	679	-1.2	**-1.5**	259	2.8	88	383	322	5.5	88	C	1	0216AGLD	B-cell CLL/lymphoma 6 (zinc finger protein 51)	Passed	NM_001706 Entrez UniGene	1920672	pSport1
213	8351	-1.2	**-1.5**	288	3.0	73	426	358	5.7	73	B	10	021DAGLE	B-cell CLL/lymphoma 1	Passed	Z23022 Entrez UniGene	1994739	pSport1
214	2949	-1.2	**-1.5**	290	3.1	77	424	356	5.9	77	E	6	021HAGL1	paraoxonase 2	Passed	BG699928 Entrez UniGene	2134968	pINCY
215	2492	-1.3	**-1.5**	295	3.1	58	439	369	5.4	58	G	3	0213AGNG	Incyte EST	Passed		87242	pBlue
216	9552	-1.3	**-1.5**	300	3.3	81	462	388	6.1	81	B	12	0219AGMS	gamma-glutamyl hydrolase (conjugase, folylpolygammaglutamyl hydrolase)	Passed	BG717516 Entrez UniGene	1997967	pSport1

217	7716	-1.2	**-1.5**	301	3.3	73	441	371	6.9	73	F	12	021YAGKN	aldo-keto reductase family 1, member B1 (aldose reductase)	Passed	AV70325 6 Entrez UniGene	1901073	pINCY
218	2495	-1.3	**-1.5**	327	3.1	49	496	417	5.3	49	G	9	0213AGNG	ubiquitin carrier protein	Passed	AI571293 Entrez UniGene	2057823	pSport1
219	5872	-1.3	**-1.5**	334	3.8	85	516	434	8.0	85	B	7	021YAGLH	CDK2-associated protein 1	Passed	NM_004642 Entrez UniGene	902381	pSport1
220	1044	-1.2	**-1.5**	347	3.2	71	505	424	5.9	71	C	11	0213AGLS	early growth response 3	Passed	NM_004430 Entrez UniGene	2633001	pINCY
221	2540	-1.3	**-1.5**	360	3.8	70	537	451	7.8	70	G	3	021MAKNL	Control: Complex Target (Homo sapiens)				
222	5682	-1.3	**-1.5**	380	4.1	76	577	485	8.6	76	B	11	0211AGL9	calcium and integrin binding protein (DNA-dependent protein kinase interacting protein)	Passed	BF685744 Entrez UniGene	4626895	pINCY

223	2082	-1.2	**-1.5**	394	4.2	82	578	486	8.8	82	E	11	021MAGMZ	ZW10 interactor	Passed	AW40976 5 Entrez UniGene	1576329	pI NCY
224	10178	-1.3	**-1.5**	430	5.2	96	644	541	10.3	96	D	4	021MAKNL	Control: Sensitivity 20pg				
225	455	-1.3	**-1.5**	436	4.2	75	657	552	9.5	75	G	9	021VAGL3	myotubularin related protein 4	No Amplification	BG01291 9 Entrez UniGene	2890336	pI NCY
226	10179	-1.3	**-1.5**	438	5.1	91	652	548	10.4	91	D	6	021MAKNL	Control: Sensitivity 200pg				
227	3097	-1.3	**-1.5**	489	4.8	68	732	615	10.5	68	G	2	021NAGL7	agrin	Passed	AF016903 Entrez UniGene	4610962	pI NCY
228	9607	-1.3	**-1.5**	506	4.6	72	763	641	8.1	72	F	2	021NAGMU	replication protein A2 (32kD)	Passed	BG33393 4 Entrez UniGene	1729876	pI NCY
229	6538	-1.2	**-1.5**	537	5.5	97	784	659	10.8	97	H	7	0210AGM8	KIAA0399 protein	Passed	AB07859 Entrez UniGene	2347845	pI NCY
230	2529	-1.2	**-1.5**	568	5.3	84	844	709	11.6	84	C	5	021MAKNL	Control: Sensitivity 200pg				

231	6166	-1.3	**-1.5**	613	6.0	100	939	789	13.0	100	D	7	021AAGLT	cell division cycle 2, G1 to S and G2 to M	Passed	BF968871 Entrez UniGene	1525795	pINCY
232	1046	-1.2	**-1.5**	661	6.0	71	979	823	12.0	71	E	3	0213AGLS	glucosidase I	Passed	NM_006302 Entrez UniGene	1555701	pINCY
233	2346	-1.3	**-1.5**	688	5.2	58	1038	872	10.7	58	E	11	021XAGNA	HS1 binding protein	Passed	AL523082 Entrez UniGene	155194	pBlue
234	4669	-1.3	**-1.5**	743	6.9	65	1109	932	12.2	65	C	2	021TAGN1	pim-1 oncogene	Passed	NM_002648 Entrez UniGene	2679117	pINCY
235	7892	-1.3	**-1.5**	746	5.8	58	1135	954	11.4	58	B	4	021IAGKV	decidual protein induced by progesterone	Passed	AK002191 Entrez UniGene	1960889	pSport1
236	341	-1.2	**-1.5**	773	7.0	77	1128	948	14.0	77	A	9	021AAGKZ	ras homolog gene family, member	Passed	AI167227 Entrez UniGene	1910324	pINCY
237	6355	-1.3	**-1.5**	779	7.0	100	1180	992	16.3	100	D	1	021NAGM1	interleukin 4 receptor	Multiple Bands	NM_000418 Entrez UniGene	1808529	pINCY

238	4633	-1.3	**-1.5**	799	8.9	67	1201	1009	17.5	67	G	2	021MAGMZ	SWI/SNF related, matrix associated, actin dependent regulator of chromatin, subfamily d, member 1	Multiple Bands	BC009368 Entrez UniGene	1658083	pINCY
239	7021	-1.3	**-1.5**	898	8.2	100	1382	1161	17.1	100	B	1	021GAGMT	protein translocation complex beta	Passed	BG029603 Entrez UniGene	1966933	pSport1
240	8845	-1.3	**-1.5**	952	9.4	65	1454	1222	21.7	65	H	2	0219AGLY	small nuclear ribonucleoprotein polypeptide G	Passed	AV762663 Entrez UniGene	2449837	pINCY
241	2530	-1.3	**-1.5**	975	8.7	68	1454	1222	19.8	68	C	7	021MAKNL	Control: Sensitivity 2000pg				
242	582	-1.3	**-1.5**	978	8.2	100	1468	1234	16.2	100	A	11	0211AGL9	hypocretin (orexin) neuropeptide precursor	Passed	BG194545 Entrez UniGene	4588486	pINCY
243	1092	-1.3	**-1.5**	1020	7.9	62	1568	1318	17.9	62	C	11	021HAGLU	autoimmune regulator (automimmune polyendocrinopathy candidiasis ectodermal dystrophy)	Passed	AJ009610 Entrez UniGene	2914128	pINCY

244	5028	-1.2	**-1.5**	1056	10.1	67	1566	1316	20.4	67	A	12	0213AGNG	acyl-Coenzyme A oxidase 2, branched chain	Passed	BG54503 6 Entrez UniGene	943744	pSport1
245	6619	-1.2	**-1.5**	1081	9.3	100	1595	1340	19.1	100	D	1	0215AGMC	activity-regulated cytoskeleton-associated protein	Passed	AF193421 Entrez UniGene	1382579	pINCY
246	3638	-1.2	**-1.5**	1135	9.6	100	1654	1390	20.6	100	C	4	021HAGLU	phosphorylase, glycogen; muscle (McArdle syndrome, glycogen storage disease type V)	Passed	NM_005609 Entrez UniGene	2635943	pINCY
247	10107	-1.2	**-1.5**	1204	9.0	62	1752	1472	16.8	62	D	6	021WAGNF	Nef-associated factor 1	Passed	NM_006058 Entrez UniGene	1985586	pSport1
248	792	-1.3	**-1.5**	1212	8.7	59	1830	1538	18.3	59	G	11	021YAGLH	interferon-induced, hepatitis C-associated microtubular aggregate protein (44kD)	Passed	NM_006417 Entrez UniGene	1922658	pSport1
249	2963	-1.2	**-1.5**	1216	9.9	100	1799	1512	20.6	100	A	10	021OAGL2	thyroid hormone receptor interactor 10	Passed	NM_004240 Entrez UniGene	2814551	pINCY

250	5597	-1.2	**-1.5**	1382	12.4	59	2043	1717	30.1	59	F	9	0219AGL5	thyroid hormone receptor interactor 10	Passed	NM_004240 Entrez UniGene	1655192	pI NCY
251	3646	-1.2	**-1.5**	1389	11.8	72	2043	1717	25.2	72	E	8	021HAGLU	non-metastatic cells 3, protein expressed in	Passed	BC00250 Entrez UniGene	2455170	pI NCY
252	2069	-1.3	**-1.5**	1439	13.1	65	2150	1807	31.1	65	A	9	021MAGMZ	cell membrane glycoprotein, 110000M(r) (surface antigen)	Passed	BF033463 Entrez UniGene	1720946	pI NCY
253	5122	-1.2	**-1.5**	1459	10.5	51	2134	1793	24.6	51	H	7	0216AKON	Internal_Control_Z				
254	6682	-1.2	**-1.5**	1459	12.6	100	2123	1784	28.0	100	H	7	021JAGME	minichromosome maintenance deficient (S. cerevisiae) 5 (cell division cycle 46)	Passed	AU149507 Entrez UniGene	1746529	pI NCY
255	72	-1.2	**-1.5**	1573	12.2	75	2284	1919	25.3	75	G	11	021YAGKN	ferredoxin reductase	Passed	NM_004110 Entrez UniGene	1819763	pI NCY
256	2355	-1.2	**-1.5**	1725	12.3	62	2541	2135	27.4	62	A	5	0214AGNB	HMT1 (hnRNP methyltransferase, S. cerevisiae)-like 2	Passed	BG167159 Entrez UniGene	2888814	pI NCY

257	9137	-1.3	**-1.5**	1744	17.0	100	2616	2198	35.3	100	H	10	021RAGMA	calcium channel, voltage-dependent, gamma subunit 1	Passed	NM_000727 Entrez UniGene	4014318	pINCY
258	5782	-1.3	**-1.5**	1871	15.7	65	2783	2339	34.9	65	D	7	0216AGLD	cyclin D1 (PRAD1: parathyroid adenomatosis 1)	Passed	X59798 Entrez UniGene	2057653	pSport1
259	3808	-1.3	**-1.5**	1938	17.3	62	2936	2467	33.5	62	C	8	021NAGM1	cathepsin C	Passed	NM_001814 Entrez UniGene	1822716	pINCY
260	1034	-1.3	**-1.5**	1939	18.3	62	2992	2514	43.6	62	A	3	0213AGLS	ras homolog gene family, member G (rho G)	Passed	BG338917 Entrez UniGene	1342744	pINCY
261	5600	-1.3	**-1.5**	1996	14.6	45	3052	2565	31.9	45	H	3	0219AGL5	delta-like homolog (Drosophila)	Passed	BF969929 Entrez UniGene	3288394	pINCY
262	7388	-1.3	**-1.5**	2222	18.6	49	3397	2855	38.6	49	D	3	0216AGN8	protein tyrosine phosphatase, receptor type, U	Passed	AL049570 Entrez UniGene	2941607	pINCY
263	2862	-1.3	**-1.5**	2523	21.5	59	3763	3162	46.8	59	G	12	021WAGKX	selenophosphate synthetase 2	Passed	BG674956 Entrez UniGene	1687542	pINCY

264	7993	-1.2	**-1.5**	2648	24.9	47	3892	3271	54.6	47	D	2	021AAGKZ	amphiregulin (schwannoma-derived growth factor)	Passed	AL546917 Entrez UniGene	2350594	pINCY
265	2486	-1.3	**-1.5**	3108	28.3	56	4658	3914	64.1	56	E	3	0213AGNG	neurogranin (protein kinase C substrate, RC3)	Multiple Bands	AW117600 Entrez UniGene	1943863	pBlue
266	7741	-1.3	**-1.5**	3676	29.5	47	5535	4651	59.1	47	H	2	0215AGKO	hypothetical protein FLJ20030	Passed	AI589003 Entrez UniGene	3027978	pINCY
267	2009	-1.2	**-1.5**	4770	37.1	98	7003	5885	86.3	98	E	9	0211AGMW	calreticulin	Passed	BG675100 Entrez UniGene	2970280	pINCY
268	113	-1.2	**-1.5**	4897	30.3	62	7233	6078	68.5	62	E	9	021CAGKP	double C2-like domains, alpha	Passed	BE646046 Entrez UniGene	1797982	pINCY
269	3041	-1.3	**-1.5**	5768	47.9	59	8870	7454	104.5	59	C	10	0219AGL5	calcitonin/calcitonin-related polypeptide, alpha	Passed	X03662 Entrez UniGene	2498725	pINCY
270	4860	-1.2	**-1.5**	151	2.1	79†	223	187	3.3	79	A	12	021DAGN9	SH3-domain binding protein 1	Passed	AL157480 Entrez UniGene	2132470	pSport1

271	89	-1.1	**-1.4**	30400	202.3	50	41185	34609	444.7	50	E	9	0215AGKO	actin, gamma 1	Passed	BG422944 Entrez UniGene	3225977	pINCY
272	1030	-1.2	**-1.4**	217	2.6	95	306	257	5.0	95	G	7	021WAGLR	hypothetical protein MGC2495	Passed	AA972572 Entrez UniGene	958633	pSport1
273	4504	-1.2	**-1.4**	7057	47.7	48	10036	8434	90.7	48	C	8	021NAGMU	lysosomal-associated membrane protein 2	Passed	BG719002 Entrez UniGene	2503017	pINCY
274	9289	-1.1	**-1.4**	6364	49.2	52	8644	7264	104.8	52	D	2	0214AGMH	major histocompatibility complex, class I, B	Multiple Bands	BG754309 Entrez UniGene	2859033	pINCY
275	3018	-1.2	**-1.4**	4521	34.2	92	6248	5250	63.5	92	C	12	0212AGL4	vinexin beta (SH3-containing adaptor molecule-1)	Passed	BG252405 Entrez UniGene	1957809	pINCY
276	28	-1.2	**-1.4**	3815	22.7	49	5424	4558	50.0	49	A	7	021RAGKM	tubulin, alpha 1 (testis specific)	Passed	BE742772 Entrez UniGene	1872936	pINCY
277	8014	-1.2	**-1.4**	3581	32.4	52	5042	4237	67.2	52	B	8	021AAGL0	mitogen-activated protein kinase kinase kinase 11	Passed	NM_002419 Entrez UniGene	1992626	pINCY

278	1689	-1.1	**-1.4**	3433	24.0	49	4676	3929	51.4	49	C	5	021IAGMJ	tyrosine 3-monooxygenase/tryptophan 5-monooxygenase activation protein, theta polypeptide	Passed	NM_006826 Entrez UniGene	4169223	pINCY
279	2989	-1.1	**-1.4**	3360	29.0	89	4595	3861	60.4	89	C	2	021VAGL3	Homo sapiens clone 23608 mRNA sequence	Passed	BC008032 Entrez UniGene	2642865	pINCY
280	7756	-1.2	**-1.4**	2910	25.7	45	4130	3471	53.6	45	D	8	021CAGKP	KIAA0544 protein	Passed	BF801696 Entrez UniGene	4174315	pINCY
281	69	-1.2	**-1.4**	2745	19.4	52	3826	3215	40.2	52	G	5	021YAGKN	splicing factor, arginine/serine-rich 3	Passed	BG287081 Entrez UniGene	2474214	pINCY
282	9609	-1.2	**-1.4**	2629	23.7	49	3739	3142	45.9	49	F	6	021NAGMU	ets variant gene 2	Passed	AF000671 Entrez UniGene	1320685	pINCY
283	7037	-1.1	**-1.4**	2590	22.8	64	3525	2962	48.0	64	F	9	021GAGMT	RAN, member RAS oncogene family	Passed	BG111605 Entrez UniGene	552654	pSport1

284	8324	-1.1	**-1.4**	2533	24.7	58	3452	2901	46.3	58	B	4	0216AGLD	vesicle-associated membrane protein 3 (cellubrevin)	Passed	BG40142 3 Entrez UniGene	1988078	pSport1
285	393	-1.1	**-1.4**	2244	13.7	44	3062	2573	25.7	44	C	5	021HAGL1	ATPase, H+ transporting, lysosomal (vacuolar proton pump), member D	Passed	BF311825 Entrez UniGene	2923189	pINCY
286	7629	-1.1	**-1.4**	2022	17.2	44	2755	2315	34.1	44	D	5	021MAKNL	Control: Sensitivity 200pg				
287	2636	-1.2	**-1.4**	2002	15.9	49	2741	2303	29.8	49	E	4	0215AGKO	H2B histone family, member Q	Passed	BF794197 Entrez UniGene	3040858	pINCY
288	3822	-1.1	**-1.4**	210	2.8	97	287	241	4.6	97	G	12	021NAGM1	M-phase phosphoprotein 6	Passed	BI088194 Entrez UniGene	1806540	pINCY
289	2121	-1.2	**-1.4**	1879	14.0	64	2628	2208	29.7	64	C	5	021TAGN1	SH3-domain GRB2-like 1	Passed	BE563866 Entrez UniGene	2346978	pINCY
290	2835	-1.2	**-1.4**	1589	13.7	70	2225	1870	28.9	70	G	6	021PAGKW	plexin B2	Passed	BC004542 Entrez UniGene	1831287	pINCY

291	903	-1.2	**-1.4**	1430	10.2	93	2028	1704	24.7	93	E	5	021XAGLM	COBW-like protein	Passed	BG70900 4 Entrez UniGene	2220048	pI NCY
292	2153	-1.2	**-1.4**	1401	10.5	67	2004	1684	25.1	67	E	9	0210AGN2	glutathione peroxidase 1	Passed	BG76473 4 Entrez UniGene	3137033	pI NCY
293	4983	-1.1	**-1.4**	1350	12.2	62	1846	1551	23.5	62	C	6	021PAGNE	splicing factor, arginine/ serine-rich (transformer 2 Drosophila homolog) 10	Passed	AL560128 Entrez UniGene	2457759	pI NCY
294	2298	-1.2	**-1.4**	1343	8.9	48	1868	1570	18.4	48	E	11	0216AGN8	Finkel-Biskis-Reilly murine sarcoma virus (FBR-MuSV) ubiquitously expressed (fox derived); ribosomal protein S30	Passed	AA31606 7 Entrez UniGene	2734906	pI NCY
295	1532	-1.2	**-1.4**	1275	12.9	73	1756	1476	26.4	73	G	3	0215AGMC	Homo sapiens, clone MGC:18203 IMAGE:4155840, mRNA, complete cds	Passed	AW24828 3 Entrez UniGene	2135596	pI NCY
296	1866	-1.2	**-1.4**	1264	10.2	100	1743	1465	23.2	100	E	11	021VAGMQ	keratin 7	Passed	AA30737 3 Entrez UniGene	1649959	pI NCY

297	2641	-1.1	**-1.4**	1239	10.4	100	1677	1409	20.3	100	G	2	0215AGKO	crystallin, alpha B	Passed	BF727296 Entrez UniGene	3016305	pINCY
298	1348	-1.2	**-1.4**	1185	9.5	66	1627	1367	20.0	66	A	7	021FAGM5	albumin	Passed	N24732 Entrez UniGene	3354436	pINCY
299	1555	-1.2	**-1.4**	1157	9.2	100	1584	1331	19.7	100	G	1	021CAGMD	serine/ threonine kinase 12	Passed	BG616089 Entrez UniGene	161207	pBlue
300	7956	-1.2	**-1.4**	177	2.3	82†	245	206	3.5	82	F	12	021WAGKX	era (E. coli G-protein homolog)-like 1	Passed	BE871890 Entrez UniGene	1686892	pINCY

† Probe 1 did not meet selection criteria

Order LifeArray clones

Previous 100 Next 100

1 2 **3** 4 5 6 7 8 9 10 11 12 13 14 15 16 17 18 19 20 21 22 23 24 25 26 27 28 29 30 31 32 33 34 35 36 37 38 39 40 41 42 43 44 45 46 47 48 49 50 51 52 53 54 55 56 57 58 59 60 61 62 63 64 65 66 67 68 69 70 71 72 73 74 75 76 77 78 79 80 81 82 83 84 85 86 87 88 89 90 91 92 93 94

Entire List in plain text (long -- 1.91 MB)

PDF image of LifeArray (long -- 2.43 MB)

LifeArray color bar:

To save your LifeArray™ results on your computer, use the 'Plain Text' option to display your results, then save them on your computer with your browser's save feature. We will also provide your LifeArray results on a CD-ROM for a nominal fee. Please contact our Technical Support group if you need any assistance.

We guarantee that your LifeArray results will remain on the server for 90 days after it was first uploaded. After that, we may remove and archive your LifeArray results at our

discretion. Please contact our Technical Support group if you need any archived LifeArray results restored to our server.

In order to view or print Adobe® Acrobat® PDF files, you need the Adobe Acrobat Reader. If you do not already have it installed, you can obtain it for free from the Adobe web site .

If you have questions about the documents or have difficulty downloading the Acrobat Reader, please contact us.

Download the LifeArray Frequently Asked Questions list in HTML format.

Download the Human UniGEM V Frequently Asked Questions list in HTML format.

Download the LifeArray Control Plate Document in HTML format.

Adobe and Acrobat are trademarks of Adobe Systems Incorporated.

Sort Again:

Username: nature

Password:

Sort Order: Ascending Descending

Sort By:
Location
Diff Expr
Balanced Diff Expr
P1 Signal
P1 S/B
P2 Balanced Signal
P2 Signal
P2 S/B
Plate ID/Row/Col
Gene Name

Plate ID:

Gene Name:

LifeArray Products
Incyte Genomics Reagents Home
